# Semisynthetic teicoplanin derivatives with dual antimicrobial activity against SARS-CoV-2 and multiresistant bacteria

**DOI:** 10.1038/s41598-022-20182-y

**Published:** 2022-09-26

**Authors:** Ilona Bereczki, Vladimir Vimberg, Eszter Lőrincz, Henrietta Papp, Lajos Nagy, Sándor Kéki, Gyula Batta, Ana Mitrović, Janko Kos, Áron Zsigmond, István Hajdú, Zsolt Lőrincz, Dávid Bajusz, László Petri, Jan Hodek, Ferenc Jakab, György M. Keserű, Jan Weber, Lieve Naesens, Pál Herczegh, Anikó Borbás

**Affiliations:** 1 Department of Pharmaceutical Chemistry, University of Debrecen, Debrecen, Egyetem tér 1, 4032 Hungary; 2 National Laboratory of Virology, University of Pécs, Pecs, Ifjúság útja 20, 7624 Hungary; 3 Laboratory for Biology of Secondary Metabolism, Institute of Microbiology, Academy of Sciences of the Czech Republic, BIOCEV, Průmyslová 595, 252 50 Vestec, Czech Republic; 4 Institute of Healthcare Industry, University of Debrecen, Debrecen, Nagyerdei körút 98, 4032 Hungary; 5 Doctoral School of Pharmaceutical Sciences, University of Debrecen, Debrecen, Egyetem tér 1, 4032 Hungary; 6 Faculty of Sciences, Institute of Biology, University of Pécs, Pecs, Ifjúság útja 6, 7624 Hungary; 7 Department of Applied Chemistry, University of Debrecen, Debrecen, 4032 Hungary; 8 Department of Organic Chemistry, University of Debrecen, Debrecen, 4032 Hungary; 9 Department of Biotechnology, Jožef Stefan Institute, Jamova 39, 1000 Ljubljana, Slovenia; 10 Faculty of Pharmacy, University of Ljubljana, Aškerčeva cesta 7, 1000 Ljubljana, Slovenia; 11 TargetEx Ltd., Dunakeszi, Madách Imre utca 31/2, 2120 Hungary; 12 Medicinal Chemistry Research Group, Research Centre for Natural Sciences, Budapest, Magyar tudósok krt. 2, 1117 Hungary; 13 Institute of Organic Chemistry and Biochemistry, Czech Academy of Sciences, Flemingovo nam. 2, 16000 Prague 6, Czech Republic; 14 Rega Institute for Medical Research, KU Leuven, 3000 Leuven, Belgium

**Keywords:** Drug discovery and development, Chemical synthesis

## Abstract

Patients infected with SARS-CoV-2 risk co-infection with Gram-positive bacteria, which severely affects their prognosis. Antimicrobial drugs with dual antiviral and antibacterial activity would be very useful in this setting. Although glycopeptide antibiotics are well-known as strong antibacterial drugs, some of them are also active against RNA viruses like SARS-CoV-2. It has been shown that the antiviral and antibacterial efficacy can be enhanced by synthetic modifications. We here report the synthesis and biological evaluation of seven derivatives of teicoplanin bearing hydrophobic or superbasic side chain. All but one teicoplanin derivatives were effective in inhibiting SARS-CoV-2 replication in VeroE6 cells. One lipophilic and three perfluoroalkyl conjugates showed activity against SARS-CoV-2 in human Calu-3 cells and against HCoV-229E, an endemic human coronavirus, in HEL cells. Pseudovirus entry and enzyme inhibition assays established that the teicoplanin derivatives efficiently prevent the cathepsin-mediated endosomal entry of SARS-CoV-2, with some compounds inhibiting also the TMPRSS2-mediated surface entry route. The teicoplanin derivatives showed good to excellent activity against Gram-positive bacteria resistant to all approved glycopeptide antibiotics, due to their ability to dually bind to the bacterial membrane and cell-wall. To conclude, we identified three perfluoralkyl and one monoguanidine analog of teicoplanin as dual inhibitors of Gram-positive bacteria and SARS-CoV-2.

## Introduction

Since the emergence of severe acute respiratory syndrome coronavirus 2 (SARS‐CoV‐2) in December 2019, the pandemic has, so far, caused an estimated number of 537 million cases of COVID-19 and 6.3 million deaths worldwide. Even in countries with high vaccination coverage, SARS-CoV-2 continues to swiftly spread, which is related to rapidly waning immunity and frequent appearance of new virus variants. Antiviral drugs are clearly needed to enable early treatment of SARS-CoV-2-infected individuals at risk of severe disease. A few drug classes are already approved in some countries, i.e. antibodies targeting the viral spike protein (with often variant-dependent activity) and small-molecule inhibitors of the viral 3CL^Pro^ protease (Paxlovid®)^1^ or viral polymerase (molnupiravir and remdesivir)^2,3^.

Earlier investigations from our team revealed that lipophilic derivatives of some glycopeptide antibiotics combine favorable antibacterial with antiviral activity^4–7^, a property that is very attractive for clinical development. The dual activity of some of these molecules against two respiratory viruses, influenza virus and coronavirus, further enhances their potential^8^. One important glycopeptide drug is teicoplanin, an antibiotic with few side-effects and long plasma half-life, that is routinely used against multidrug-resistant Gram-positive bacteria such as methicillin-resistant *Staphylococcus aureus* (MRSA). Our group was the first to investigate the efficacy of teicoplanin against SARS-CoV-2 replication in cell culture^9^. Early in the COVID-19 pandemic, teicoplanin was proposed for dual use against SARS-CoV-2 and *Staphylococcus aureus* superinfection^10^. In a cohort of critically ill patients infected with SARS-CoV-2, Ceccarelli et al*.*^11^ observed that teicoplanin was effective at preventing Gram-positive superinfections but a significant antiviral effect was not seen. Although unmodified teicoplanin thus seems unable to exert dual antimicrobial activity in the clinic, this molecule remains a relevant lead structure to design more potent derivatives bearing appropriate modifications.

Mechanistically, the finding that teicoplanin efficiently suppressed SARS-CoV-2 pseudovirus entry into cells^10^ supports that its antiviral effect is related to inhibition of the SARS-CoV-2 entry process. Upon binding of the viral spike protein to the angiotensin-converting enzyme 2 (ACE2) receptor, the virion can enter via two routes: fusion at the cell surface or endocytosis followed by fusion in the endosome. Whereas the first route is favored in cells expressing high levels of TMPRSS2 (transmembrane serine protease 2), the endosomal pathway is functional in cells lacking this protease but rich in cathepsin B/L proteases^12,13^. In both routes, proteolytic cleavage is required to release the fusion peptide within the spike protein. The inhibitory effect of some glycopeptide antibiotics (i.e. teicoplanin, dalbavancin, oritavancin and telavancin) in a SARS-CoV-1 pseudovirus entry assay was attributed to inhibition of cathepsin L^14^. On the other hand, teicoplanin was also reported to inhibit SARS-CoV-2 3CL^Pro^ protease in an enzymatic assay, suggesting that it may target different steps in the viral replication cycle^15^. This is consistent with our own findings, showing that teicoplanin and its more potent derivatives bearing lipophilic apocarotenoid side chains, suppress SARS-CoV-2 replication by combining inhibition of cathepsin L and 3CL^Pro^^9^. The derivatives retained antibacterial activity against MRSA and enterococcal strains.

**Figure 1:**
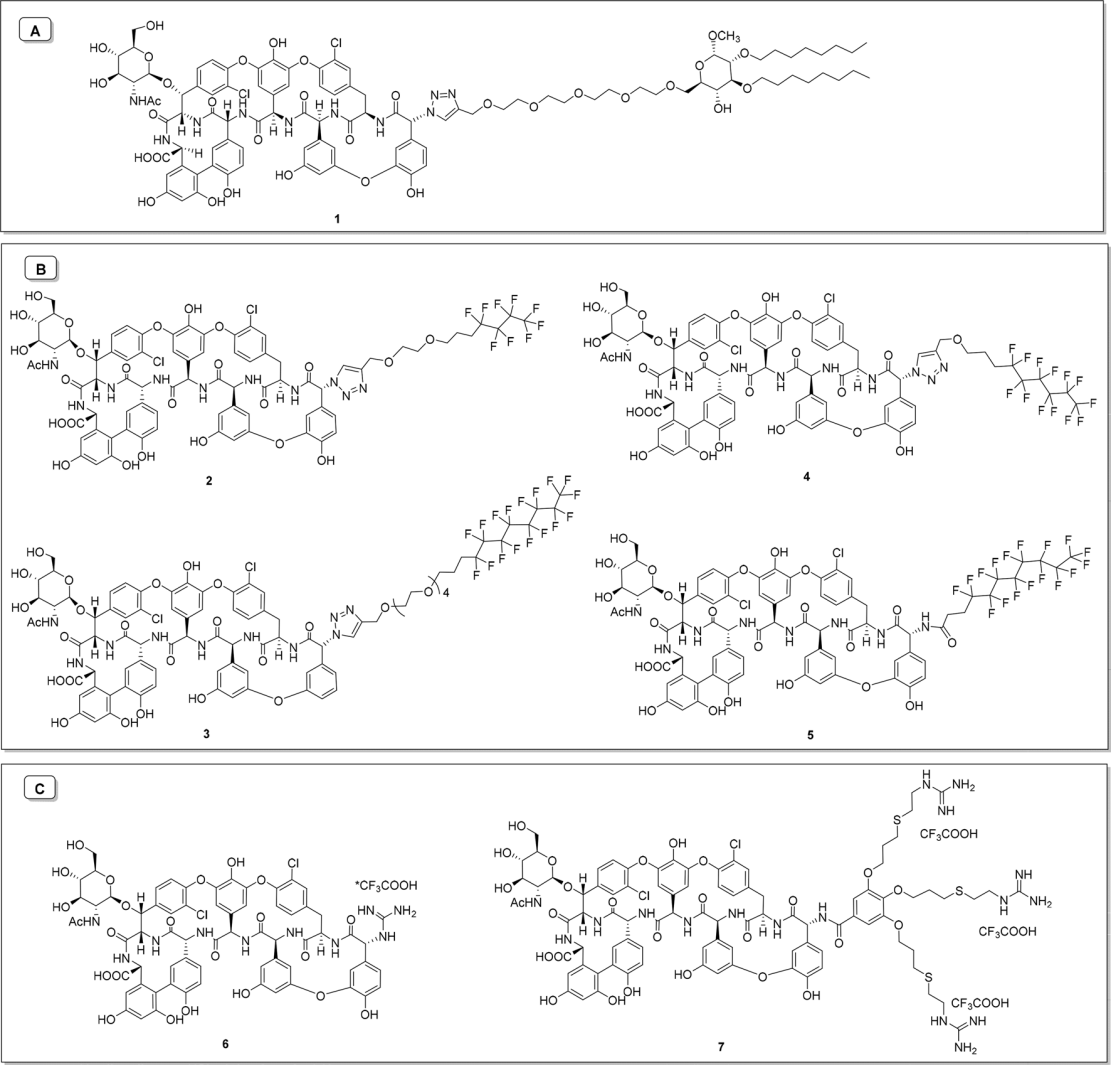
Teicoplanin pseudoaglycone derivatives bearing hydrophobic (**A**), dually lipo- and hydrophobic (**B**) and superbasic guanidino (**C**) groups at the *N*-terminal amino group.

Over the past decade, we and others have amply demonstrated that the antibacterial or antiviral activity of glycopeptide antibiotics can be significantly enhanced by synthetic modifications^16–19^. However, only certain synthetic modifications have produced significant combined effects against both types of pathogens, i.e. viruses and bacteria. We showed that attaching teicoplanin pseudoaglycone to a double-tailed lipophilic group^7,8^ or dually lipo- and hydrophobic perfluoroalkyl chain^20^, leads to favorable anti-RNA virus or antibacterial activity, depending on the length of the chain. Furthermore, introducing a superbasic guanidino group to the peptide core of teicoplanin confers excellent activity against glycopeptide-resistant staphylococci and enterococci^21^. We hypothesized that combining the above-mentioned three types of synthetic modifications might deliver semisynthetic teicoplanins with dual activity against SARS-CoV-2 and bacteria, a highly relevant feature for management of COVID-19. To test this hypothesis, we selected prototypes of previously prepared teicoplanin derivatives equipped with double lipophilic tails (**1**^7^); perfluoroalkyl chains (**2** and **3**)^20^; or a guanidine group (**6**^21^), besides preparing three new analogs (**4**, **5** and **7**) (Fig. 1). A thorough investigation was performed to determine their activity against SARS-CoV-2 and multidrug-resistant bacteria, and underlying biological mechanisms.

## Results and discussion

### Synthesis

Compound **1** was selected on the basis of its high anti-RNA virus activity^7,8^. From the perfluoroalkyl-substituted series described previously^20^. we selected derivative **2** with perfluorobutyl chain and compound **3** with perfluorooctyl chain. Compound **2** exerted impressive activity (MIC 0.5–1 µM) against a panel of bacteria including MRSA, and also showed broad antiviral activity, though not against human coronavirus 229E (HCoV-229E) (EC_50_ > 100 µM). On the other hand, **3** was found to have modest antibacterial effect (MIC 4–32 µM) but nice activity against HCoV-229E (EC_50_: 4.9 µM)^20^. In order to study how the antiviral and antibacterial activity can be tuned by modifying the length of the perfluoroalkyl chain or the linker region, we designed compound **4** containing a medium-length perfluorohexyl chain, and derivative **5** in which the triazole ring and tetraethylene moiety of the linker region are omitted. Compound **6** was already found to have robust antibacterial activity^21^, but was so far not evaluated for antiviral activity. Furthermore, the triguanidine derivative **7** was prepared to assess whether the biological activity is enhanced by multiplying the guanidine groups.

**Figure 2:**
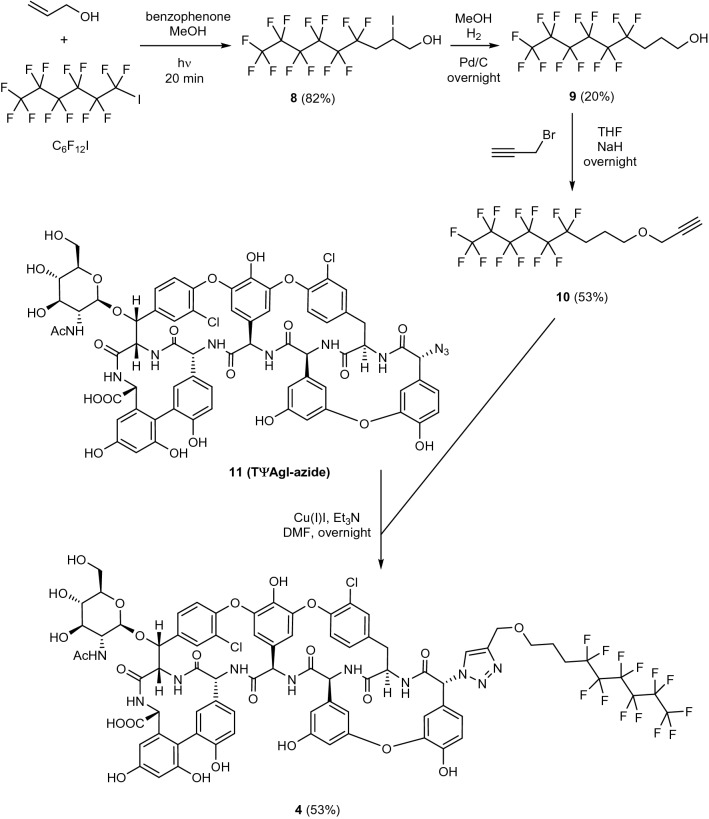
Synthesis of teicoplanin pseudoaglycon derivative **4** bearing a perfluoroalkyl chain of medium-length.

We started our work with the synthesis of teicoplanin derivative **4** bearing a perfluorohexyl mioety (Fig. 2). A light-promoted atom-transfer radical addition reaction^22^ of the commercially available perfluorohexyl iodide onto the double bond of allyl alcohol resulted in **8**, and removal of its iodo subtstituent by catalytic hydrogenation afforded **9** with a moderate yield. Reaction of the latter with propargyl bromide produced **10**, which was conjugated to teicoplanin pseudoaglycone azide **11**^5^ by azide alkyne cycloaddition click reaction to provide the required **4** carrying a perfluorohexyl side chain attached to the peptide core via a triazole ring.

**Figure 3:**
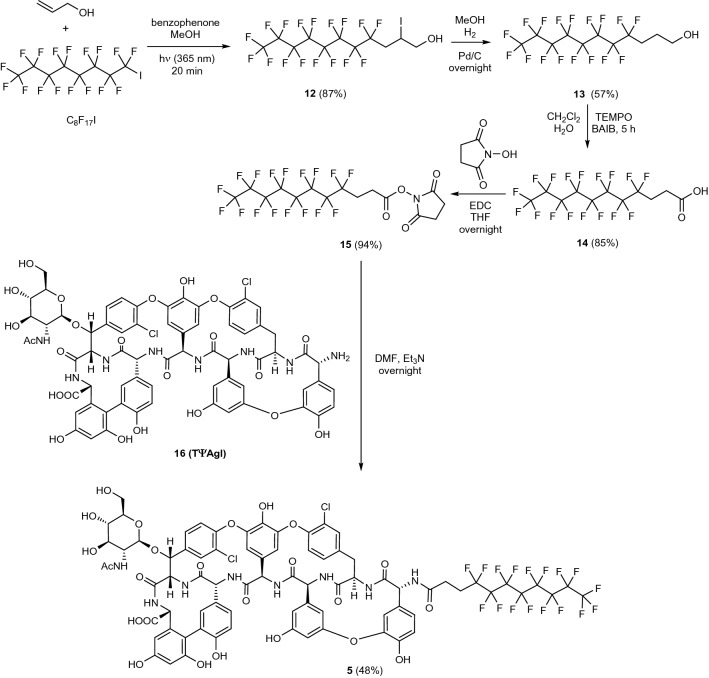
Attachment of a perfluorooctyl side chain to the *N*-terminal amino group of the peptide core via acylation.

To elucidate how the biological activity of **3** is dependent on the linker region, consisting of a triazole ring and tetraethylene glycol unit, we prepared a related perfluorooctyl derivative (**5**) by a simple *N*-acylation reaction (Fig. 3). Alcohol **13** was prepared via photoinduced addition of perfluorooctyl iodide to allyl alcohol followed by catalytic hydrogenation. Next, TEMPO-BAIB mediated oxidation of **13** resulted in the carboxylic acid derivative **14** which was then converted to active ester **15** and used for *N*-acylation of teicoplanin pseudoaglycone **16**^21,23^ to produce **5** in which the perfluorooctyl chain is attached to the peptide core through an amide bond.

**Figure 4:**
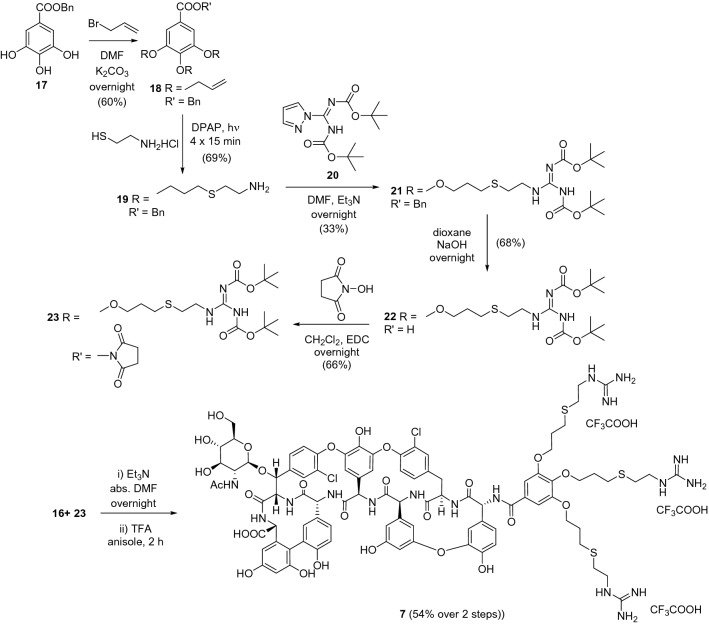
Synthesis of compound **7**.

For the synthesis of **7** bearing three guanidine moieties, benzyl gallate was reacted with allyl bromide to obtain the trivalent scaffold **18** (Fig. 4). UV-light induced radical addition^24^ of cysteamine hydrochloride in the presence of the photoinitiator 2,2-dimethoxy-2-phenylacetophenone (DPAP) onto the double bonds of **18** afforded **19** displaying three primary amino groups. Formation of the guanidine moities was achieved by reacting **19** with the readily available 1-pyrazolecarboxamidine reagent **20**. Carboxylic acid function of triguanidine derivative **21** obtained was deprotected by catalytic hydrogenolysis (**22**) and converted to active ester **23** by treatment with *N*-hydroxysuccinimide in the presence of EDC. Finally, acylation of the primary amino group of teicoplanin pseudoaglycone **16** with **23** produced **7** displaying three guanidine groups at the *N*-terminal amino acid.

**Figure 5:**
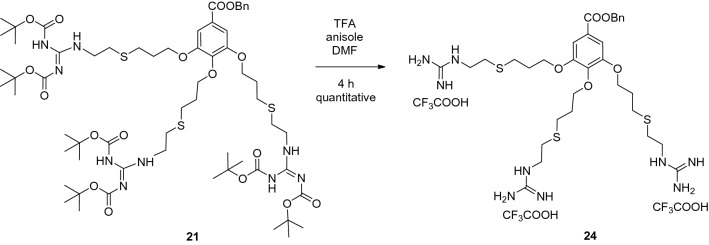
Synthesis of side chain **24** for antiviral study.

In order to test whether the triple guanidinylated side chain of **7** alone has antiviral activity, benzyl gallate derivative **24** (Fig. 5) was prepared and included in the antiviral evaluation.

### Anti-coronavirus evaluation

#### Activity in cellular assays

We determined the anti-coronavirus activity by several cell-based methods: cytopathic effect (CPE) reduction assays with SARS-CoV-2 in VeroE6 and Calu-3 cells, and with HCoV-229E in HEL cells; and a SARS-CoV-2 pseudovirus entry assay performed in Vero and A549-AT cells (i.e. A549 cells that express ACE2 and TMPRSS2). The results are summarized in Table 1.

**Table 1:** Inhibitory activity on coronavirus replication and spike-mediated pseudovirus entry.

**Compound**	Activity and cytotoxicity in CPE reduction assays (µM)						Inhibition of SARS-CoV-2 pseudovirus entry in	
	SARS-CoV-2 in Vero E6 cells^a^		SARS-CoV-2 in Calu-3 cells^b^		HCoV-229E in HEL cells^c^		Vero cells	A549-AT^d^ cells
	EC_50_	CC_50_	EC_50_	CC_50_	EC_50_^e^	CC_50_^e^	EC_50_^f^ (µM)	EC_50_^f^ (µM)
**1**	25 ± 2.5	> 100	63 ± 7.4	75 ± 9.0	17	> 100	2.2 ± 0.1	11 ± 5
**2**	> 100	> 100	nd	nd	> 100	> 100	nd	nd
**3**	28 ± 1.9	> 100	49 ± 3.4	> 100	4.9	> 100	nd	nd
**4**	13 ± 1.8	> 100	22 ± 0.6	93 ± 11	12	> 100	1.0 ± 0.2	16 ± 4
**5**	24 ± 2.4	> 100	57 ± 2.6	> 100	13	> 100	nd	nd
**6**	13 ± 1.6	> 100	> 100	> 100	≥ 83	> 100	14 ± 3	> 125
**7**	53 ± 12	> 100	> 100	> 100	> 100	> 100	nd	nd
**Teicoplanin**	16 ± 1.7	> 100	> 100	> 100	> 100	> 100	21 ± 5	> 125
**9**	> 100	> 100	nd	nd	nd	nd	nd	nd
**13**	> 100	> 100	nd	nd	nd	nd	nd	nd
**14**	19 ± 1.5	39 ± 1.4	46 ± 1.9	~ 53	69	> 100	13 ± 5	24 ± 6
**24**	> 100	33.2	nd	nd	nd	nd	nd	nd
**Remdesivir**^**g**^	2.5 ± 0.2	> 100	0.3 ± 0.03	> 50	–	–	–	–
**GS-441524**^** g**^	–	–	–	–	3.0	> 40	–	–
**Camostat**^**g**^	–	–	–	–	–	–	> 20	0.49 ± 0.05
**E64d**^**g**^	–	–	–	–	–	–	1.3 ± 1.2	> 20

^a^VeroE6: African green monkey kidney cells.

^b^Calu-3: human lung adenocarcinoma cells.

^c^HEL: human embryonic lung fibroblast cells.

^d^A549-AT: human lung cancer cells expressing ACE2 and TMPRSS2.

^e^EC_50_ (50% effective concentration) and CC_50_ (50% cytotoxic concentration) based on MTS-based cell viability assay. Values are the mean of 2–3 tests.

^f^EC_50_: concentration at which the luciferase signal was 50% compared to the condition receiving no compound. Values are the mean ± SEM of 3 tests.

nd: not determined.

^g^Reference compounds: remdesivir, nucleotide prodrug inhibitor of coronavirus RNA synthesis; GS-441524, nucleoside form of remdesivir; camostat: inhibitor of serine proteases like TMPRSS2; E64d: inhibitor of cathepsins like the endo/lysosomal cathepsin L enzyme.

The African green monkey kidney VeroE6 cell line exhibits strong CPE when infected with SARS-CoV-2^25^. Due to the lack of TMPRSS2, the virus enters these cells by the endosomal route in which the spike is activated by cathepsin L. Except for perfluorobutyl conjugate **2**, all teicoplanin derivatives proved effective at reducing the CPE of SARS-CoV-2. Lipophilic derivative **1**, perfluoroalkyl conjugates **3–5**, guanidine derivative **6** and unmodified teicoplanin showed EC_50_ values in the range of 10–20 µM, while the triguanidine-modified derivative **7** had an EC_50_ of 53 µM. The modifying groups **9**, **13** and **24** were, on their own, not active. The fluorous carboxylic acid derivative **14** did show antiviral activity, although it was also quite toxic.

We also tested the compounds in human Calu-3 cells, a lung cancer-derived epithelial cell line that expresses TMPRSS2 and supports virus entry via the cell surface pathway^25^. The teicoplanin conjugates **1**, **3**, **4** and **5** and modifying group **14** proved again active, with compound **4** having an EC_50_ value of 20 µM. Unmodified teicoplanin and the two guanidine derivatives **6** and **7** were inactive.

Finally, we tested the compounds against HCoV-229E, a common cold coronavirus. While unmodified teicoplanin, **2** and **7** were inactive and **6** showed only modest activity, conjugates **1**, **3**, **4** and **5** produced robust inhibition of HCoV-229E CPE, with EC_50_ values of 17, 4.9, 12 and 13 µM, respectively. A clear parallel was visible between activity in SARS-CoV-2-infected Calu-3 cells and HCoV-229E-infected HEL cells.

**Figure 6:**
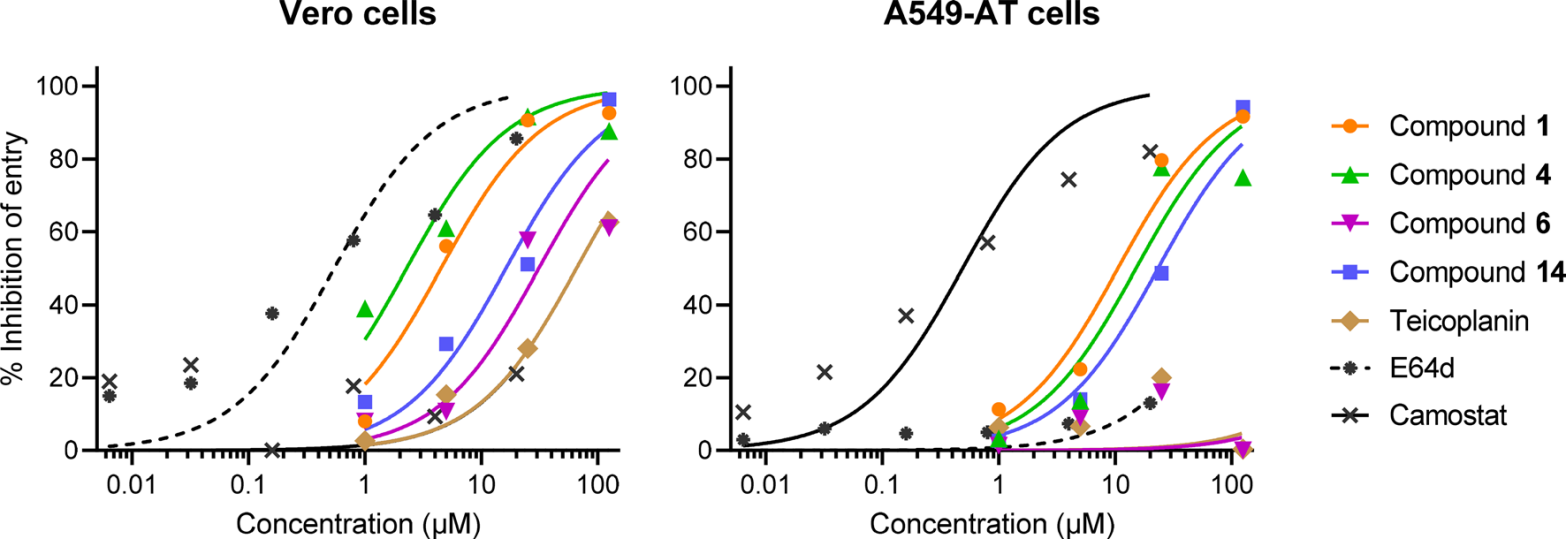
Dose–response curves for inhibition of SARS-CoV-2 pseudovirus entry in Vero (left) and A549-AT (right) cells. The data points are the average values of three independent experiments.

Next, we submitted a selection of five compounds to pseudovirus assays, in order to determine inhibition of spike-mediated entry by the cathepsin-dependent endosomal pathway (active in Vero cells) and TMPRSS2-mediated uptake at the cell surface (in A549-AT cells). Teicoplanin conjugates **1** and **4** proved highly effective in Vero cells (EC_50_ values: 1 and 2 µM, respectively) and also active, though less potent, in A549-AT cells (Fig. 6). Unmodified teicoplanin and guanidine derivative **6** were moderately active in Vero cells but inactive in A549-AT cells. The modifying group **14** inhibited both entry routes.

In combination, these cell-based data show that the glycopeptide derivatives suppress SARS-CoV-2 replication in VeroE6 cells (CPE assay) by inhibiting virus entry via the endosomal pathway (pseudovirus assay). Additional activity against the surface entry route was accomplished by creating teicoplanin conjugates **1** and **4**, explaining their efficacy in Calu-3 cells. Comparison of compounds **2** and **4** shows that the antiviral activity is determined by the length of the perfluoroalkyl chain. These coronavirus entry inhibitors are not restricted to SARS-CoV-2 since also HCoV-229E proved sensitive. Finally, we found that unmodified teicoplanin does not inhibit SARS-CoV-2 in Calu-3 cells, a cell model in which this glycopeptide antibiotic was not evaluated so far.

#### Activity in biochemical assays

Our recent work on teicoplanin analogs with apocarotenoid side chains pointed to cathepsin L inhibition as the main mechanism, with inhibition of the viral 3CL^Pro^ enzyme as an auxiliary mechanism^9^. Another group reported that the structurally related glycopeptide dalbavancin binds to the ACE2 receptor, thereby preventing interaction between ACE2 and the receptor-binding domain (RBD) of the viral spike protein^26^. Hence, we tested our teicoplanin conjugates **1**–**7** in three biochemical assays to measure inhibition of cathepsin L or 3CL^Pro^ enzyme activity, or the ACE2-spike interaction (Table 2).

**Table 2:** Inhibition of SARS-CoV-2-related entry factors or the viral 3CL^pro^ enzyme.

**Compound**	Cathepsin L	ACE2-spike interaction	3CL^Pro^
	IC_50_ (µM)	% inhibition at 100 µM	% inhibition at 200 µM
**1**	49 ± 1	1.4 ± 6.7	43 ± 10
**2**	98 ± 1	16 ± 5	13 ± 7
**3**	33 ± 1	22 ± 0	16 ± 2
**4**	66 ± 1	7.6 ± 12.7	9 ± 14
**5**	52 ± 1	5.0 ± 4.1	65 ± 2
**6**	> 500	28 ± 12	48 ± 2
**7**	4.5 ± 1.0	52 ± 2	38 ± 8
**Teicoplanin**	5% at 50 µM	34 ± 7	13 ± 8
**14**	> 500	5.7 ± 5.3	12 ± 2
**24**	18 ± 1	7.4 ± 12.0	− 14 ± 1^a^

^a^Raw fluorescence values are converted to percent inhibition values by normalization between 0 and 100% with the negative and positive controls, respectively. Small negative values are within the precision limit of the measurement and can be considered as a complete lack of activity.

In line with our previous study^9^, we observed inhibition of cathepsin L with IC_50_ values in the mid-micromolar range and as low as 4.5 µM for compound **7** (see Figure S15 for the dose–response curves). Unlike the structurally related analogs that we reported earlier^9^, compounds **1**–**7** exhibited no significant 3CL^Pro^ inhibition (< 50% inhibition at 200 µM; 65% inhibition for compound **5**). Also inhibition of the ACE2-spike interaction was weak, with only compound **7** reaching 50% inhibition at a concentration of 100 µM.

Hence, for the lipophilic derivative **1** and perfluoroalkyl conjugates **3**–**5**, inhibition of cathepsin L plausibly contributes to their anti-coronavirus effect in VeroE6 cells. For compound **1**, this is consistent with the literature results, which demonstrated that glycopeptide antibiotics containing a lipophilic group exert their anti-SARS-CoV-2 effect by interacting with the enzymatic domains of cathepsin L^9,14^. At the same time, the cathepsin inhibitory data of compounds **3**–**5** reveal that the lipophilic group is not essential for cathepsin inhibition, the perfluoroalkyl groups having a dual lipo- and hydrophobic character can also provide this effect. The anti-coronavirus activity of the lipophilic conjugate **1** and the perfluoroalkylated compounds **3**–**5** in Calu-3 cells lacking cathepsin L, can presumably be explained by their ability to also inhibit the TMPRSS2 protein. It is mportant to note that the inactivity of the perfluorobutyl derivative **2** observed in all antiviral assays clearly shows that the size of the hydrophobic group is crucial for the interaction with the proteins that mediate viral entry; among the tested side chains, the perfluorohexyl group seems to be optimal for eliciting antiviral activity.

For guanidine derivatives **6** and **7**, the suppression of endocytic virus entry showed an inverse relationship with the inhibitory effect on cathepsin L. Compound **6** did not prove to be an inhibitor of cathepsin L but efficiently prevented the endosomal pseudovirus entry, possibly by increasing the endosomal pH due to its highly basic guanidine moiety. The triguadinine-teicoplanin conjugate **7** might have insufficient cell permeability to exert antiviral activity.

### Antibacterial evaluation

We determined the minimal inhibitory concentrations (MIC) of the teicoplanin conjugates for a large panel of Gram-positive bacterial strains, using vancomycin, unmodified teicoplanin, dalbavancin and oritavancin as reference compounds. We used the broth microdilution method according to EUCAST ISO standard 20776-1 and the MIC results for the known glycopeptide antibiotics were read according to EUCAST clinical breakpoints (EUCAST, 2019). The bacterial strains (see Suppl. Table S5 for all details) were selected to achieve maximum diversity of multidrug-resistance and include strains with known mechanisms of resistance to glycopeptides in clinical use. The *S. epidermidis* and *S. haemolyticus* strains resistant to dalbavancin and oritavancin were obtained in vitro from clinical isolates sensitive to the glycopeptide antibiotics.

Except for compound **6**, all derivatives were inactive against *E. faecium* and *E. faecalis* strains expressing the VanA resistance phenotype (Table 3). This is the most common glycopeptide antibiotic resistance in enterococci. It causes cell wall reprogramming by enzymes encoded in *vanHAX* gene clusters, yielding peptidoglycan precursors with d-alanine-d-lactate (d-Ala-d-Lac) or d-alanine-d-serine instead of d-alanine-d-alanine (d-Ala-d-Ala) and decreasing the affinity of glycopeptide antibiotics for the peptidoglycan^27^. Alike oritavancin, the only approved glycopeptide antibiotic that can overcome VanA resistance, the monoguanidine derivative of teicoplanin **6** exhibited strong activity against our collection of *E. faecium* strains. This means that both compounds do not depend on modification of peptidoglycan precursors. Compound **6** proved also active against *S. epidermidis* and *S. haemolyticus* exhibiting resistance to all four glycopeptide antibiotics. Hence, **6** appears to exert its antibacterial action by another mechanism than oritavancin and dalbavancin.

**Table 3:** Activity against diverse species of Gram-positive bacteria including drug-resistant strains.

Bacterial species and phenotype	No. of strains tested^a^	Average MIC value (µg/ml) for compound^b^									
		2	3	4	5	6	7	VAN	TEI	DALB	ORI
***S. aureus***											
MSSA	4	0.265	2.5	1.75	1.75	0.1875	1.125	0.625	0.375	0.125	0.078
CA-MRSA	1	0.25	2	2	4	0.125	1	0.25	0.125	0.0625	0.0625
MRSA	7	0.285	2.285	1.714	2.57	0.125	0.89	1.25	1.43	0.1	0.14
***E. faecalis***											
VAN^S^, TEI^S^	2	1	> 8	0.25	0.25	0.125	2	0.25	0.25	0.25	0.0625
VAN^R^,TEI^R^/VanA	1	> 8	> 8	> 8	> 8	0.125	> 8	> 64	> 64	64	0.125
***E. faecium***											
VAN^S^, TEI^S^	1	1	> 8	0.25	0.25	0.0625	2	0.25	0.5	0.25	0.0625
VAN^R^,TEI^R^/VanA	8	> 8	> 8	> 8	> 8	0.15625	> 8	> 64	> 64	48	0.12
***S. epidermidis***											
mecA	6	0.645	2.75	0.812	1.4	0.1	0.25	6.67	26.7	1.77	1.2
***S. haemolyticus***											
mecA	6	1.9	7	6.8	7	0.27	1.2	28	112	3.83	0.24

^a^See Suppl. Table S5 for individual MIC values of each tested strain.

^b^VAN: vancomycin; TEI: teicoplanin; DALB: dalbavancin, ORI: oritavancin. ^S^ sensitive; ^R^ resistant.

Among the bacteria causing superinfection in hospitalized COVID-19 patients, there are also Gram-negative pathogens (most commonly *Klebsiella pneumoniae*). Given the well-known membrane permeabilizing ability of guanidinium ions^21^, we hypothesized that teicoplanin derivatives **6** and **7** bearing one and three guanidine groups, respectively, may be effective against Gram-negative strains. Unfortunately, however, they were inactive against the four clinically relevant Gram-negative bacterial strains tested (Table S6).

#### Fluorescent binding assays

To further clarify the antibacterial properties, we used our fluorescent assay^28^ to assess the compounds’ ability to reverse the binding of vancomycin and teicoplanin to the surface of authentic *S. aureus*. Vancomycin exerts its antibacterial effect by binding to the cell-wall peptidoglycan precursor d-Ala-d-Ala, leading to physical inhibition of the bacterial transpeptidase enzyme^29^. Teicoplanin has a more complex and not fully elucidated mechanism^29,30^, that involves attachment to the bacterial membrane via the lipophilic fatty acid side chain^31^.

**Figure 7:**
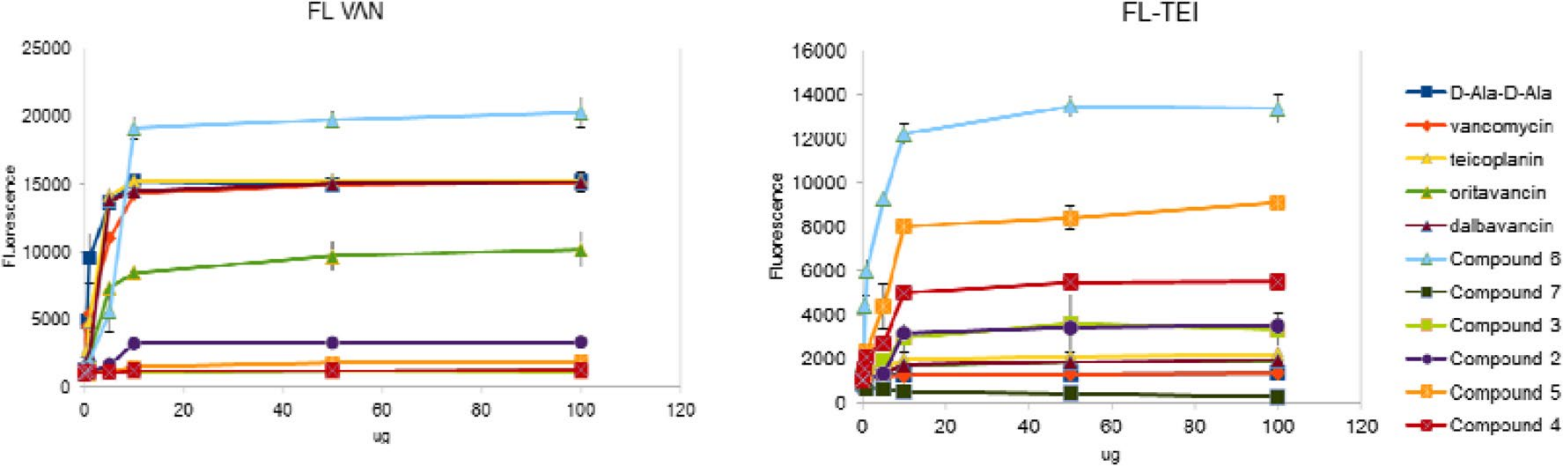
Fluorescent competition assay for glycopeptide binding to *S. aureus.* Exponentially growing bacterial cells were saturated with fluorescent vancomycin (left panel) or teicoplanin (right panel). Following addition of d-Ala-d-Ala or the glycopeptide compounds, the release of fluorescent vancomycin or teicoplanin was measured by Tecan Infinite 200Proreader. Data points are the average ± SEM values of 3 independent tests.

The results show that monoguanidine **6** was highly effective at outcompeting fluorescent vancomycin (Fig. 7, left panel), indicating excellent binding affinity for the bacterial cell wall. The triguanidine derivative **7** and perfluoroalkyl compounds **2**–**5** had no effect in this assay. Unmodified teicoplanin and dalbavancin outcompeted fluorescent vancomycin with similar potency as non-fluorescent vancomycin and the dipeptide d-Ala-d-Ala. For oritavancin, the competition was less pronounced. In the binding assay with fluorescent teicoplanin (Fig. 7, right panel), conjugate **6** was again the most active competitor, which is probably due to electrostatic interaction between its cationic guanidine group and the negatively charged bacterial membrane. The perfluoroalkyl derivatives **2**–**5** outcompeted fluorescent teicoplanin with higher efficacy than unlabeled teicoplanin itself, indicating that these conjugates exhibit superior binding to the cell membrane.

Overall, the results with derivative **6** demonstrate that the highest antibacterial activity is achieved when a glycopeptide antibiotic combines binding to the cell wall precursors with affinity for the cell membrane. This property of **6** explains its activity against *Staphylococcus* strains that are even resistant to oritavancin. The perfluoroalkyl-modified teicoplanin derivatives **4** and **5** bind to the membrane but not to cell wall precursors, explaining their lower antibacterial activity. For vancomycin, teicoplanin and dalbavancin, high efficiency binding was only seen for peptidoglycan precursors, explaining why these drugs cannot overcome modification of the d-Ala-d-Ala unit in bacteria bearing the VanA resistance marker.

## Conclusion

Among a series of seven teicoplanin derivatives, the perfluoroalkyl conjugates **3**–**5** and guanidine derivative **6** were found to combine promising anti-SARS-CoV-2 activity with outstanding antibacterial activity. Perfluorohexyl analog **4** emerged as the most potent inhibitor. Dual activity against SARS-CoV-2 and Gram positive bacteria is a property with high relevance for management of COVID-19 disease bearing a risk of bacterial superinfection.

Mechanistically, these teicoplanin conjugates inhibit the SARS-CoV-2 spike-mediated entry process, particularly the endosomal route that is dependent on cathepsin L. Based on our enzymatic findings, we propose that direct inhibition of cathepsin L contributes to the anti-coronavirus effect. However, also other mechanisms apply, given that unmodified teicoplanin and the guanidine conjugate **6** proved active against SARS-CoV-2 in VeroE6 cells despite being inactive on cathepsin L enzyme. In addition, the lipophilic conjugate **1** and perfluoroalkylated derivatives **3**–**5** exhibited modest inhibition of virus entry via the TMPRSS2-mediated cell surface route, in Calu-3 cells infected with live virus and A549-AT cells infected with pseudovirus. The basis for this effect is currently unknown, but inhibition of the ACE2-spike interaction was excluded.

Regarding the antibacterial effect, the high potency of guanidine derivative **6** across all (drug-resistant) bacterial strains in the test panel proved to be related to its strong binding affinity to the cell wall precursor dipeptide but also the bacterial membrane.

To conclude, the three different synthetic modifications (double lipophilic tail, perfluoroalkyl chains, and guanidine group) studied, the incorporation of either a guanidine moiety or a perfluorooctyl/hexyl group to the peptide core effectively increased the activity of the parent teicoplanin antibiotic against both SARS-CoV-2 and resistant bacteria. Further testing of the compounds against different variants of SARS-CoV-2 will decide which modification provides the most potent lead compound for the development of a dual-acting drug for the treatment of CoVID-19.

## Experimental

### General informations

TLC was performed on Kieselgel 60 F254 (Merck) with detection either by immersing into ammonium molybdate-sulfuric acid solution followed by heating or by using Pauly’s reagent for detection. Flash column chromatography was performed using Silica gel 60 (Merck 0.040–0.063 mm). The ^1^H NMR (500 and 400 MHz) ^13^C NMR (125 and 100 MHz) and 2D NMR spectra were recorded with a Bruker DRX-400 and Bruker Avance II 500 spectrometer at 298 K or 300 K. Chemical shifts are referenced to Me_4_Si (0.00 ppm for 1H) and to the solvent residual signals. MALDI-TOF MS measurements were carried out with a Bruker Autoflex Speed mass spectrometer equipped with a time-of-flight (TOF) mass analyzer. In all cases 19 kV (ion source voltage 1) and 16.65 kV (ion source voltage 2) were used. For reflectron mode, 21 kV and 9.55 kV were applied as reflector voltage 1 and reflector voltage 2, respectively. A solid phase laser (355 nm, ≥ 100 μJ/pulse) operating at 500 Hz was applied to produce laser desorption and 3000 shots were summed. 2,5-Dihydroxybenzoic acid (DHB) was used as matrix and F_3_CCOONa as cationising agent in DMF. ESI-QTOF MS measurements were carried out on a maXis II UHR ESI-QTOF MS instrument (Bruker), in positive ionization mode. The following parameters were applied for the electrospray ion source: capillary voltage: 3.5 kV; end plate offset: 500 V; nebulizer pressure: 0.8 bar; dry gas temperature: 200 °C and dry gas flow rate: 4.5 L/min. Constant background correction was applied for each spectrum, the background was recorded before each sample by injecting the blank sample matrix (solvent). Na-formate calibrant was injected after each sample, which enabled internal calibration during data evaluation. Mass spectra were recorded by otofControl version 4.1 (build: 3.5, Bruker) and processed by Compass DataAnalysis version 4.4 (build: 200.55.2969). For analytical RP-HPLC a Waters 2695 Separations Module (Waters Corp., Milford, USA) was used. The separations were carried out on a VDSphere PUR 100 C18-M-SE, 5 µm, 150 × 4.6 mm column at an injection volume of 10 µL, using a flow rate of 1.0 mL/min with a Waters 2996 DAD set at 254 nm and a Bruker MicroTOF-Q type Qq-TOF MS instrument (Bruker Daltonik, Bremen, Germany) as detectors. All compounds are > 95% pure by HPLC analysis. The following system was used for the elution: Solvent A: water: MeCN 9:1 + 0.0025%v/v TFA and Solvent B: MeCN. Gradient elution: from 20% of B to 80% from 0 to 40 min and 80% of B from 40 to 50 min. The MicroTOF-Q mass spectrometer was equipped with an electrospray ion source. The mass spectrometer was operated in positive ion mode with a capillary voltage of 3.5 kV, an endplate offset of − 500 V, nebulizer pressure of 1.8 bar, and N_2_ as drying gas with a flow rate of 9.0 l/min at 200 °C. The mass spectra were recorded by means of a digitizer at a sampling rate of 2 GHz. The mass spectra were calibrated externally using the exact masses of clusters [(NaTFA)_n_ + TFA]^+^ from the solution of sodium trifluoroacetate (NaTFA). The spectra were evaluated with the DataAnalysis 3.4 software from Bruker. The photoinduced reactions were carried out in a borosilicate vessel by irradiation with a low-pressure Hg-lamp (Osram Supratec UV, HTC 150–211, 150 W, 230 V, R7s) giving maximum emission at 365 nm.

### Chemistry

#### Compound 8

Allyl alcohol (585 μl, 8.61 mmol) was dissolved in abs. methanol (5 mL) and perfluorohexyl iodide (2.24 mL, 10.33 mmol, 1.2 equiv.) and benzophenone (17 mg, 0.09 mmol) in abs. methanol (1 mL) were added. Argon gas was bubbled through the solution for 10 min and the reaction mixture was irradiated with UV light for 20 min. The solvent was evaporated and the product was purified by flash column chromatography (hexane/acetone 95:5) to yield **8** (3.54 g, 82%) as a white solid. *R*_f_ = 0.35 (hexane/acetone 9:1); ^1^H NMR (500 MHz, methanol-d_4_): *δ* (ppm) 4.30–4.22 (m, 1H, 2. C*H*I), 3.84 (dd, 1H, J = 11.9, 5.1 Hz, C-1 C*H*_2_a), 3.68 (dd, 1H, *J* = 11.9 Hz, 7.3 Hz, C-1 C*H*_2_b), 3.26–3.11 (m, 1H, C-3 C*H*_2_a), 2.79–2.63 (m, 1H, C-3 C*H*_2_b); ^13^C NMR (100 MHz, CDCl_3_): *δ* (ppm) 120.5, 118.8, 117.9, 115.9, 113.8, 111.1, 108.4 (6C, *C*F_2_ and *C*F_3_), 68.1 (1C, C-1 *C*H_2_), 37.6 (t, 1C, C-3 *C*H_2_), 21.8 (1C, *C*HI).

#### Compound 9

Compound **8** (3.54 g, 7.02 mmol) was dissolved in abs. methanol (20 mL) and argon gas was bubbled through the solution for 10 min. 10% palladium on activated charcoal (708 mg) and NaHCO_3_ (1.47 g, 17.56 mmol, 2.5 equiv.) were added, and the reaction mixture was stirred overnight under H_2_ atmosphere, then filtered through Celite, and the solvent was evaporated. The product was purified by flash column chromatography (hexane/acetone 9:1 and 8:2) to yield 536 mg (20%) of **9** as a colourless syrup. *R*_f_ = 0.34 (hexane/acetone 8:2); ^1^H NMR (500 MHz, CDCl_3_):* δ* (ppm) 3.75 (t, 2H, *J* = 6.1 Hz, C-1 C*H*_2_), 2.29–2.15 (m, 2H, C-2 C*H*_2_), 1.92–1.83 (m, 2H, C-3 C*H*_2_); ^13^C NMR (100 MHz, methanol-d_4_): *δ* (ppm) 120.0, 117.2, 116.1, 112.6 (6C, *C*F_2_ and *C*F_3_), 61.5 (1C, C-1 *C*H_2_), 28.6 (t, 1C, C-3 *C*H_2_), 24.4 (1C, C-2 *C*H_2_).

#### Compound 10

Compund **9** (200 mg, 0.53 mmol) was dissolved in abs. THF (2 mL) and the solution was cooled in an ice bath under argon atmosphere. NaH (50% dispersion in mineral oil) (51 mg, 1.06 mmol) was added and the mixture was stirred for 30 min, then propargyl bromide (80% solution in toluene) (57 μL, 0.636 mmol, 1.2 equiv.) was added. The mixture was stirred at room temperature overnight, then methanol (2 mL) and distilled water (1 mL) were added to the mixture and it was stirred for 15 min. The solvent was evaporated and the product was purified by flash column chromatography (hexane/acetone 99:1) to yield **10** (115 mg, 53%) as a colourless syrup. *R*_f_ = 0.78 (hexane/acetone 98:2); ^1^H NMR (400 MHz, CDCl_3_):* δ* (ppm) 4.18–4.13 (m, 2H, propargyl C*H*_2_) 3.6 (t, 2H, *J* = 6.0 Hz, C-1 C*H*_2_), 2.45–2.41 (m, 1H, propargyl C*H*), 2.29–2.11 (m, 2H, C*H*_2_), 1.95–1.86 (m, 2H, C*H*_2_); ^13^C NMR (100 MHz, CDCl_3_): *δ* (ppm) 121.2, 118.8, 116.0, 111.2 (6C, *C*F_2_ and *C*F_3_), 79.6 (1C, propargyl Cq), 74.6 (1C, propargyl CH), 68.5, 58.3 (2C, *C*H_2_), 28.2 (t, 1C, *C*H_2_), 20.9 (1C, *C*H_2_).

#### Compound 4

To the solution of **11** (143 mg, 0.1 mmol) in abs. dimethylformamide (3 mL) **10** (62 mg, 0.15 mmol) was added in abs. dimethylformamide (1 mL). The mixture was stirred under argon atmosphere for 15 min, then triethylamine (28 μL, 0.2 mmol) and Cu(I)I (0.04 mmol) were added. The reaction mixture was stirred overnight, then Na_2_S (10 mg) in water (1 mL) was added and stirred for 15 min. The solvent was evaporated and the product was purified by flash column chromatography (toluene/MeOH 6:4 containing 0.1 v/v% acetic acid) to yield **4** (98 mg, 53%) as a brownish white solid. *R*_f_ = 0.45 (toluene/MeOH 4:6 containing 1.0 v/v% acetic acid); HRMS (ESI): *m/z* calcd for C_78_H_64_Cl_2_F_13_N_10_O_24_Na + Na^+^: 1887.3054 [*M*-H + 2Na]^+^; found: 1887.3054. RP-HPLC: RT_1_ = 21.60 min, RT_2_ = 22.17 (purity: 95.7%).

#### Compound 12

Allyl alcohol (585 μL, 500 mg, 8.61 mmol) was dissolved in abs. methanol (5 mL), then perfluorooctyl iodide (2.765 mL, 10.33 mmol, 1.2 equiv.) and benzophenone (17 mg, 0.09 mmol) in abs. methanol (1 mL) were added. Argon gas was bubbled through the solution for 10 min and the reaction mixture was irradiated with UV light for 20 min. The solvent was evaporated and the product was purified by flash column chromatography (hexane/acetone 9:1) to yield **12** (4.5 g, 87%) as a white solid. *R*_f_ = 0.78 (hexane/acetone 98:2); ^1^H NMR (400 MHz, CDCl_3_):* δ* (ppm) 4.50–4.38 (m, 1H, C*H*I), 3.91–3.73 (m, 2H, C-1 C*H*_2_), 3.14–2.90 (m, 1H, C-3 C*H*_2_a), 2.89–2.66 (m, 1H, C-3 C*H*_2_b), 2.16 (t, 1H, O*H*); ^13^C NMR (100 MHz, CDCl_3_): *δ* (ppm) 120.5, 118.7, 117.9, 115.8, 110.9, 108,5 (8C, *C*F_2_ and *C*F_3_), 68.1 (1C, C-1*C*H_2_), 37.7 (t, 1C, C-3 *C*H_2_), 21.9 (1C, *C*HI).

#### Compound 13

Compound **12** (2.2 g, 3.64 mmol) was dissolved in abs. methanol (20 mL) and argon gas was bubbled through the solution for 10 min. 10% palladium on activated charcoal (440 mg) and NaHCO_3_ (0.77 g, 9.16 mmol, 2.5 equiv.) were added, and the reaction mixture was stirred overnight under H_2_ atmosphere, then filtered through Celite, washed with dichloromethane and the solvent was evaporated. The product was purified by flash column chromatography (hexane/acetone 9:1 and 8:2) to yield **13** (1.0 g, 57%) as a colourless syrup. *R*_f_ = 0.34 (hexane/acetone 8:2); ^1^H NMR (500 MHz, CDCl_3_):* δ* (ppm) 3.74 (t, 2H, *J* = 6.1 Hz, C*H*_2_OH), 2.30–2.12 (m, 2H, C*H*_2_), 1.92–1.81 (m, 2H, C*H*_2_), 1.66 (s, 1H, O*H*); ^13^C NMR (100 MHz, CDCl_3_): *δ* (ppm) 118.7, 116.2, 111.0 (8C, *C*F_2_ and *C*F_3_), 61.6 (1C, C-1 *C*H_2_), 27.8 (t, 1C, C-3 *C*H_2_), 23.5 (1C, C-2 *C*H_2_);

#### Compound 14

To the solution of **13** (500 mg, 1.0457 mmol) in dichloromethane (10 mL) and dist. water (5 mL) TEMPO (2,2,6,6-tetramethylpiperidine 1-oxyl) (30 mg, 0.188 mmol, 0.18 equiv.) and BAIB ((diacetoxyiodo)benzene) (1.01 g, 3.137 mmol, 3 equiv.) were added and the reaction mixture was stirred for 5 h. Then it was neutralized with 10% aqueous solution of Na_2_S_2_O_3_ (4.5 mL), diluted with dichloromethane (200 mL) and separated. The aqueous phase was extracted with dichloromethane (100 mL), the combined organic phase was dried over Na_2_SO_4_, the solution was filtered and the solvent was evaporated. The product was purified by flash column chromatography (hexane/acetone 85:15) to yield **14** (440 mg, 85%) as a white powder. *R*_f_ = 0.35 (hexane/acetone 7:3); ^1^H NMR (400 MHz, methanol-d_4_):* δ* (ppm) 2.47–2.30 (m, 4H, C*H*_2_); ^13^C NMR (100 MHz, methanol-d_4_): *δ* (ppm) 175.5 (1C, C=O), 119.7, 117.4, 114.6, 112.0, 109.3 (8C, *C*F_2_ and *C*F_3_), 27.5 (t, 1C, *C*H_2_), 26.0 (1C, *C*H_2_).

#### Compound 15

Compound **14** (389 mg, 0.79 mmol) was dissolved in the mixture of abs. dichloromethane (10 mL) and abs. THF (5 mL) and under argon atmosphere *N*-hydroxysuccinimide (100 mg, 0.869 mmol, 1.1 equiv.) was added and the mixture was cooled to 0 °C. After 10 min, EDC (*N*-(3-dimethylaminopropyl)-*N′*-ethylcarbodiimide hydrochloride) (166 mg, 0.869) was added and the reaction mixture was stirred overnight. The solvent was evaporated and the product was purified to yield **15** (436 mg, 94%) as a white powder. *R*_f_ = 0.38 (hexane/acetone 7:3); ^1^H NMR (400 MHz, CDCl_3_):* δ* (ppm) 3.04–2.93 (m, 2H, C*H*_2_), 2.86 (s, 4H, succinimide C*H*_2_), 2.67–2.48 (m, 2H, C*H*_2_); ^13^C NMR (100 MHz, CDCl_3_): *δ* (ppm) 168.8 (2C, succinimide *C*=O), 166.9 (1C, *C*=O), 26.4 (t, 1C, *C*H_2_), 25.7 (2C, succinimide *C*H_2_), 23.0 (1C, C*H*_2_). HRMS (ESI): *m/z* calcd for C_15_H_8_F_17_O_4_ + Na^+^: 612.0074 [*M* + Na]^+^; found: 612.0075.

#### Compound 5

Teicoplanin pseudoaglycone **16** (142 mg, 0.1 mmol) was dissolved in abs. dimethylformamide (3 mL) and triethylamine (21 μL, 0.15 mmol, 1.5 equiv.) was added. Under argon atmosphere **15** (77 mg, 0.13 mmol, 1.3 equiv.) was added, and the reaction mixture was stirred overnight. Then the solvent was evaporated and the product was purified by flash column chromatography to yield **5** (91 mg, 48%) as a dirty white solid. *R*_f_ = 0.19 (toluene/methanol 1:1 containing 1.0 v/v% acetic acid). HRMS (ESI): *m/z* calcd for C_77_H_60_Cl_2_F_17_N_8_O_24_Na + Na^+^: 1919.2616 [*M*-H + 2Na]^+^; found: 1919.2624. RP-HPLC: RT = 21.60 min, (purity: ~ 100%).

#### Compound 18

Benzyl gallate **17** (3.5 g, 13.4 mol) was dissolved in abs. dimethylformamide (30 mL) and K_2_CO_3_ (8.29 g, 60 mmol) and allyl bromide (3.02 mL, 48 mmol) were added. The reaction mixture was stirred overnight, then diluted with dichloromethane and stirred for 20 min. The mixture was washed with 4% aqueous solution of NaOH (2 × 50 mL) and saturated aqueous NaCl solution (3 × 50 mL), the organic phase was dried with Na_2_SO_4_, filtered and the solvent was evaporated. The product was purified by column chromatography (hexane/EtOAc 98:2) to yield **18** (3.0 g, 60%) as a yellowish syrup. *R*_f_ = 0.53 (hexane/EtOAc 8:2); ^1^H NMR (400 MHz, CDCl_3_): *δ* (ppm) 7.45–7.30 (m, 7H, aromatic C*H*), 6.14–5.99 (m, 3H, allyl C*H*), 5.44 (q, 1H, *J* = 1.6 Hz, allyl = C*H*_2_), 5.40 (q, 1H, *J* = 1.6 Hz, allyl = C*H*_2_), 5.36–5.30 (m, 3H, benzyl C*H*_2_, allyl = C*H*_2_), 5.29 (q, 1H, *J* = 1.5 Hz, allyl = C*H*_2_), 5.26 (q, 1H, *J* = 1.5 Hz, allyl = C*H*_2_), 5.18 (ddt, 1H, *J* = 10.3, 2.0, 1.1 Hz, allyl = C*H*_2_), 4.63 (t, 2H, *J* = 1.3 Hz, allyl C*H*_2_), 4.61 (t, 2H, *J* = 1.5 Hz, allyl C*H*_2_), 4.59 (t, 2H, *J* = 1.6 Hz, allyl C*H*_2_); ^13^C NMR (100 MHz, CDCl_3_): *δ* (ppm) 166.1 (1C, *C*=O), 152.4 (2C, quat. *C*), 142.1 (1C, quat. *C*), 136.2 (1C, quat. *C*), 134.3, 133.1 (3C, allyl *C*H), 128.7, 128.3, 128.2 (5C, aromatic *C*H), 125.1 (1C, quat. *C*), 118.0, 117.8 (3C, allyl *C*H_2_), 109.0 (2C, aromatic *C*H), 74.1, 70.1, 66.8 (4C, *C*H_2_). HRMS (ESI): *m/z* calcd for C_23_H_24_O_5_ + Na^+^: 403.1516 [*M* + Na]^+^; found: 403.1510.

#### Compound 19

To the solution of **18** (1 g, 3 mmol) in abs. methanol (15 mL) cysteamine hydrochloride (1.136 g, (10 mmol) and 2,2-dimethoxy-2-phenylacetophenone (DPAP, 77 mg, 0.3 mmol) were added. The reaction mixture was cooled to 0 °C and irradiated with UV light for 15 min 4 times, and DPAP (77 mg, 0.3 mmol) was added before each irradiation cycle. The solvent was evaporated, the residue was dissolved in distilled water (60 mL), saturated aqueous K_2_CO_3_ solution (100 mL) was added and it was extracted with dichloromethane (3 × 100 mL). The combined organic phase was dried over Na_2_SO_4_, filtered, and the solvent was evaporated in vacuum. The product was purified by by column chromatography (CH_2_Cl_2_/MeOH6:4 containing 0,01% Et_3_N) to yield **19** (1.1 g, 69%) as a yellow liquid. *R*_f_ = 0.10 (CH_2_Cl_2_/MeOH/Et_3_N 5:5:0.1); ^1^H NMR (500 MHz, CDCl_3_): *δ* (ppm) 7.46–7.27 (m, 7H, aromatic C*H*), 5.33 (s, 2H, benzyl C*H*_2_), 4.10 (t, 6H, *J* = 6.0 Hz, OC*H*_2_), 2.84–2.69 (m, 12H, C*H*_2_), 2.63 (t, 6H, *J* = 6.6 Hz, C*H*_2_), 2.06 (quin, 4H, *J* = 6.4 Hz, C*H*_2_), 1.97 (quin, 2H, J = 6.3 Hz, C*H*_2_). ^13^C NMR (100 MHz, CDCl_3_): *δ* (ppm) 167.4 (1C, *C*=O), 153.9 (2C, quat. *C*), 143.3 (1C, quat. *C*), 137.6 (1C, quat. *C*), 129.7, 129.3, 129.3 (5C, aromatic *C*H), 126.5 (1C, quat. *C*), 109.2 (2C, aromatic *C*H), 72.9, 68.6, 67.9 (4C, O*C*H_2_), 41.7, 41.7 (3C, C*H*_2_), 35.6 (3C, C*H*_2_), 31.6 (1C, C*H*_2_), 30.6 (2C, C*H*_2_), 29.1, 29.0 (3C, C*H*_2_). HRMS (MALDI): *m/z* calcd for C_29_H_45_N_3_O_5_S_3_ + Na^+^: 634.2414 [*M* + Na]^+^; found: 634.2402.

#### Compound 21

To the solution of **19** (1.0 g, 1.6 mmol) in abs. dimethylformamide (20 mL) triethylamine (2.7 mL, 0.02 mol) and **20** (1.64 g, 5.28 mmol) were added, and the reaction mixture was stirred overnight. The solvent was evaporated and the product was purified by flash column chromatography (hexane/acetone 8:2) to yield **21** (700 mg, 33%) as a yellowish syrup. *R*_f_ = 0.40 (hexane/EtOAc 7:3); ^1^H NMR (400 MHz, methanol-*d*_4_ + CDCl_3_): *δ* (ppm) 7.48–7.30 (m, 7H, aromatic C*H*), 5.36 (s, 2H, benzyl C*H*_2_), 4.21–4.12 (m, 6H, OC*H*_2_), 3.59 (t, 6H, *J* = 6.6 Hz, C*H*_2_), 2.88–2.72 (m, 12H, C*H*_2_), 2.19–2.08 (m, 4H, C*H*_2_), 2.07–1.97 (m, 2H, C*H*_2_), 1.55–1.44 (m, 54H, C*H*_3_). HRMS (MALDI): (cleavage of the BOC protecting groups occurred) *m/z* calcd for C_32_H_51_N_9_O_5_S_3_ + Na^+^: 760.3067 [*M* + Na]^+^; found: 760.3064.

#### Compound 22

Compound **21** (500 mg, 0.373 mmol) was dissolved in the mixture of dioxane (18 mL) and water (2 mL) and the mixture was cooled to 0 °C. 0.5 M NaOH solution (3.7 mL, 1.876 mmol, 5 equiv.) was added and the reaction mixture was stirred overnight at room temperature. Serdolit red H^+^ ion exchange resin was added and stirred untill neutralization. Then the resin was filtered out and the solvent was evaporated in vacuum and the product was purified by column chromatography (hexane/acetone 7:3) to yield **22** (317 mg, 68%) as a white solid. *R*_f_ = 0.42 (hexane/acetone 6:4); ^1^H NMR (400 MHz, CDCl_3_):* δ* (ppm) 8.71–8.60 (m, 3H, N*H*), 7.35 (s, 2H, aromatic C*H*), 4.21–4.07 (m, 6H, OC*H*_2_), 3.71–3.59 (m, 6H, NHC*H*_2_), 2.86–2.68 (m, 12H, SC*H*_2_), 2.13 (quin, 4H, *J* = 6.5 Hz, C*H*_2_), 2.01 (quin, 2H, J = 6.5 Hz, C*H*_2_). ^13^C NMR (100 MHz, CDCl_3_): *δ* (ppm) 170.6, 163.5, 163.4, 156.1, 153.1, 152.4, 142.2, 125.0, 83.2, 79.3 (20C, *C*=O, quat. *C*), 108.6 (2C, aromatic *C*H), 71.7, 67.3 (3C, O*C*H_2_), 40.0 (3C, N*C*H_2_), 31.1 (3C, S*C*H_2_), 30.3 (1C, *C*H_2_), 29.1 (2C, *C*H_2_), 28.3, 28.1 (18C, *C*H_3_), 28.2 (3C, S*C*H_2_). HRMS (MALDI): (cleavage of the BOC protecting groups occurred) *m/z* calcd for C_25_H_45_N_9_O_5_S_3_ + Na^+^: 670.2598 [*M* + Na]^+^; found: 670.2581.

#### Compound 23

To the solution of **22** (335 mg, 0.268 mmol) in abs. dichloromethane (15 mL) *N*-hydroxysuccinimide (34 mg, 0.295 mmol, 1.1 equiv.) was added and the mixture was cooled in ice bath to 0 °C. After addition of EDC (52 mg, 0.27 mmol) the reaction mixture was stirred overnight at room temperature. The solvent was evaporated and the producet was purified by flash column chromatography (hexane/acetone 8:2) to yield **23** (237 mg, 66%) as a white solid. *R*_f_ = 0.34 (hexane/acetone 7:3); ^1^H NMR (400 MHz, CDCl_3_):* δ* (ppm) 11.48 (s, 3H, N*H*), 8.66–8.59 (m, 3H, N*H*), 7.36 (s, 2H, aromatic C*H*), 4.19–4.11 (m, 6H, OC*H*_2_), 3.64 (q, 6H, *J* = 6.3 Hz, NHC*H*_2_), 2.91 (s, 4H, succinimide C*H*_2_), 2.84–2.71 (m, 12H, SC*H*_2_), 2.13 (quin., 4H, *J* = 6.6 Hz, C*H*_2_), 2.01 (quin., 2H, *J* = 6.5 Hz, C*H*_2_), 1.52–1.46 (m, 54H, C*H*_3_); ^13^C NMR (100 MHz, CDCl_3_): *δ* (ppm) 169.2, 163.5, 161.4, 156.1, 153.1, 152.8, 143.7, 119.6, 83.1, 83.1, 79.2 (22C, *C*=O, quat. *C*), 109.1 (2C, aromatic *C*H), 71.8, 67.5 (3C, O*C*H_2_), 40.0, 40.0 (3C, NH*C*H_2_), 31.2, 31.1 (3C, S*C*H_2_), 30.2, 29.0 (3C, *C*H_2_), 28.3 (9C, *C*H_3_), 28.2 (3C, S*C*H_2_), 28.1 (9C, *C*H_3_), 25.7 (2C, succinimide *C*H_2_). HRMS (ESI): *m/z* calcd for C_59_H_96_N_10_O_19_S_3_ + Na^+^: 1367.5908 [*M* + Na]^+^; found: 1367.5905.

#### Compound 7

Teicoplanin pseudoaglycone (175 mg, 0.125 mmol) was dissolved in abs. dimethylformamide (3 mL), then triethylamine (27 μl, 0.188 mmol, 1.5 equiv.) and **23** (220 mg, 1.163 mmol, 1.3 equiv) were added and the reaction mixture was stirred overnight. Then the solvent was evaporated and the product was purified by flash column chromatography (toluene/methanol 7:3 and 0.1% acetic acid) to yield a brownish white powder (190.5 mg, 58%) of the *tert*-butoxycarbonyl protected product. For the deprotection of the guanidino groups, the product of the previous reaction (180 mg, 0.0684 mmol) was dissolved in trifluoroacetic acid (2 mL) and anisole (15 mg, 0.137 mmol, 2 equiv.) was added. After 2 h stirring, the product was precipitated with abs. diethyl ether, filtered and dried to yield **7** (151 mg, 93%) as a dirty white solid. *R*_f_ = 0.38 (toluene/methanol 55:45 containing 1.0 V/V% aceic acid) for the first step, and *R*_f_ = 0.28 (acetonitrile/water 9:1) for **7**. HRMS (ESI): *m/z* calcd for C_91_H_101_Cl_2_N_17_O_27_S_3_ + 2H^+^: 1015.7869 [*M* + 2H]^2+^; found: 1015.7917. RP-HPLC: RT = 21.60 min (purity: ~ 100%).

#### Compound 24

To the solution of **21** (300 mg, 0.244 mmol) in dimethylformamide (4 mL) trifluoroacetic acid (3 mL) was added. After 4 h stirring, the solvent was evaporated and toluene (4 mL) was added and evaporated in vacuum. The residue was dissolved in dist. water (6 mL) and Amberlite IRA 400 Cl^-^ ion exchange resin was added and the mixture was stirred for 4 h, then it was filtered and the solvent was evaporated to yield **24** (190 mg, 100%) as a yellow syrup. *R*_f_ = 0.40 (CH_2_Cl_2_/MeOH 8:2); ^1^H NMR (400 MHz, methanol-*d*_4_):* δ* (ppm) 8.34 (d, 6H, *J* = 2.7 Hz, N*H*_2_), 7.48–7.31 (m, 7H, aromatic C*H*), 6.85 (t, 2H, *J* = 2.7 Hz, N*H*), 5.35 (s, 2H, benzyl C*H*_2_), 4.14 (t, 6H, *J* = 5.9 Hz, OC*H*_2_), 3.48–3.40 (m, 6H, NC*H*_2_), 2.88–2.74 (m, 12H, SC*H*_2_), 2.10 (quin., 4H, *J* = 6.5 Hz, C*H*_2_), 1.99 (quin., 2H, *J* = 6.4 Hz, C*H*_2_). ^13^C NMR (100 MHz, methanol-*d*_4_): *δ* (ppm) 167.1, 158.4, 158.4, 153.6, 142.9, 137.6, 126.2 (9C, *C*=O and quat. *C*), 129.6, 129.2, 129.1 (5C, aromatic benzyl *C*H), 109.0 (2C, acomatic *C*H), 72.8, 68.4, 67.8 (4C, benzyl *C*H_2_, O*C*H_2_), 42.2, 42.1 (3C, N*C*H_2_), 31.7, 31.3, 30.3, 29.2, 29.2 (9C, S*C*H_2_, *C*H_2_). HRMS (ESI): *m/z* calcd for C_32_H_51_N_9_O_5_S_3_ + 2H^+^: 369.6660 [*M* + 2H]^2+^; found: 369.6655.

### Anti-coronavirus activity and cytotoxicity in CPE reduction assays

We used the following viruses and cell lines: SARS-CoV-2 (strain hCoV-19/Czech Republic/NRL_6632_2/2020, clade G, lineage B.1); HCoV-229E virus (VR-740 from ATCC); and VeroE6, Calu-3 and HEL cells (all three from ATCC). For the SARS-CoV-2 assay, two-fold serial dilutions of the compounds were prepared from 100 to 0.78 µM, then added in triplicate in a 384-well plate containing 5000 VeroE6 cells per well or a 96-well plate containing 40,000 Calu-3 cells per well. The medium consisted of DMEM supplemented with 2% FBS, penicillin and streptomycin. After 1 h incubation, SARS-CoV-2 was added at a multiplicity of infection of 0.04 IU/mL and 0.05 IU/mL, respectively. After three days incubation at 37 °C, the virus-induced cytopathic effect was quantified by formazan-based cell viability assay. After adding the XTT solution (Sigma-Aldrich) and 4 h incubation, the absorbance at 450 nm was measured using an EnVision plate reader (Perkin Elmer). After plotting the percentage cell viability versus log_10_ concentrations, the compound concentrations required to reduce viral cytopathic effect by 50% (EC_50_) were calculated by nonlinear regression, using GraphPad Prism software.

A similar assay was used for HCoV-229E^32^. After growing HEL cells in 96-well plates until confluency, the cells were exposed to serial dilutions of the compounds. Immediately afterwards, HCoV-229E was added at an MOI of 100 CCID_50_ per well. After five days incubation at 35 °C, the MTS reagent was added (CellTiter 96 AQ_ueous_ MTS Reagent from Promega). Twenty-four hours later, the absorbance at 490 nm was measured in a plate reader. The EC_50_ values were calculated as explained above.

To assess compound cytotoxicity, mock-infected cultures of the VeroE6, Calu-3 and HEL cells were exposed to the compound dilutions as above. For the VeroE6 and Calu-3 cells, the XTT assay was conducted after three days incubation, while the HEL cells were submitted to the MTS procedure after five days incubation. The compound concentrations causing 50% reduction in cell viability (CC_50_) were calculated by Graphpad, in the same way as explained for the EC_50_ calculations.

### Inhibition of SARS-CoV-2 spike-mediated pseudovirus entry

Firefly luciferase (fLuc)-expressing MLV pseudovirus carrying the S protein of SARS-CoV-2 (D614G variant of an early 2020 isolate with NCBI accession number YP_009724390.1) was produced as reported^13^. Briefly, HEK293T cells in 6-well plates were transfected with three plasmids encoding MLV gag-pol and fLuc reporter (kind gift from S. Pöhlmann, Germany) plus the S protein. After three days incubation at 33 °C, the pseudovirus-containing supernatants were harvested and frozen. For the entry inhibition assay, Vero cells or A549-AT cells (i.e. A549 cells expressing ACE2 and TMPRSS2; code a549d-cov2r from Invivogen) were seeded at 7500 cells per well in white half-area 96-well plates. One day later, the cells were pre-incubated with serial compound dilutions (in serum-free medium) for 20 min at 37 °C, after which the pseudovirus was added and spinoculated by centrifugating the plates at 453 × *g* for 45 min at 37 °C. After placing the plates for two more hours in the incubator, the supernatants were removed and replaced by medium with 10% FCS. After 3 days incubation at 33 °C, fLuc activity was monitored using a luciferase assay system kit and GloMax® Navigator Microplate Luminometer (both from Promega). The EC_50_ values of the compounds were calculated with Graphpad software, as explained in Section “Anti-coronavirus activity and cytotoxicity in CPE reduction assays”.

### Enzyme inhibition assays

Cathepsin L inhibition was assessed as reported^9^. Briefly, human recombinant cathepsin L was expressed in *Escherichia coli*^33^. Enzymatic activity was determined in 100 mM acetate buffer (pH 5.5) containing 0.1% PEG 8000, 5 mM cysteine and 1.5 mM EDTA. The enzyme was first activated in assay buffer for 5 min at 37 °C, after which it was added to the wells of a black microplate containing the Z-Phe-Arg-AMC substrate (Bachem) and compounds. Formation of the fluorescent cleavage product was monitored for 50 min at 37 °C, using a Tecan Infinit M1000 spectrofluorimeter set at 460 nm emission and 380 nm excitation wavelength. All assay mixtures contained 5% (v/v) DMSO and 0.01% Triton X-100 to prevent false-positive inhibition due to formation of compound aggregates^34^. All measurements were performed in triplicate and repeated twice. The relative inhibition was calculated using the equation: *Relative inhibition (%)* = *100(1 − v*_*i*_*/v*_*o*_*)*, where *v*_*i*_ and *v*_*o*_ designate the reaction velocities in the presence and absence of inhibitor, respectively. IC_50_ values were determined from nine data points at different inhibitor concentrations.

Inhibition of the SARS-CoV-2 main protease was conducted as recently described^9^. Briefly, 3CL^Pro^ (GenBank sequence MN908947.3 and codon-optimized by Thermo Fisher Scientific) was expressed in Rosetta2 cells (Novagen). We used a FRET substrate (Hilyte™ Fluor-488-ESATLQSGLRKAK-(QXL®-520)-NH2, Cat. AS-65599 from Anaspec)^35^ and a pH 7.5 buffer containing 0.1 M NaH_2_PO_4_, 1 mM EDTA and 100 mM NaCl. Fluorescence was monitored in black 384-well plates using a Victor^2^ 1420 multilabel counter (Perkin Elmer) set at 485 nm emission and 520 nm excitation wavelength. The compounds were tested at 200 µM concentration, with each sample assessed in duplicate.

To assess inhibition of the binding interaction between the SARS-CoV-2 spike receptor-binding domain (S-RBD) and human ACE2 receptor (hACE2), we used the RayBio® COVID-19 Spike-ACE2 binding assay kit (RayBiotech). hACE2-coated wells were incubated with 100 μM of the compounds for 16 h at 4 °C. After adding the S-RBD protein, the manufacturer’s protocol was followed. Briefly, after removing unbound S-RBD by washing, the HRP-conjugated IgG was allowed to interact with the bound S-RBD protein, then exposed to TMB (3,3’,5,5’-tetramethylbenzidine) substrate. The HRP-TMB reaction was halted with Stop Solution, and the absorbance at 450 nm was measured in a SpectraMax® iD5 reader (Molecular Devices). Datapoints were acquired in two independent replicates and percent inhibition values were calculated as *(A*_*0 *_*− A)/A*_*0*_ × *100*, where *A* is the absorbance in the compound sample and *A*_*0*_ is the absorbance for the no compound control.

### Antibacterial evaluations

We used the broth microdilution method according to EUCAST ISO standard 20,776–1 and the MIC results for the known glycopeptide antibiotics were read according to EUCAST clinical breakpoints (EUCAST, 2019). The collection of bacterial strains was obtained from the Institute of Microbiology (CAS). Vancomycin, teicoplanin and oritavancin were purchased from Sigma-Aldrich, and dalbavancin from MedChemExpress. Vancomycin-BODIPY conjugate was purchased from ThermoFisher Scientific. Fluorescently labelled teicoplanin was synthesized as reported earlier^28^.

The fluorescent binding assay was done as described earlier^28^. Briefly, *S. aureus* ATCC29213 was grown in MH medium to A_630nm_ = 0.4. The cells were pelleted at 5000 rpm for 10 min, washed twice with 50 mM Tris–HCl pH 7.4 and resuspended in this buffer to A_630nm_ = 1. After adding FL-VAN (final concentration: 1 mg/mL) or FL-TEI (10 mg/mL), the cells were incubated for 10 min at room temperature. Next, they were pelleted and washed twice with Tris–HCl buffer as above, then resuspended in 1 mL buffer and divided in six 100 µL aliquots. The non-fluorescent antibiotics or *N*-acetyl-d-Ala-d-Ala dipeptide (Sigma-Aldrich) were added at different concentrations (1, 10, 20, 40, 100, 500, 1000 µg/mL) and the bacteria were incubated for 10 min at room temperature. After cell centrifugation, fluorescence released in the supernatant was measured by Tecan Infinite 200Pro. The reader was set at 485 nm excitation and 528 nm emission wavelength for FL-VAN, and 530 nm excitation and 590 nm emission wavelength for FL-TEI. Each experiment was performed twice, with three replicates.

## Supplementary Information

Supplementary Information.

## Data Availability

The datasets used and/or analysed during the current study available from the corresponding author (A.B.) on reasonable request.
