# Supplementary material for: Semisynthetic teicoplanin derivatives with dual antimicrobial activity against SARS-CoV-2 and multiresistant bacteria
Source: Sci Rep. 2022 Sep 26;12:16001. doi: 10.1038/s41598-022-20182-y (PMC9511441; doi:10.1038/s41598-022-20182-y)

**Supplementary information**

**Semisynthetic teicoplanin derivatives with dual antimicrobial activity against SARS-CoV-2 and multiresistant bacteria**

Ilona Bereczki, Vladimir Vimberg, Eszter Lőrincz, Henrietta Papp, Lajos Nagy, Sándor Kéki, Gyula Batta, Ana Mitrović, Janko Kos, Áron Zsigmond, István Hajdú, Zsolt Lőrincz, Dávid Bajusz, László Petri, Jan Hodek, Ferenc Jakab, György M. Keserű, Jan Weber, Lieve Naesens, Pál Herczegh, Anikó Borbás

Table of content

[NMR data of **4** S3](#_Toc112679934)

[NMR data of **5** S4](#_Toc112679935)

[NMR data of **7** S5](#_Toc112679936)

[HPLC-MS of compound **4** S7](#_Toc112679937)

[HPLC-MS of compound **5** S9](#_Toc112679938)

[HPLC-MS of compound **7** S10](#_Toc112679939)

[Anti-SARS-CoV-2 activity determination in VERO E6 cells S11](#_Toc112679940)

[Cytotoxicity in VERO E6 cells S12](#_Toc112679941)

[Anti-SARS-CoV-2 activity determination in CaLu3 cells S13](#_Toc112679942)

[Cytotoxicity in CaLu3 cells (XTT assay- CC_50_ measurement) S14](#_Toc112679943)

[Cathepsin L inhibitory properties of compounds S15](#_Toc112679944)

[Antibacterial evaluation S16](#_Toc112679945)

[NMR spectra S19](#_Toc112679946)

[NMR spectra of **4** S19](#_Toc112679947)

[NMR spectra of **5** S20](#_Toc112679948)

[NMR spectra of **7** S22](#_Toc112679949)

[NMR spectra of **8** S23](#_Toc112679950)

[NMR spectra of **9** S25](#_Toc112679951)

[NMR spectra of **10** S26](#_Toc112679952)

[NMR spectra of **12** S27](#_Toc112679953)

[NMR spectra of **13** S28](#_Toc112679954)

[NMR spectra of **14** S29](#_Toc112679955)

[NMR spectra of **15** S30](#_Toc112679956)

[NMR spectra of **18** S31](#_Toc112679957)

[NMR spectra of **19** S32](#_Toc112679958)

[NMR spectrum of **21** S33](#_Toc112679959)

[NMR Spectra of **22** S34](#_Toc112679960)

[NMR spectra of **23** S36](#_Toc112679961)

[NMR spectra of **24** S38](#_Toc112679962)

# NMR data of **4**

Figure S1. Structure and numbering of compound **4**

Table S1. NMR data of compound **4**

| Annotation | ^1^H [ppm] | ^13^C [ppm] |
| --- | --- | --- |
| 1b | 6.871 | 119.24 |
| 1e | 6.962 | 120.46 |
| 1f | 6.714 | 126.03 |
| 2b | 7.151 | 131.02 |
| 2e | 7.025 | 124.95 |
| 2f | 7.691 | 131.72 |
| 3b | 6.327 | 110.22 |
| 3d | 6.598 | 104.16 |
| 3f | 6.294 | 102.14 |
| 4b | 5.569 | 108.38 |
| 4f | 5.108 | 104.98 |
| 5 | 7.723 | 124.63 |
| 5b | 7.099 | 136.73 |
| 5e | 6.632 | 116.97 |
| 5f | 6.648 | 125.78 |
| 6 | 4.451 | 63.59 |
| 6b | 7.834 | 129.02 |
| 6e | 7.252 | 123.66 |
| 6f | 7.253 | 128.37 |
| 7d | 6.328 | 104.80 |
| 7f | 6.510 | 108.47 |
| 8 | 3.491 | 68.30 |
| DMSO | 2.509 | 40.32 |
| G1 | 4.399 | 99.63 |
| G2 | 3.543 | 56.27 |
| G3 | 3.257 | 70.26 |
| G4 | 3.407 | 73.80 |
| G5 | 3.100 | 77.34 |
| G6 | 3.633 | 60.47 |
| x1 | 7.062 | 64.96 |
| x2 | 4.984 | 55.74 |
| x3 | 5.372 | 59.19 |
| x4 | 5.591 | 55.06 |
| x5 | 4.373 | 54.00 |
| x6 | 4.161 | 61.35 |
| z2a | 3.249 | 37.59 |
| z2b | 2.834 | 37.59 |
| z6 | 5.416 | 76.39 |

# NMR data of **5**

Figure S2. Structure and numbering of compound **5**

Table S2. NMR data of compound **5**

| Annotation | ^1^H [ppm] | ^13^C [ppm] |
| --- | --- | --- |
| *DMSO | 2.516 | 40.28 |
| 1b | 6.766 | 119.49 |
| 1e | 6.926 | 118.83 |
| 1f | 6.931 | 125.55 |
| 2b | 7.186 | 131.17 |
| 2e | 7.145 | 125.17 |
| 2f | 7.607 | 131.03 |
| 3 | 1.254 | 29.36 |
| 3b | 6.329 | 110.34 |
| 3d | 6.487 | 103.73 |
| 3f | 6.310 | 102.23 |
| 4 | 2.514 | 26.23 |
| 4b | 5.542 | 108.07 |
| 4f | 5.113 | 105.00 |
| 5b | 7.116 | 136.65 |
| 5e | 6.651 | 117.02 |
| 5f | 6.666 | 125.74 |
| 6b | 7.845 | 129.08 |
| 6e | 7.254 | 123.69 |
| 6f | 7.258 | 128.35 |
| 7d | 6.344 | 105.17 |
| 7f | 6.517 | 108.43 |
| G1 | 4.382 | 99.87 |
| G2 | 3.578 | 56.16 |
| G3 | 3.273 | 70.14 |
| G4 | 3.422 | 73.63 |
| G5 | 3.093 | 77.33 |
| G6 | 3.651 | 60.27 |
| x1 | 5.777 | 56.11 |
| x2 | 4.998 | 55.23 |
| x3 | 5.346 | 58.83 |
| x4 | 5.605 | 55.21 |
| x5 | 4.380 | 54.09 |
| x6 | 4.194 | 61.32 |
| x7 | 4.314 | 59.79 |
| z2a | 3.287 | 37.68 |
| z2b | 2.775 | 37.68 |
| z6 | 5.395 | 76.64 |

# NMR data of **7**

Figure S3. Structure and numbering of compound **7**

Table S3. NMR data of compound **7**

| Annotation | ^1^H [ppm] | ^13^C [ppm] |
| --- | --- | --- |
| 1" | 4.040 | 71.69 |
| 1', 1'" | 4.109 | 67.68 |
| 1b | 6.852 | 119.80 |
| 1e | 7.019 | 119.13 |
| 1f | 7.083 | 126.05 |
| 2" | 1.905 | 30.44 |
| 2', 2'" | 2.026 | 29.38 |
| 2b | 7.256 | 131.49 |
| 2e | 7.325 | 125.41 |
| 2f | 7.693 | 130.56 |
| 3', 3", 3'" | 2.751 | 28.11 |
| 3b | 6.386 | 110.61 |
| 3d | 6.385 | 105.39 |
| 3f, 7d | 6.429 | 103.23 |
| 4', 4", 4'" | 2.691 | 30.70 |
| 4,8 | 7.320 | 107.06 |
| 4b | 5.592 | 108.10 |
| 4f | 5.121 | 105.13 |
| 5', 5", 5'" | 3.360 | 41.09 |
| 5b | 7.100 | 136.19 |
| 5e | 6.706 | 117.16 |
| 5f | 6.735 | 126.34 |
| 6b | 7.882 | 129.13 |
| 6e | 7.267 | 123.83 |
| 6f | 7.283 | 128.60 |
| 7f | 6.269 | 106.36 |
| DMSO | 2.527 | 40.28 |
| G1 | 4.369 | 99.81 |
| G2 | 3.405 | 56.55 |
| G3 | 3.105 | 71.14 |
| G4 | 3.453 | 73.53 |
| G5 | 3.069 | 77.22 |
| G6a | 3.718 | 61.62 |
| G6b | 3.509 | 61.61 |
| x1 | 5.844 | 57.36 |
| x2 | 5.001 | 55.10 |
| x3 | 5.369 | 58.51 |
| x4 | 5.676 | 55.39 |
| x5 | 4.329 | 54.27 |
| x6 | 4.183 | 61.44 |
| x7 | 4.478 | 57.04 |
| z2a | 3.369 | 37.40 |
| z2b | 2.853 | 37.39 |
| z6 | 5.286 | 76.76 |

# HPLC-MS of compound **4**

Figure S4. HPLC chromatogram of compound **4**

Figure S5. MS spectrum of peak 1.

Figure S6. MS spectrum of peak 2.

Table S4. List of peaks of the HPLC-MS of compound **4**.

| # | RT [min] | Area | Area Frac. % |
| --- | --- | --- | --- |
| 1 | 21.60 | 384.7 | 95.7 |
| 2 | 22.17 | 38.8 |  |
| 3 | 23.50 | 18.8 | 4.3 |

# HPLC-MS of compound **5**

Figure S7. HPLC chromatogram of compound **5**

Figure S8. MS spectrum of peak 1.

# HPLC-MS of compound **7**

Figure S9. HPLC chromatogram of compound **7**

Figure S10. MS spectrum of peak 1.

# Anti-SARS-CoV-2 activity determination in VERO E6 cells


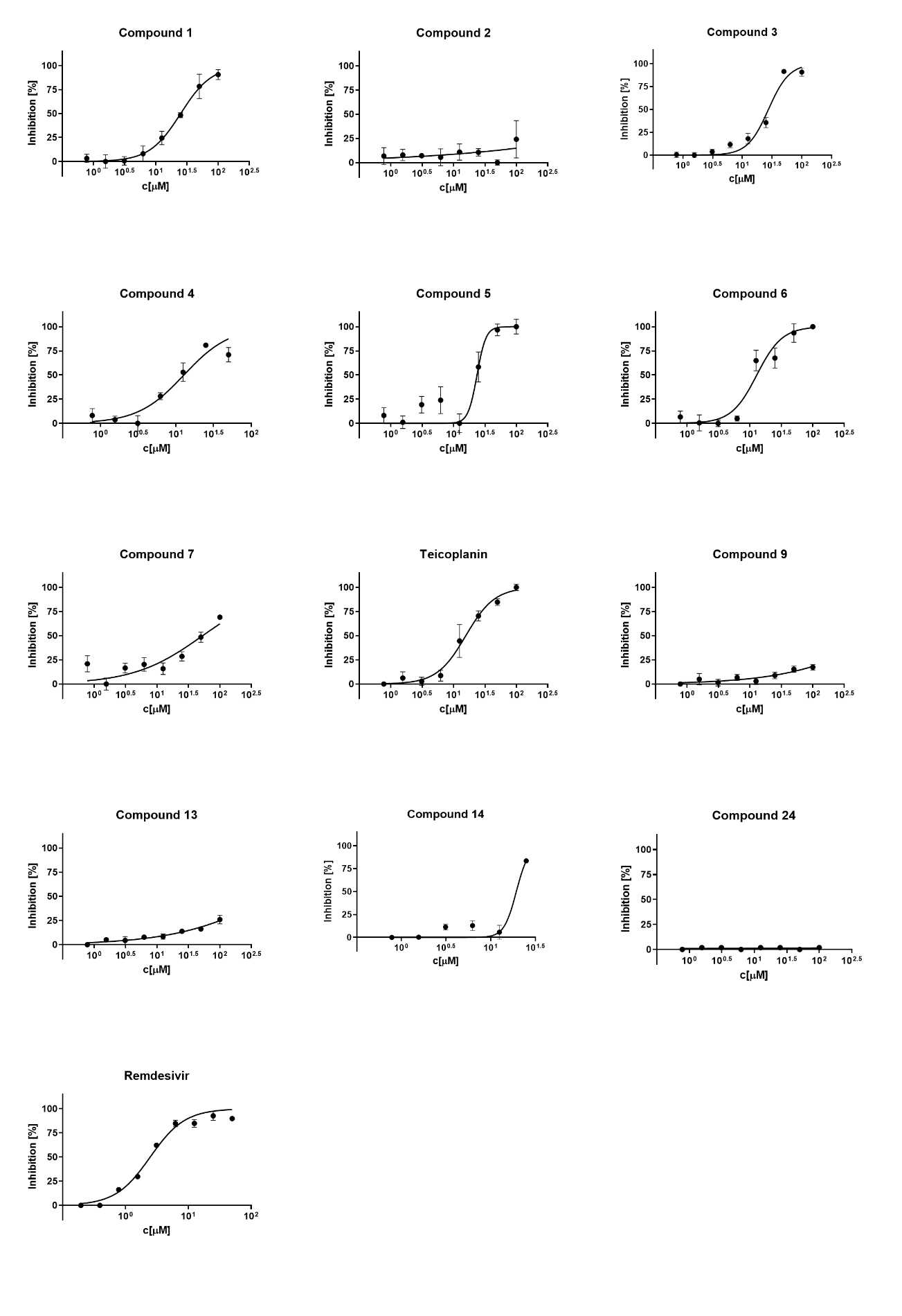


Figure S11. Inhibition of SARS-CoV-2-induced cytopathic effect in Vero E6 cells

# Cytotoxicity in VERO E6 cells


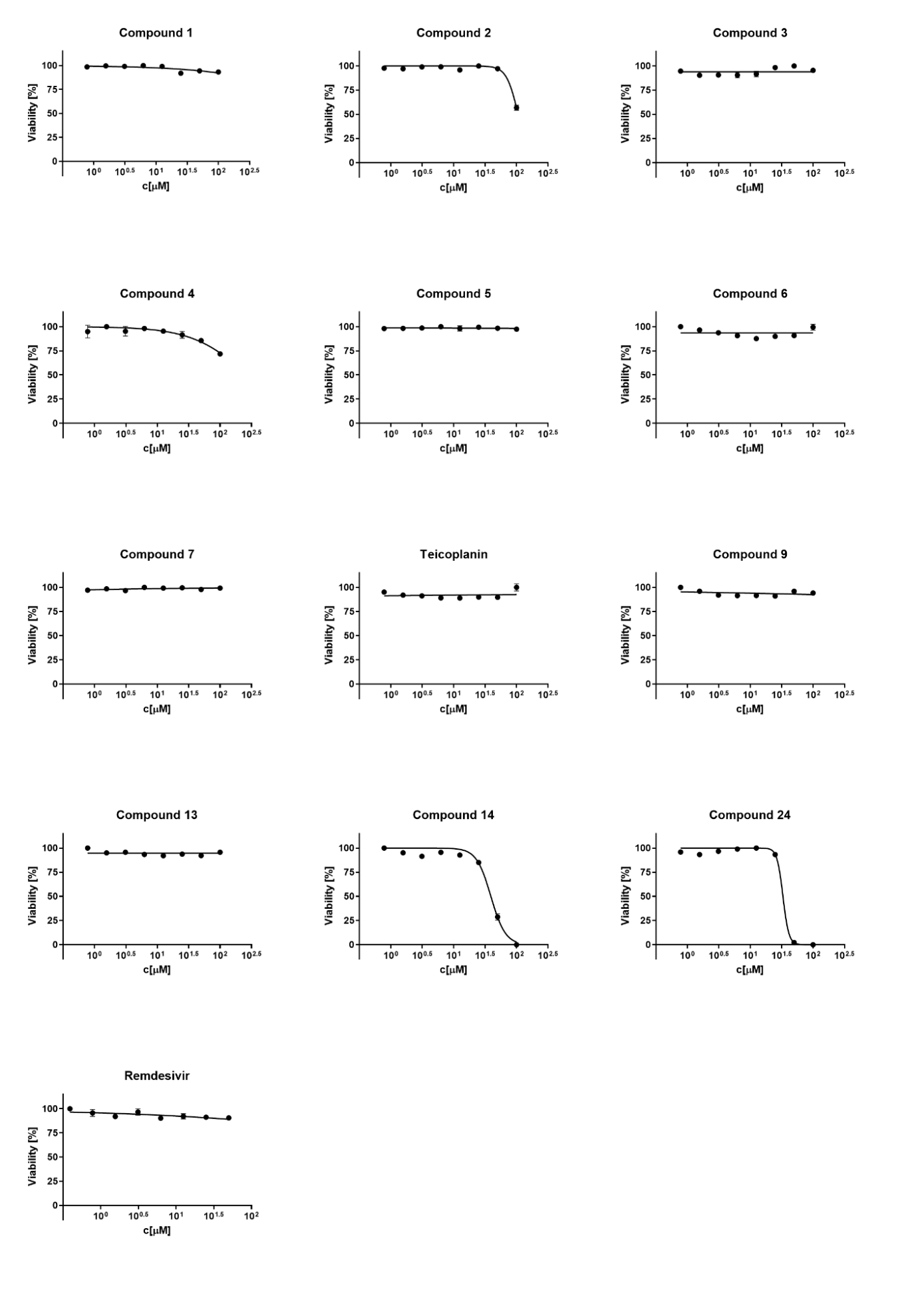


Figure S12. Cytotoxicity determination in Vero E6 cells

# Anti-SARS-CoV-2 activity determination in CaLu3 cells


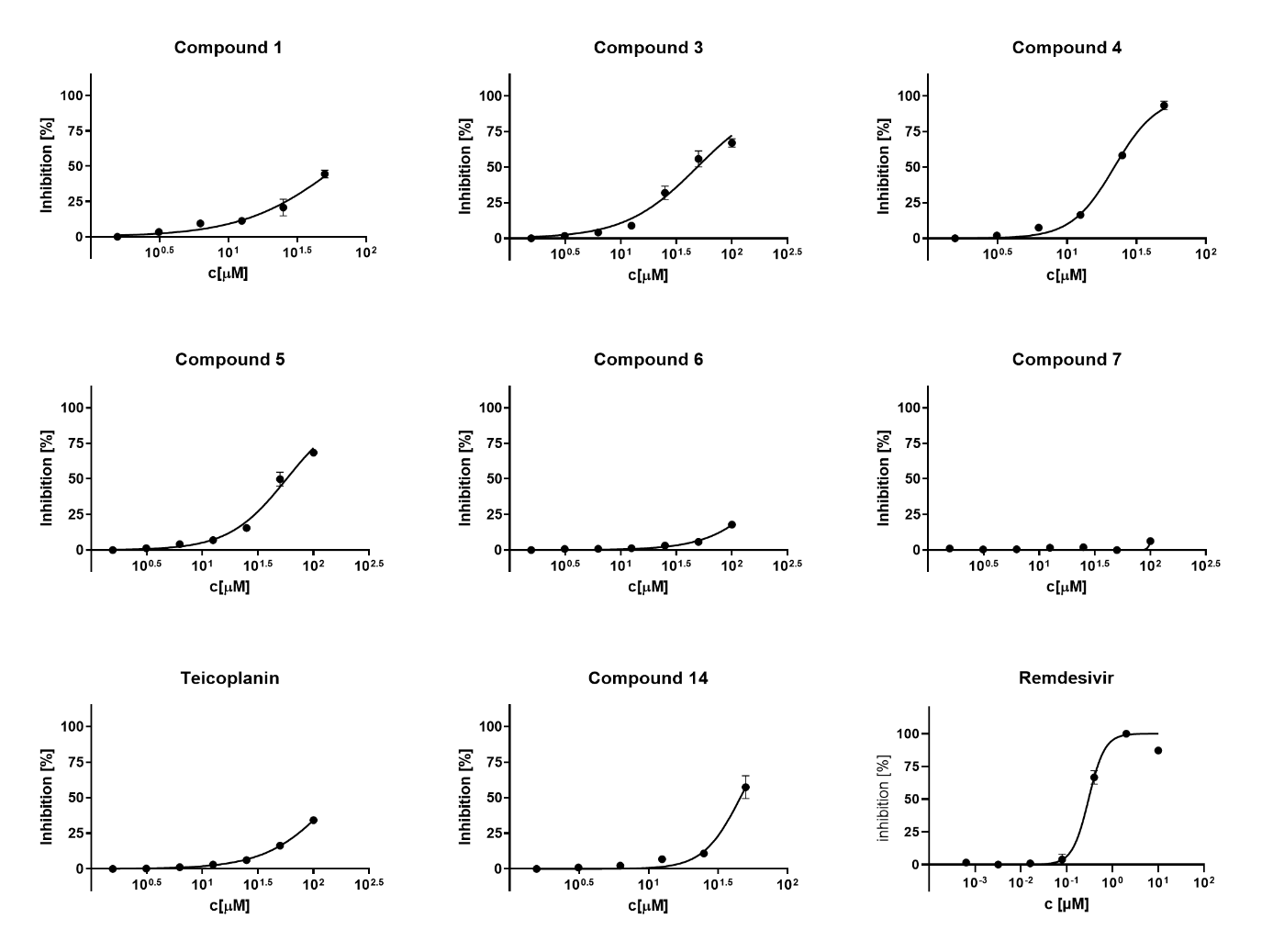


Figure S13. Inhibition of SARS-CoV-2-induced cytopathic effect in Calu-3 cells

# Cytotoxicity in CaLu3 cells (XTT assay- CC_50_ measurement)


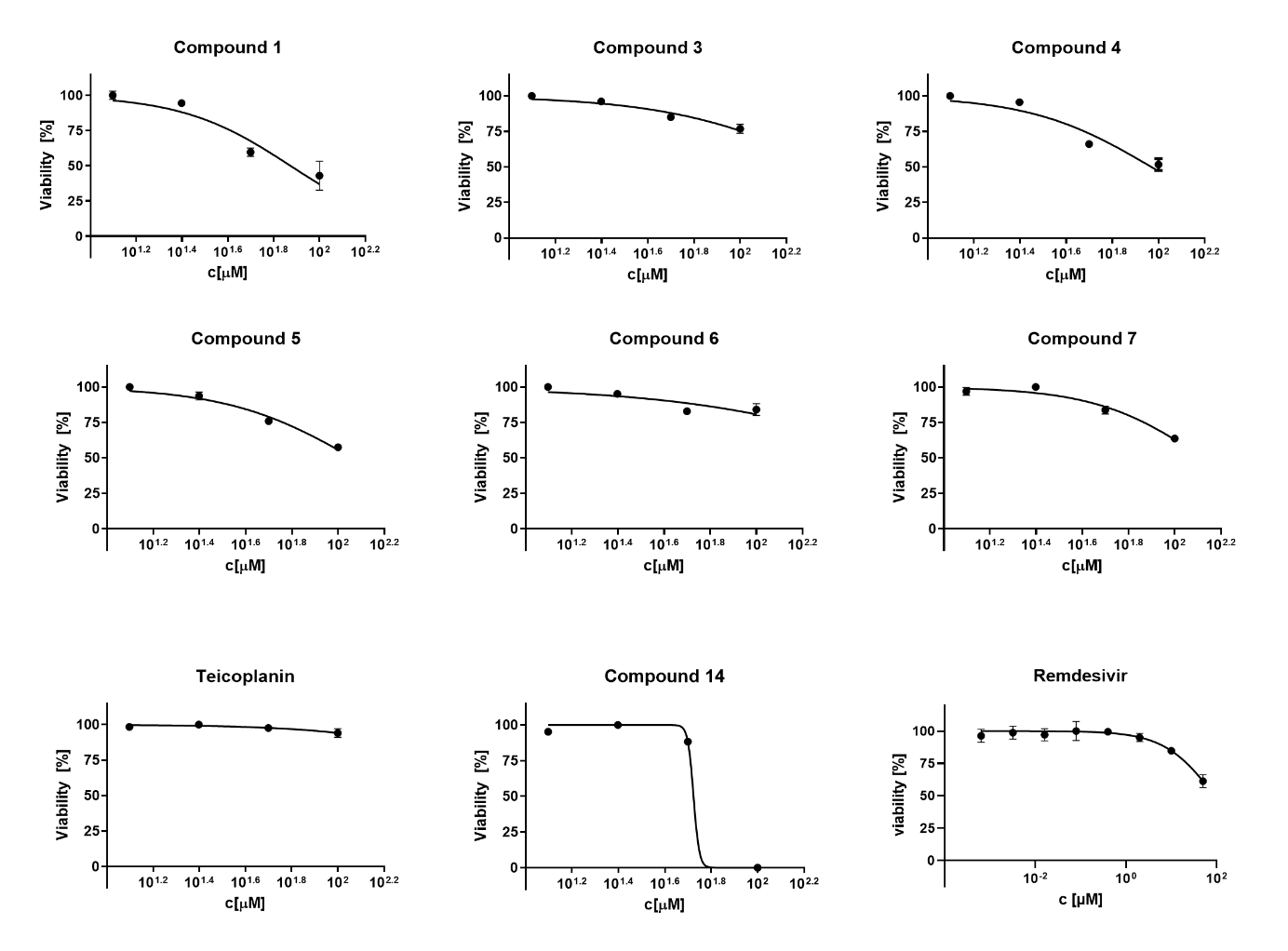
Figure S14. Cytotoxicity determination in Calu-3 cells

# Cathepsin L inhibitory properties of compounds


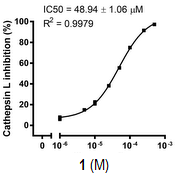

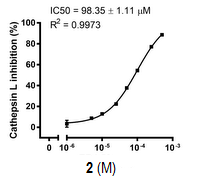

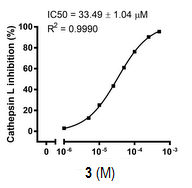


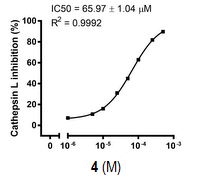

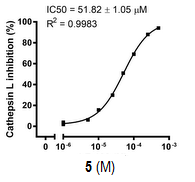

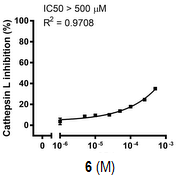


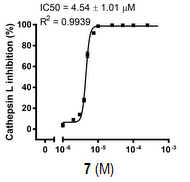

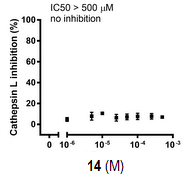

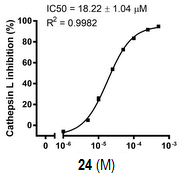


Figure S15. IC_50_ graphs of Cathepsin L inhibitory properties

# Antibacterial evaluation

Table S5. Activity of compounds **2-7** and glycopeptide antibiotic drugs (VAN, TEI, DALB, ORI) against clinically relevant Gram-positive bacteria

| **Bacterial species** | **Phenotype** | **MIC value (µg/ml) for compound^a^** | | | | | | | | | |
| --- | --- | --- | --- | --- | --- | --- | --- | --- | --- | --- | --- |
| ***S. aureus*** |  | **2** | **3** | **4** | **5** | **6** | **7** | **VAN** | **TEI** | **DALB** | **ORI** |
| ATCC29213 | MSSA | 0,0625 | 2 | 1 | 1 | 0,25 | 0,5 | 1 | 0,5 | 0,125 | 0,125 |
| Newman | MSSA | 0,5 | 4 | 2 | 2 | 0,25 | 1 | 0,5 | 0,25 | 0,125 | 0,0625 |
| USA300 | CA-MRSA | 0,25 | 2 | 2 | 4 | 0,125 | 1 | 0,25 | 0,125 | 0,0625 | 0,0625 |
| SA564 | MSSA | 0,25 | 2 | 2 | 2 | 0,125 | 1 | 0,5 | 0,5 | 0,125 | 0,0625 |
| N315 | MRSA | 0,25 | 2 | 2 | 2 | 0,125 | 0,25 | 0,5 | 1 | 0,125 | 0,125 |
| MSSA476 | MSSA | 0,25 | 2 | 2 | 2 | 0,125 | 2 | 0,5 | 0,25 | 0,125 | 0,0625 |
| Mu50 | MRSA | 0,25 | 2 | 2 | 2 | 0,125 | 1 | 4 | 4 | 0,125 | 0,25 |
| 214OL | MRSA | 0,25 | 2 | 2 | 4 | 0,125 | 1 | 1 | 2 | 0,125 | 0,125 |
| 1041OL | MRSA | 0,25 | 4 | 2 | 4 | 0,125 | 1 | 1 | 1 | 0,03125 | 0,125 |
| 73OL | MRSA | 0,25 | 2 | 2 | 2 | 0,125 | 1 | 1 | 0,5 | 0,125 | 0,125 |
| A6918 | MRSA | 0,5 | 2 | 1 | 2 | 0,125 | 1 | 1 | 1 | 0,125 | 0,125 |
| A6919 | MRSA | 0,25 | 2 | 1 | 2 | 0,125 | 1 | 0,25 | 0,5 | 0,0625 | 0,125 |
|  |  |  |  |  |  |  |  |  |  |  |  |
| ***E.faecalis*** | **Phenotype** | **2** | **3** | **4** | **5** | **6** | **7** | **VAN** | **TEI** | **DALB** | **ORI** |
| JH2-2 |  | 1 | >8 | 0,25 | 0,25 | 0,125 | 2 | 0,25 | 0,25 | 0,25 | 0,0625 |
| OG1RF |  | 1 | >8 | 0,25 | 0,25 | 0,125 | 2 | 0,25 | 0,25 | 0,25 | 0,0625 |
| V583 | VAN,TEI/VanA | >8 | >8 | >8 | >8 | 0,125 | >8 | >64 | >64 | 64 | 0,125 |
|  |  |  |  |  |  |  |  |  |  |  |  |
| ***E.faecium*** | **Phenotype** | **2** | **3** | **4** | **5** | **6** | **7** | **VAN** | **TEI** | **DALB** | **ORI** |
| TX0016 |  | 1 | >8 | 0,25 | 0,25 | 0,0625 | 2 | 0,25 | 0,5 | 0,25 | 0,0625 |
| E1132 | VAN,TEI,LNZ/VanA | >8 | >8 | >8 | >8 | 0,0625 | >8 | >64 | >64 | 32 | 0,125 |
| E1131 | VAN,TEI,LNZ/VanA | >8 | >8 | >8 | >8 | 0,0625 | >8 | >64 | >64 | 64 | 0,125 |
| E1114 | VAN,TEI,LNZ/VanA | >8 | >8 | >8 | >8 | 0,5 | >8 | >64 | >64 | 64 | 0,25 |
| E859 | VAN,TEI,LNZ/VanA | >8 | >8 | >8 | >8 | 0,125 | >8 | >64 | >64 | 32 | 0,0625 |
| E854 | VAN,TEI,LNZ/VanA | >8 | >8 | >8 | >8 | 0,125 | >8 | >64 | >64 | 32 | 0,0625 |
| E832 | VAN,TEI,LNZ/VanA | >8 | >8 | >8 | >8 | 0,125 | >8 | >64 | >64 | 64 | 0,125 |
| E355 | VAN,TEI,LNZ/VanA | >8 | >8 | >8 | >8 | 0,125 | >8 | >64 | >64 | 32 | 0,0625 |
| E335 | VAN,TEI,LNZ/VanA | >8 | >8 | >8 | >8 | 0,125 | >8 | >64 | >64 | 64 | 0,125 |
|  |  |  |  |  |  |  |  |  |  |  |  |
| ***S.pyogenes*** | **Phenotype** | **2** | **3** | **4** | **5** | **6** | **7** | **VAN** | **TEI** | **DALB** | **ORI** |
| H232 |  | 0,0625 | 0,125 | 0,0625 | 0,25 | 0,125 | 0,25 | 0,0625 | 0,03125 | 0,0625 | 0,125 |
| H550 |  | 0,0625 | 0,125 | 0,0625 | 0,25 | 0,125 | 0,25 | 0,0625 | 0,25 | 0,25 | 0,0625 |
| H578 |  | 0,0625 | 0,125 | 0,0625 | 0,25 | 0,125 | 0,25 | 0,0625 | 0,25 | 0,25 | 0,0625 |
|  |  |  |  |  |  |  |  |  |  |  |  |
| ***S.epidermidis*** | **Phenotype** | **2** | **3** | **4** | **5** | **6** | **7** | **VAN** | **TEI** | **DALB** | **ORI** |
| ORI_R1 | mecA | 0,5 | 2 | 2 | 4 | 0,0625 | 0,25 | 8 | 32 | 4 | 4 |
| ORI_R2 | mecA | 0,5 | 2 | 1 | 1 | 0,125 | 0,25 | 8 | 16 | 4 | 0,5 |
| ORI_R3 | mecA | 0,25 | 2 | 1 | 2 | 0,0625 | 0,25 | 8 | 32 | 2 | 2 |
| ORI_R4 | mecA | 0,5 | 2 | 0,25 | 0,5 | 0,0625 | 0,25 | 4 | 16 | 0,25 | 0,5 |
| TEI_R1 | mecA | 0,125 | 0,5 | 0,5 | 0,5 | 0,0625 | 0,25 | 4 | 32 | 0,25 | 0,125 |
| TEI_R2 | mecA | 2 | 8 | 0,125 | 0,5 | 0,25 | 0,25 | 8 | 32 | 0,125 | 0,125 |
|  |  |  |  |  |  |  |  |  |  |  |  |
| ***S.haemolyticus*** | **Phenotype** | **2** | **3** | **4** | **5** | **6** | **7** | **VAN** | **TEI** | **DALB** | **ORI** |
| DALB_R1 | mecA | 4 | 8 | 8 | 8 | 0,5 | 1 | 32 | 128 | 2 | 0,25 |
| DALB_R2 | mecA | 2 | 8 | 8 | 8 | 0,25 | 1 | 64 | 256 | 8 | 0,25 |
| DALB_R3 | mecA | 2 | 8 | 8 | 8 | 0,25 | 2 | 8 | 32 | 2 | 0,25 |
| DALB_R4 | mecA | 2 | 8 | 8 | 8 | 0,125 | 2 | 32 | 64 | 2 | 0,0625 |
| DALB_R5 | mecA | 0,5 | 2 | 1 | 2 | 0,25 | 0,5 | 16 | 128 | 1 | 0,125 |
| ORI_R1 | mecA | 1 | 8 | 8 | 8 | 0,25 | 0,5 | 16 | 64 | 8 | 0,5 |

MIC: minimal inhibitory concentration. MICs of the compounds against glycopeptide antibiotic resistant strains are highlighted in red. ^a^VAN, vancomycin; TEI: teicoplanin; DALB: dalbavancin, ORI: oritavancin.


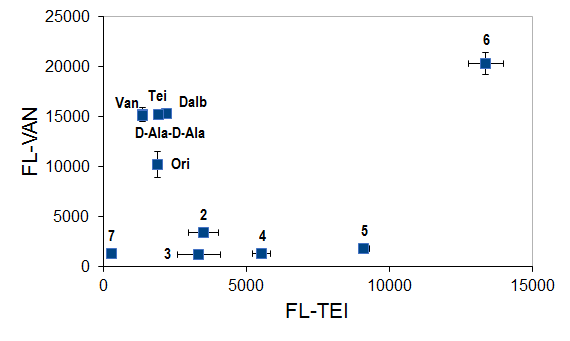


Figure S16. Summary of fluorescent data. Relative fluorescence of FL-VAN (Y axis) versus FL-TEI (X-axis), released by non-fluorescent glycopeptides from live *S. aureus* cells, saturated by FL-VAN or FL-TEI. (VAN, vancomycin; TEI: teicoplanin; DALB: dalbavancin, ORI: oritavancin.)

Table S6. Antibacterial activity of compounds **6**, **7** and **24** against Gram-negative strains using teicoplanin as a reference antibiotic

| MIC in μg/mL | | | | |
| --- | --- | --- | --- | --- |
| Bacterial strains | Compounds | | | |
|  | teicoplanin | **6** | **7** | **24** |
| *Klebsiella* *pneumoniae* ST258 clone K 160/09 | 256 | 128 | 512 | 128 |
| *Pseudomonas* *aeruginosa* ATCC 27853 | 256 | 256 | 128 | 25.6 |
| *Acinetobacter* *baumannii* ATCC BAA1605 | 128 | 128 | 128 | 25.6 |
| *Escherichia* *coli* ATCC 25218 | 256 | 128 | 256 | 3.2 |

MIC: minimum inhibitory concentration.

# NMR spectra

## NMR spectra of **4**

^1^H NMR, 500 MHz, DMSO-d6


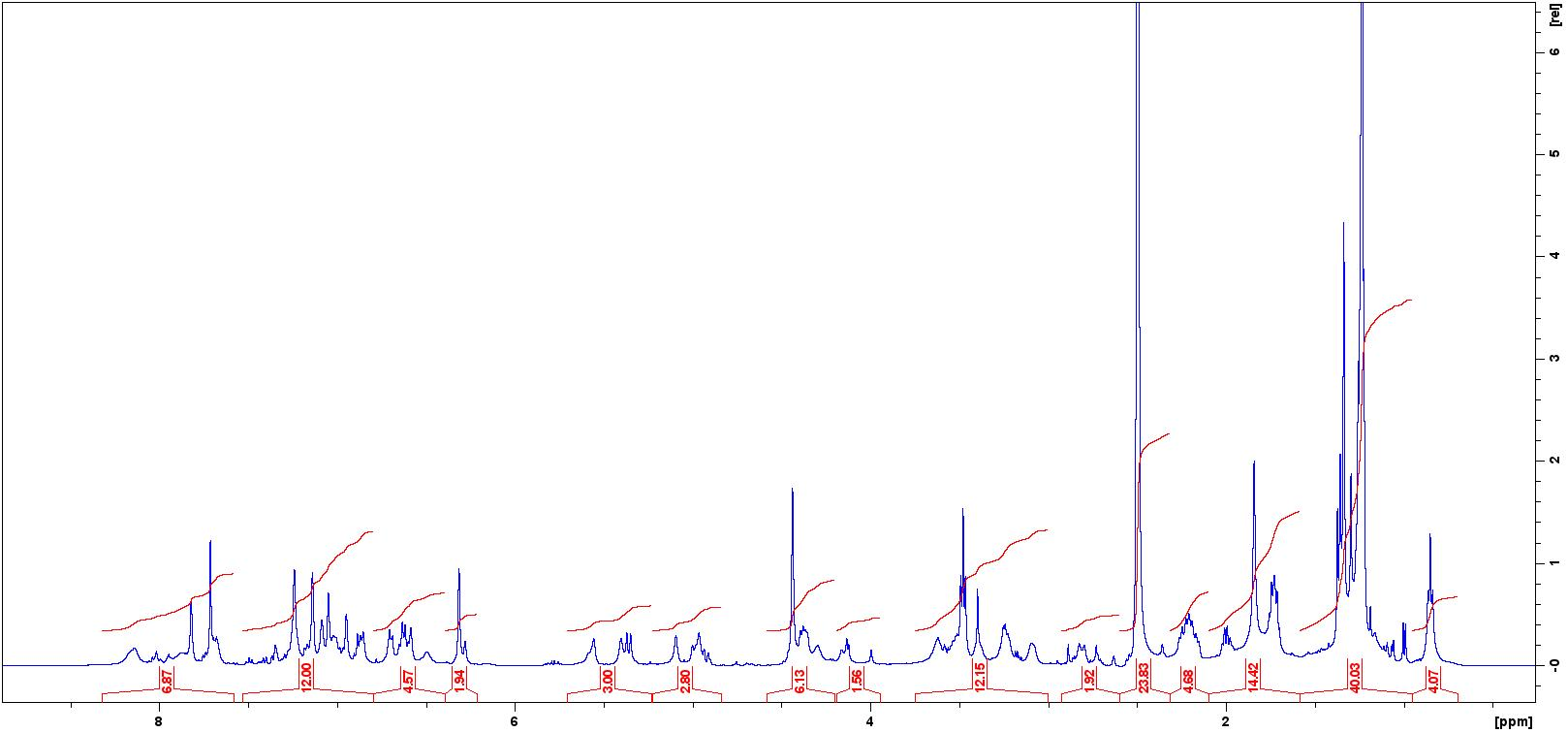


^13^C, 125 MHz, DMSO-d6


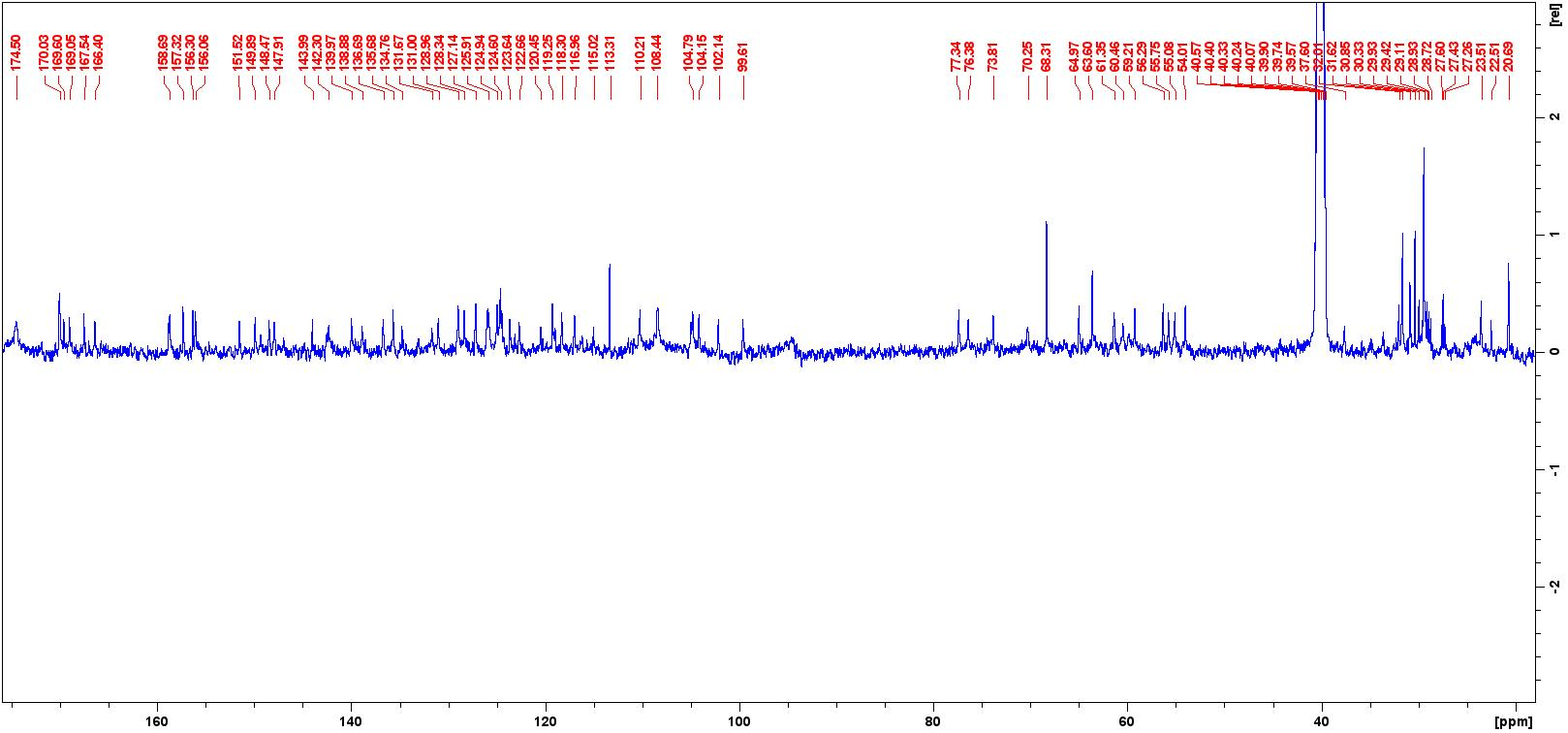


HSQC, 125 MHz, DMSO-d6


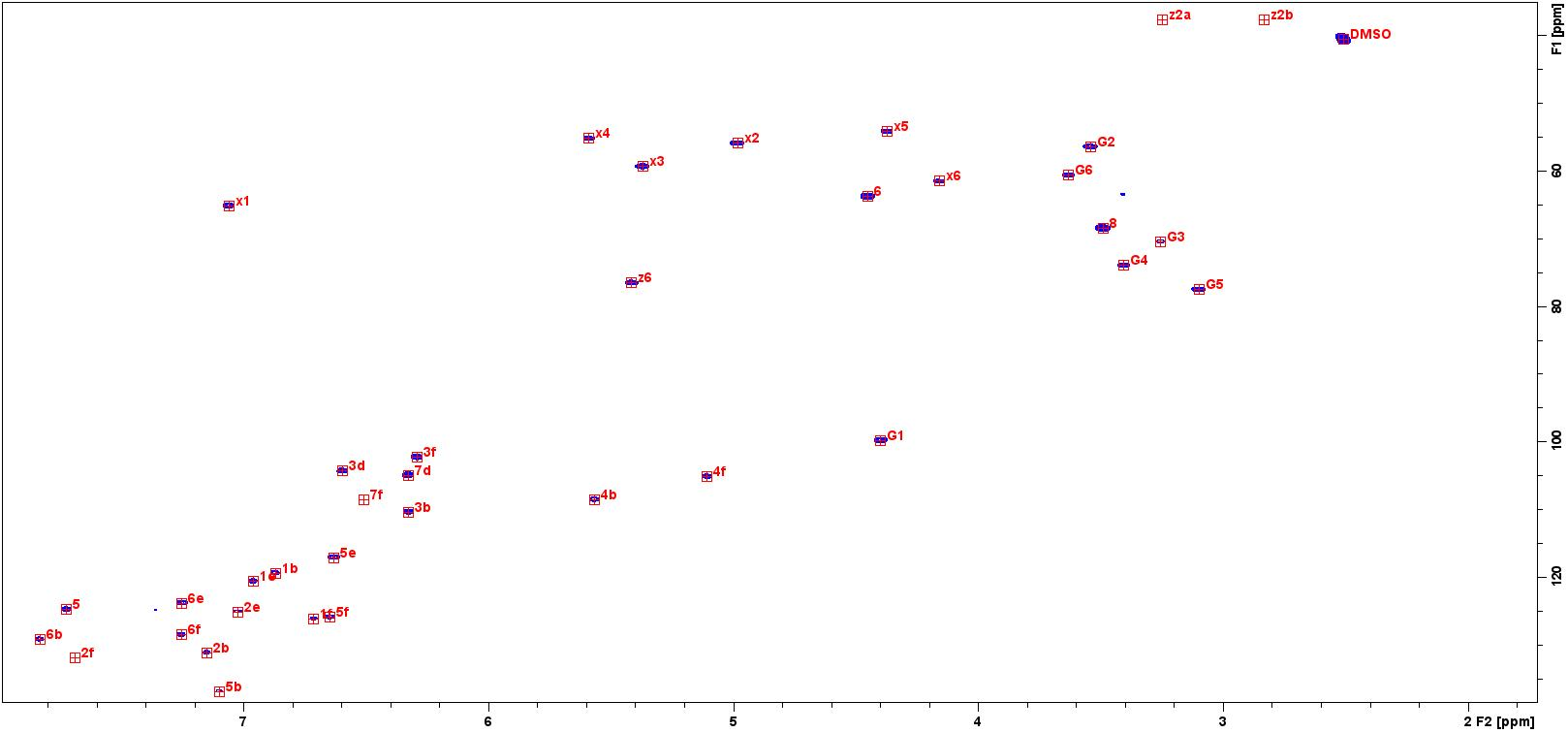


## NMR spectra of **5**

^1^H NMR, 500 MHz, DMSO-d6


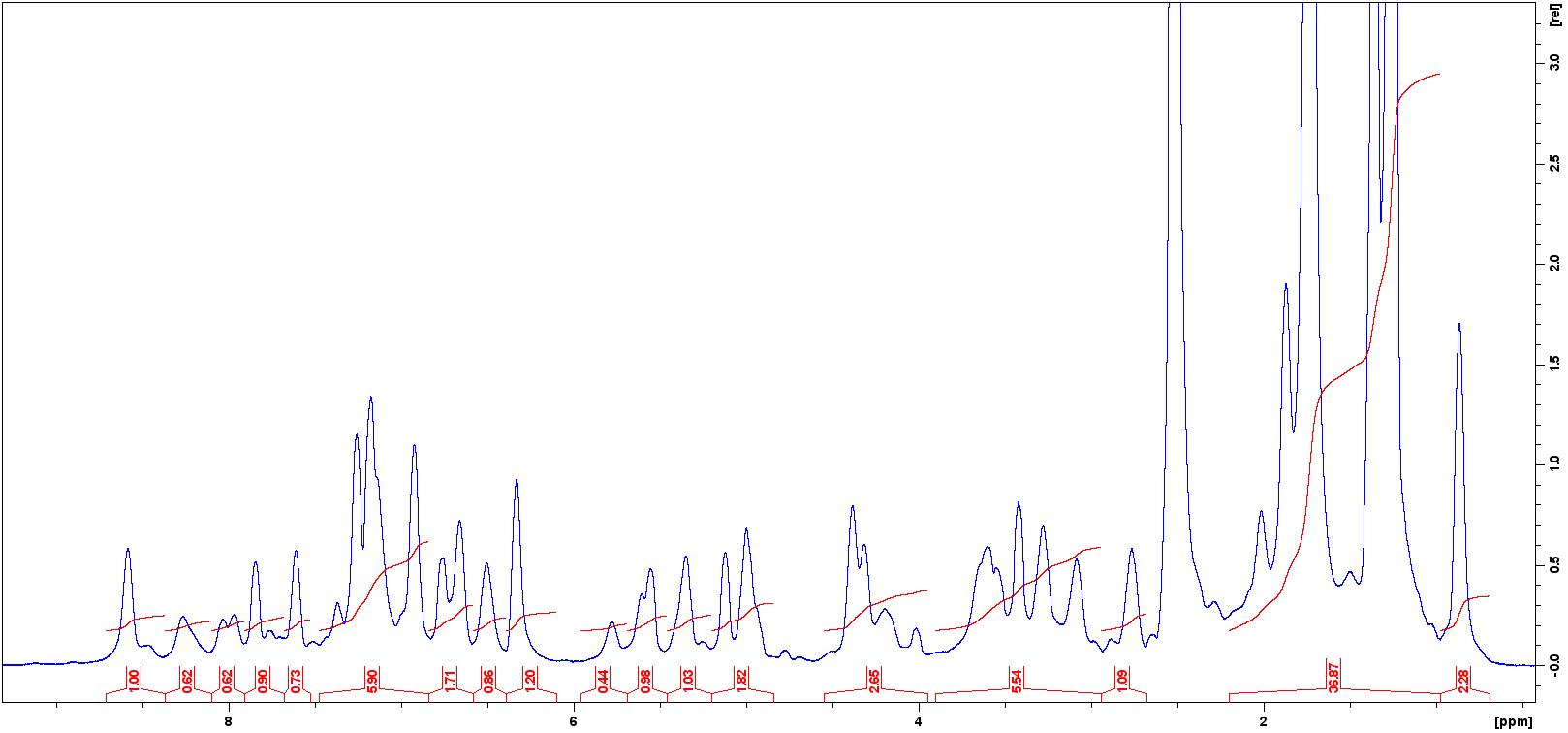


^13^C NMR, 125 MHz, DMSO-d6


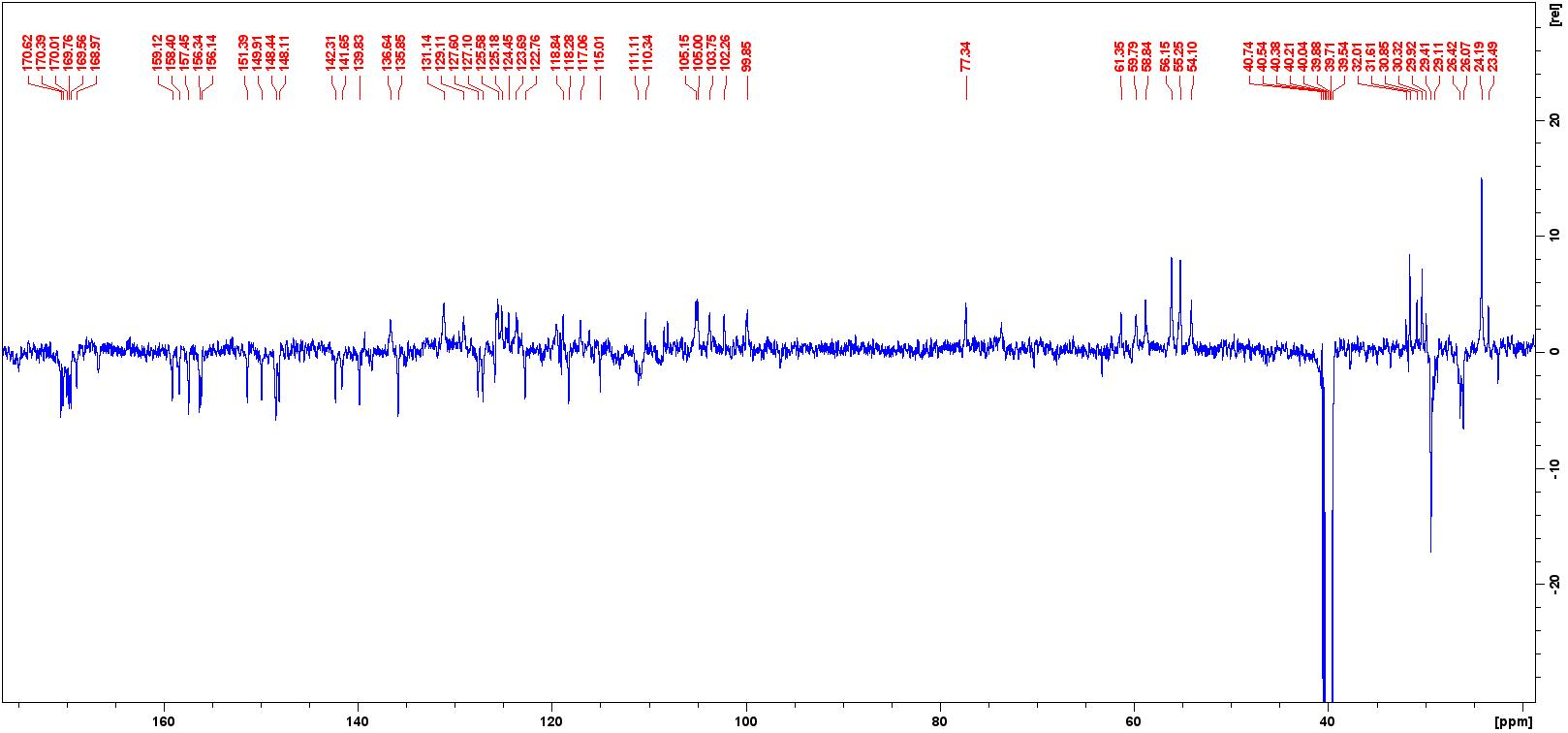


HSQC NMR, 125 MHz, DMSO-d6


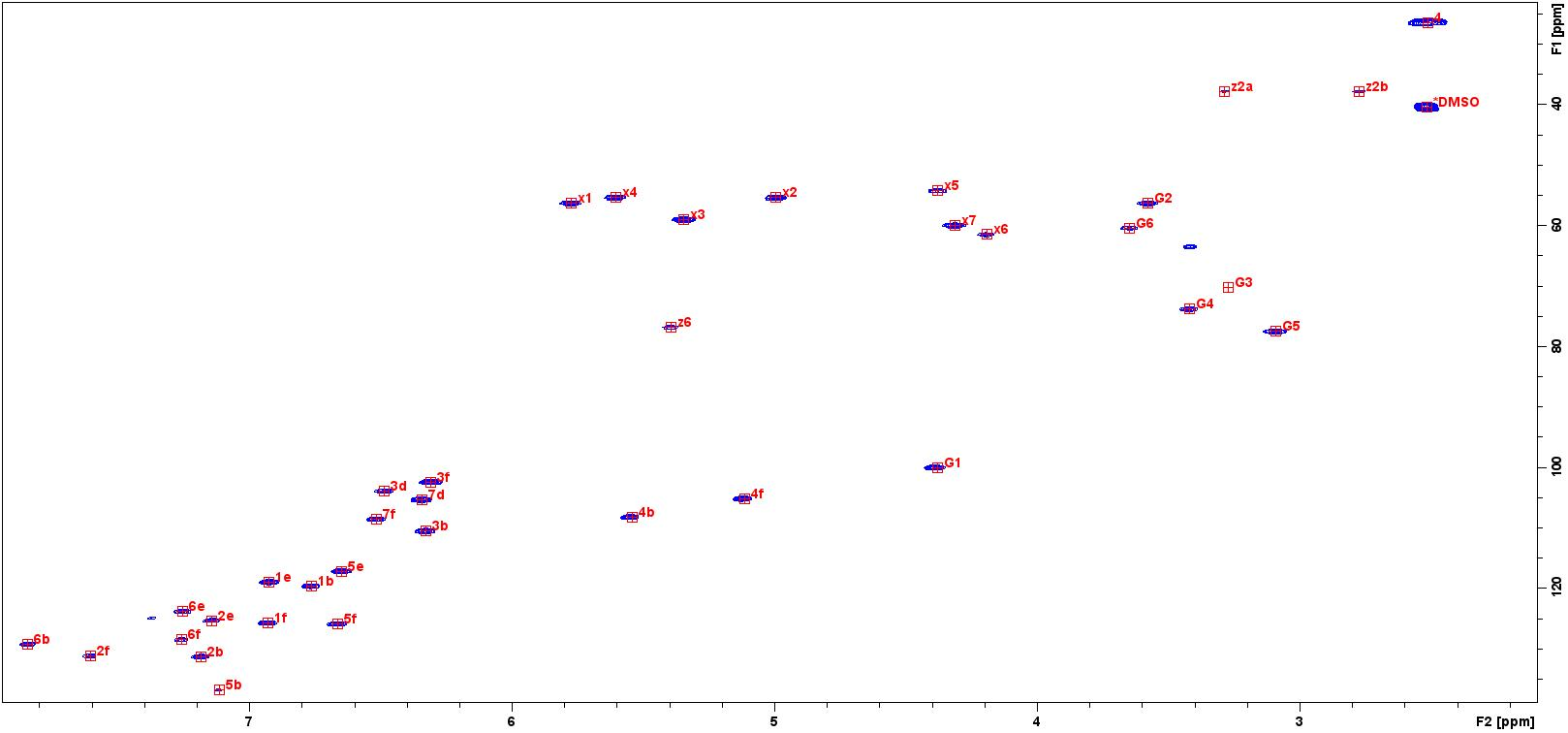


## NMR spectra of **7**

^1^H NMR, 500 MHz, DMSO-d6


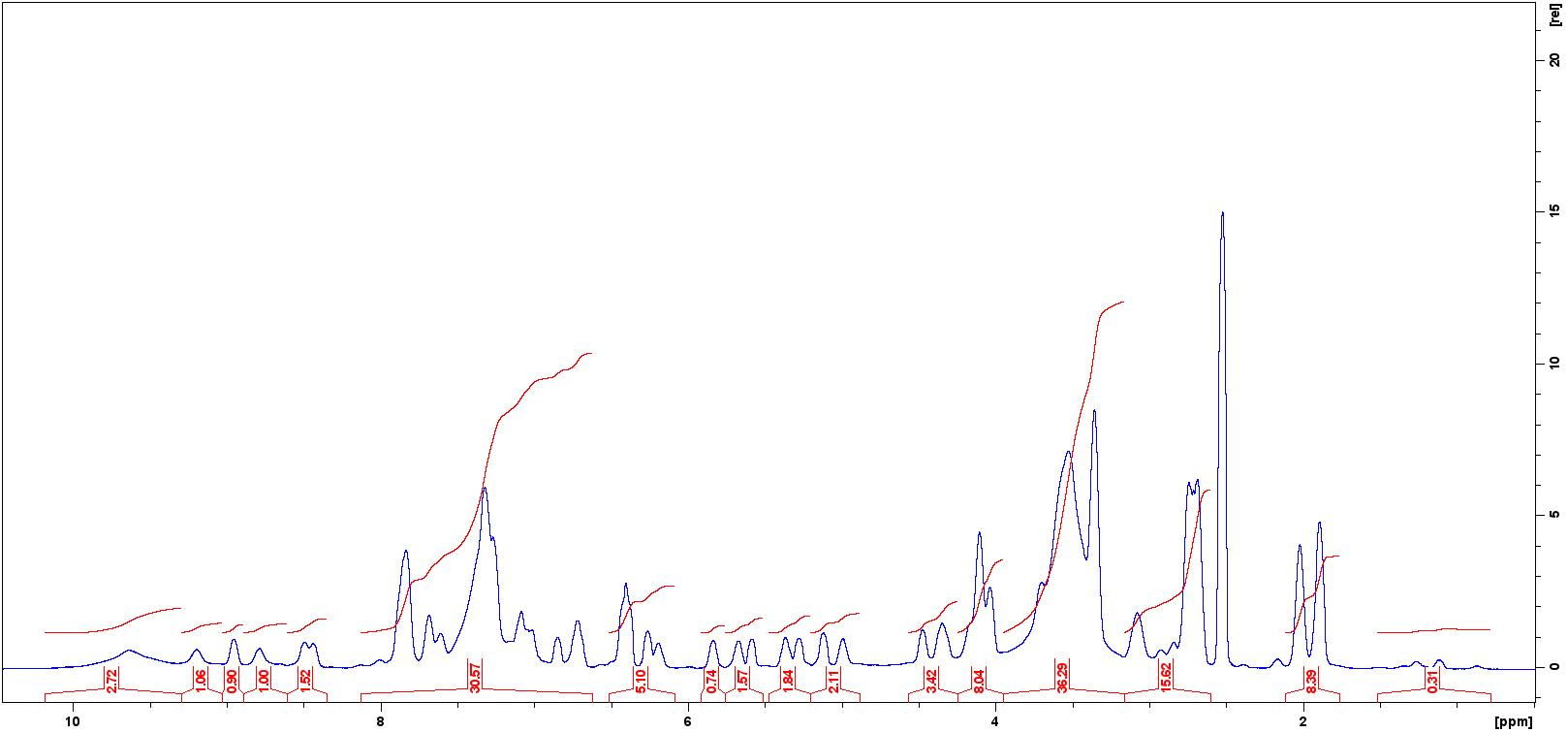


^13^C NMR, 125 MHz, DMSO-d6


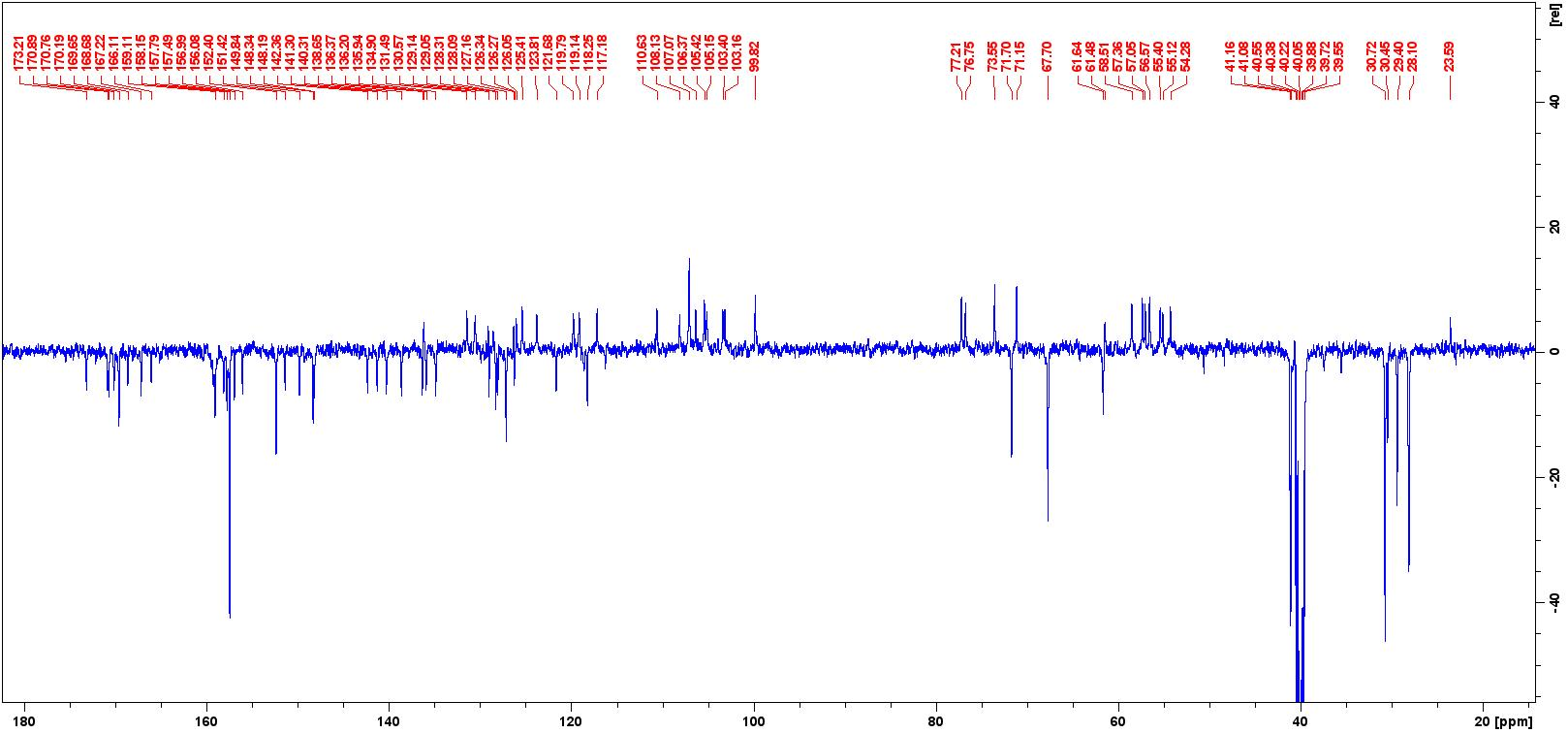


HSQC NMR, 125 MHz, DMSO-d6


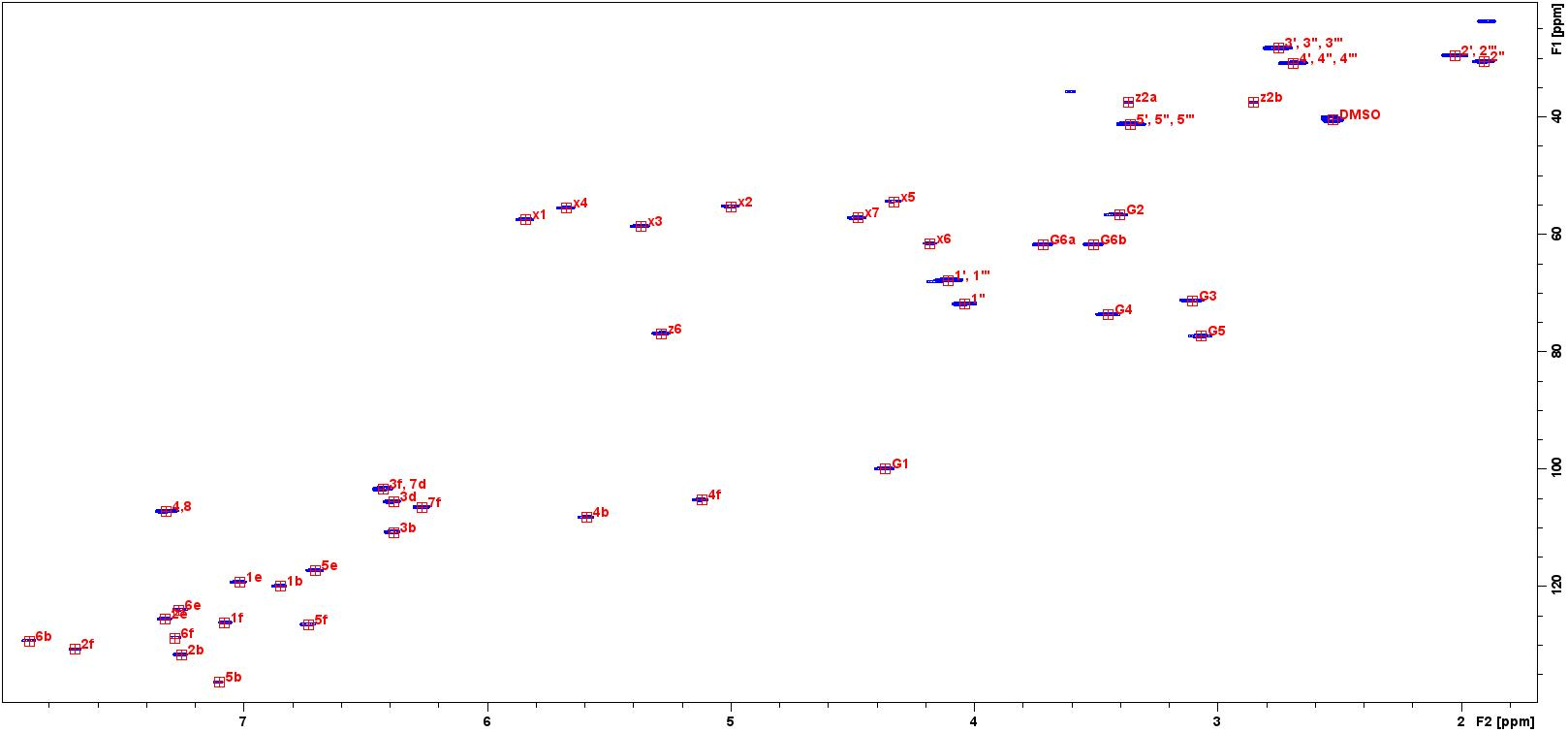


## NMR spectra of **8**


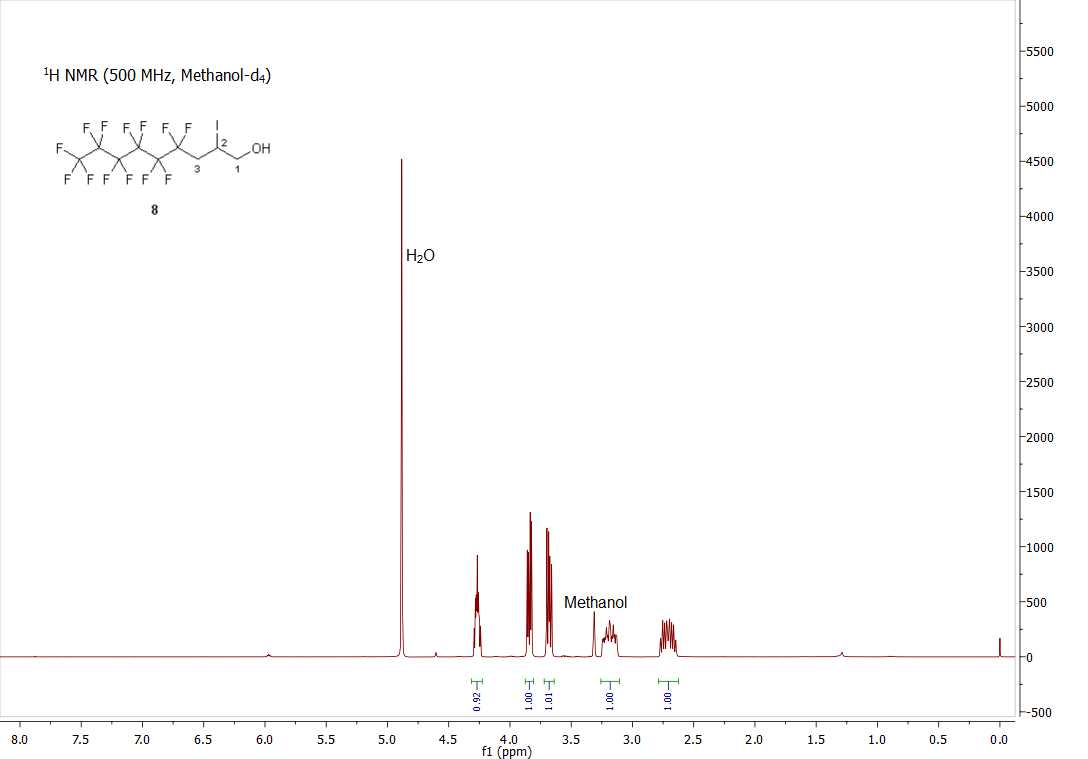


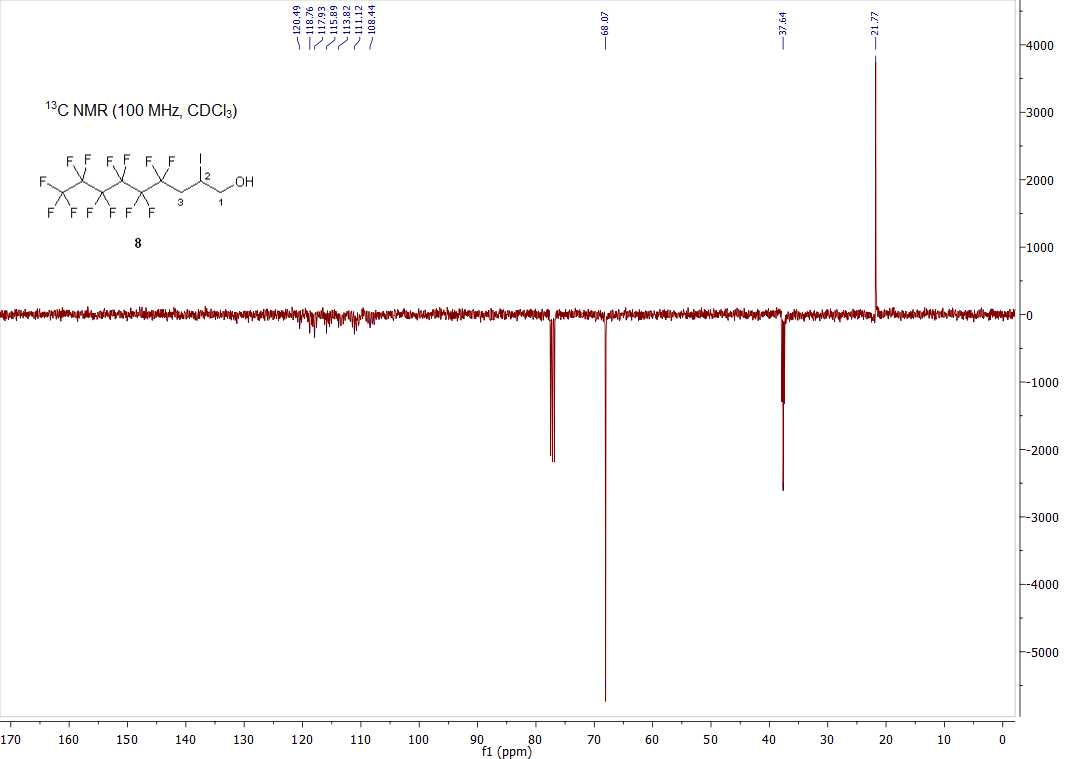


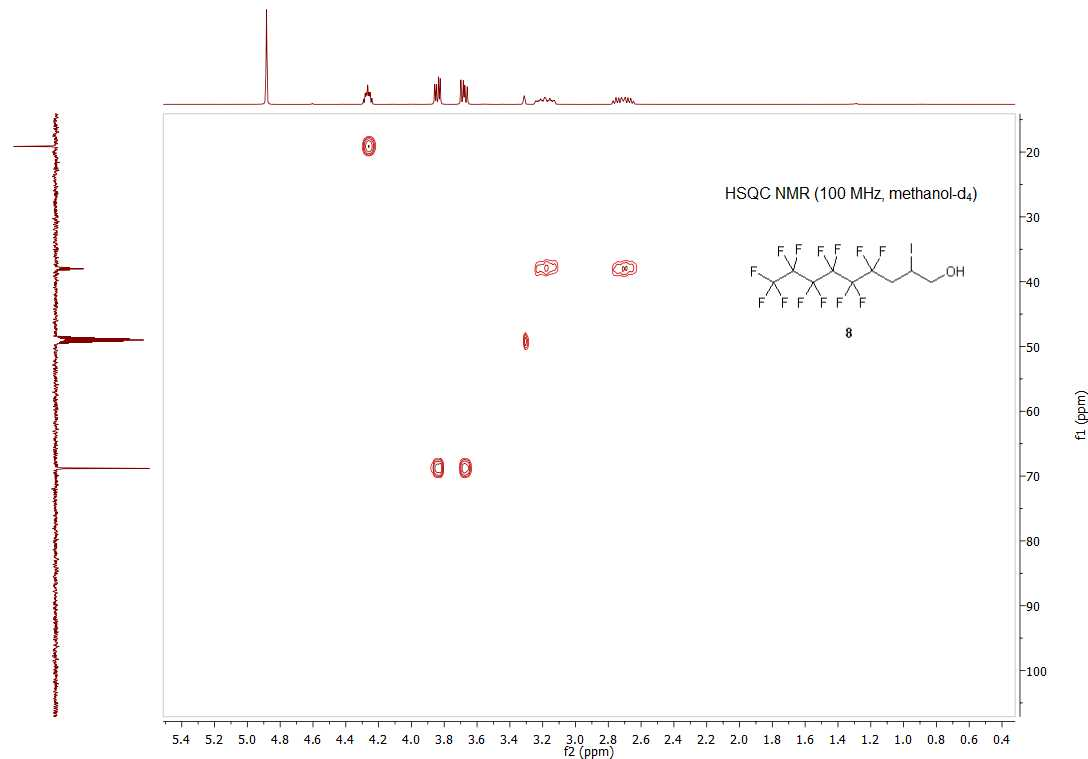


## NMR spectra of **9**


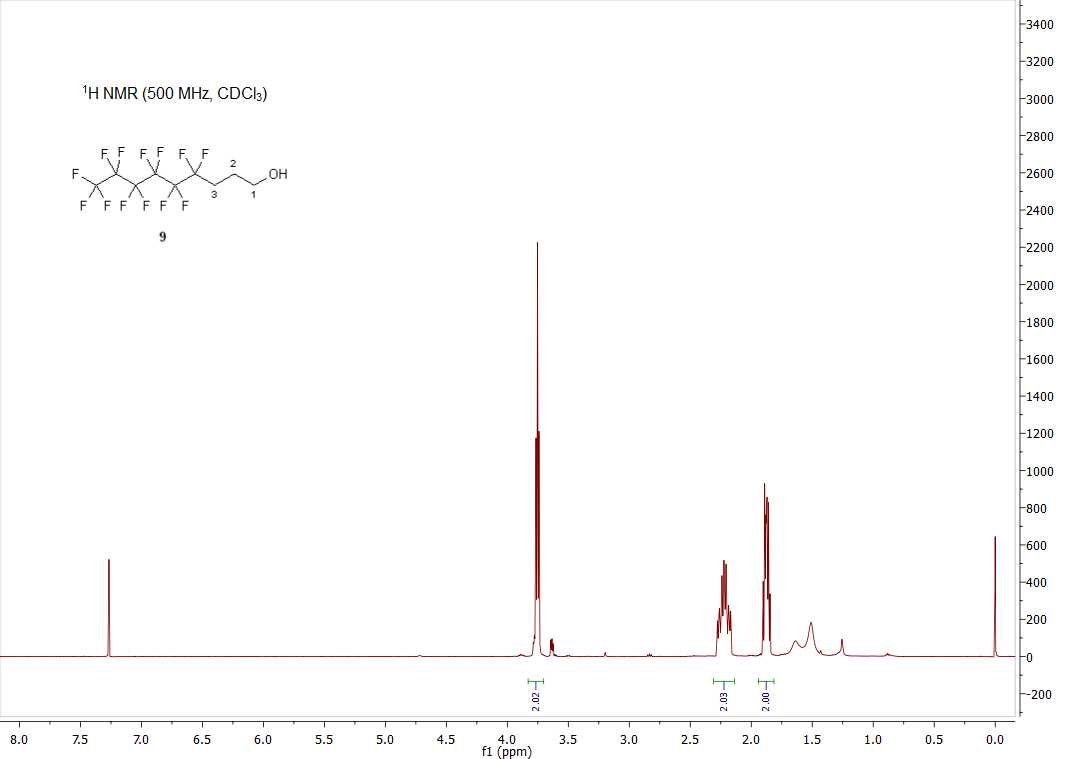


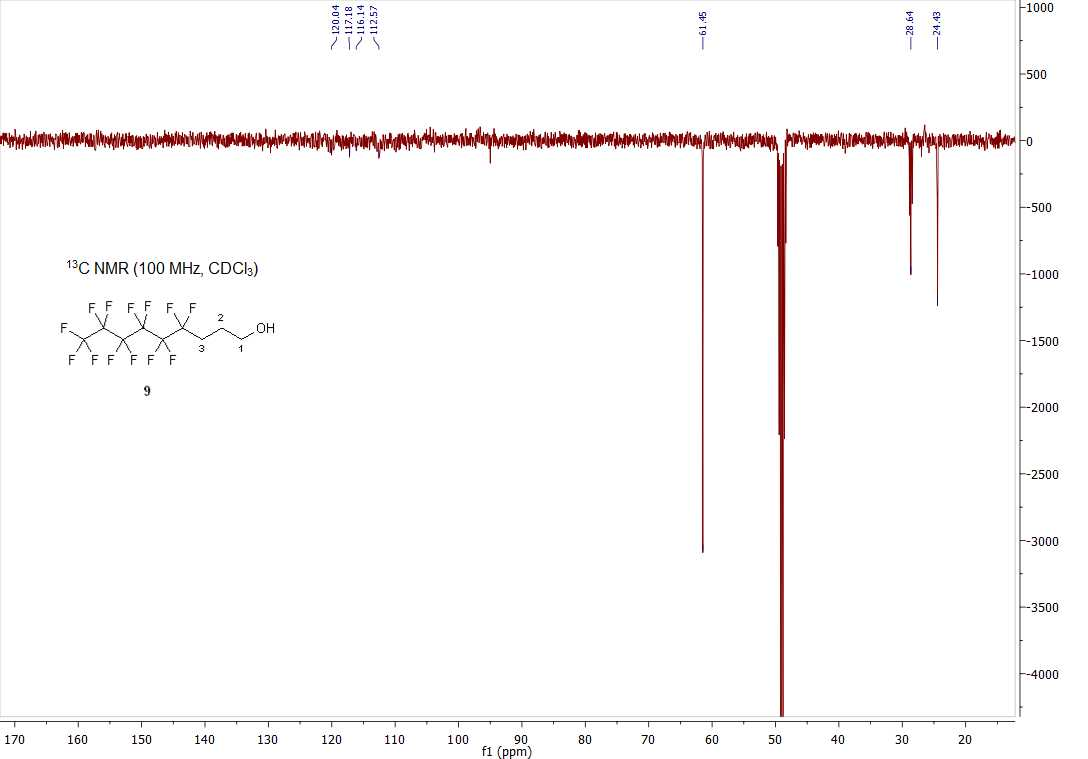


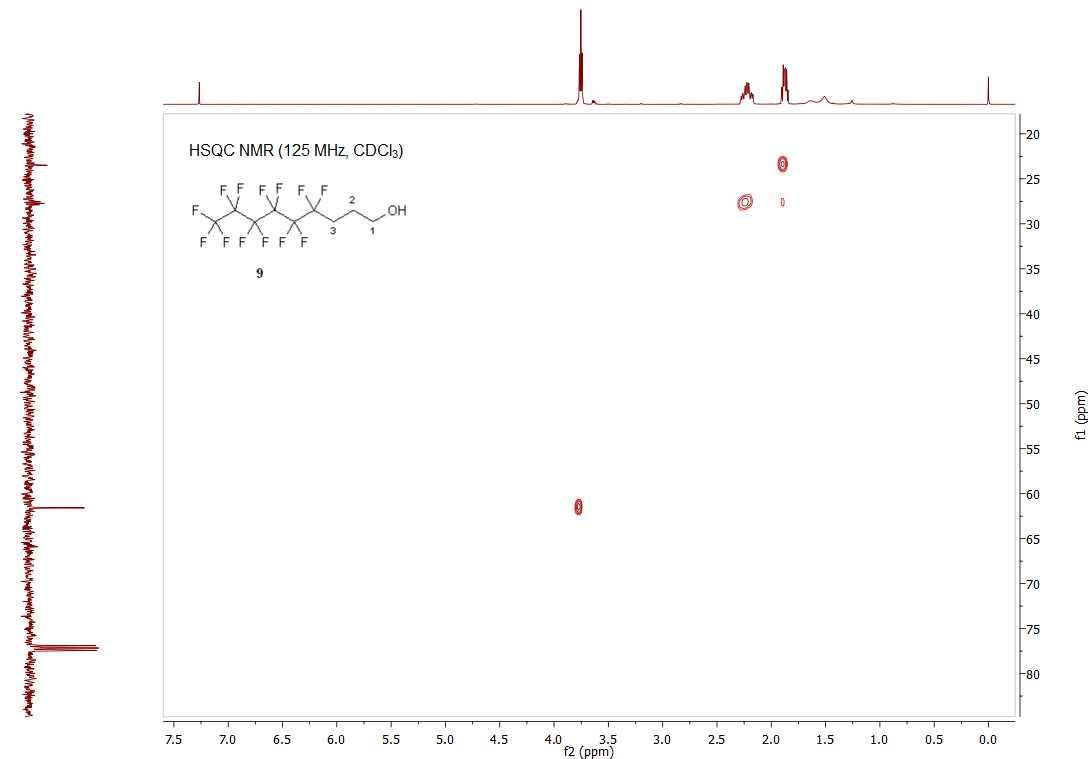


## NMR spectra of **10**


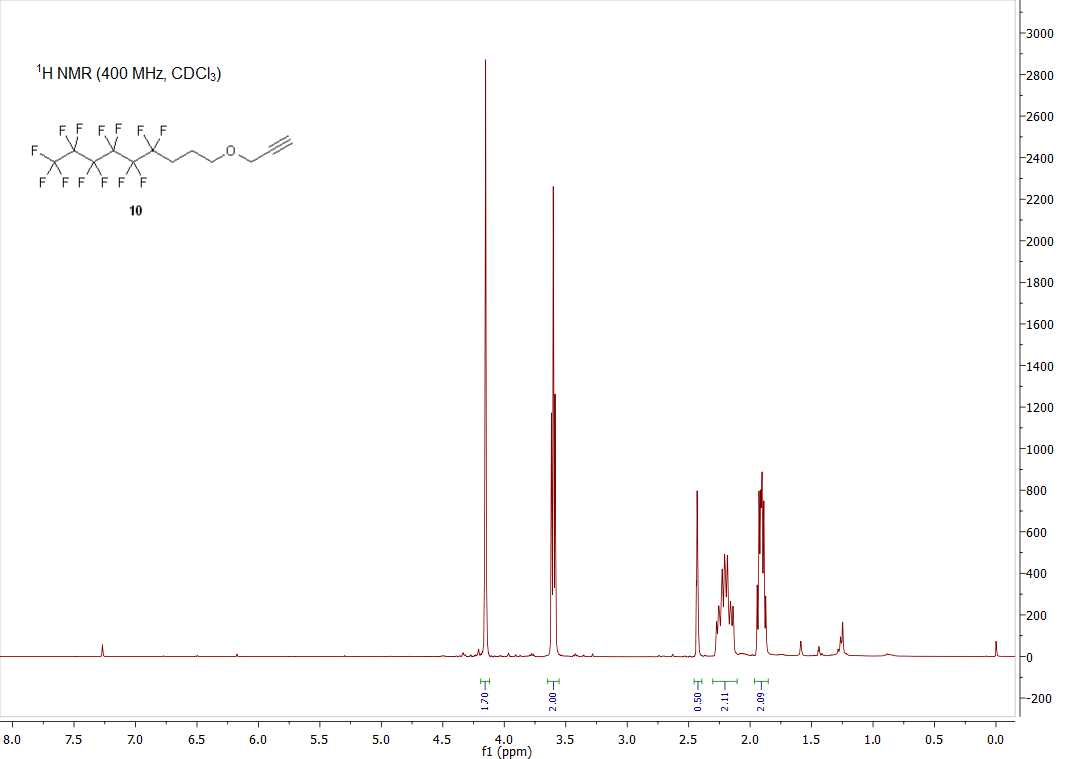


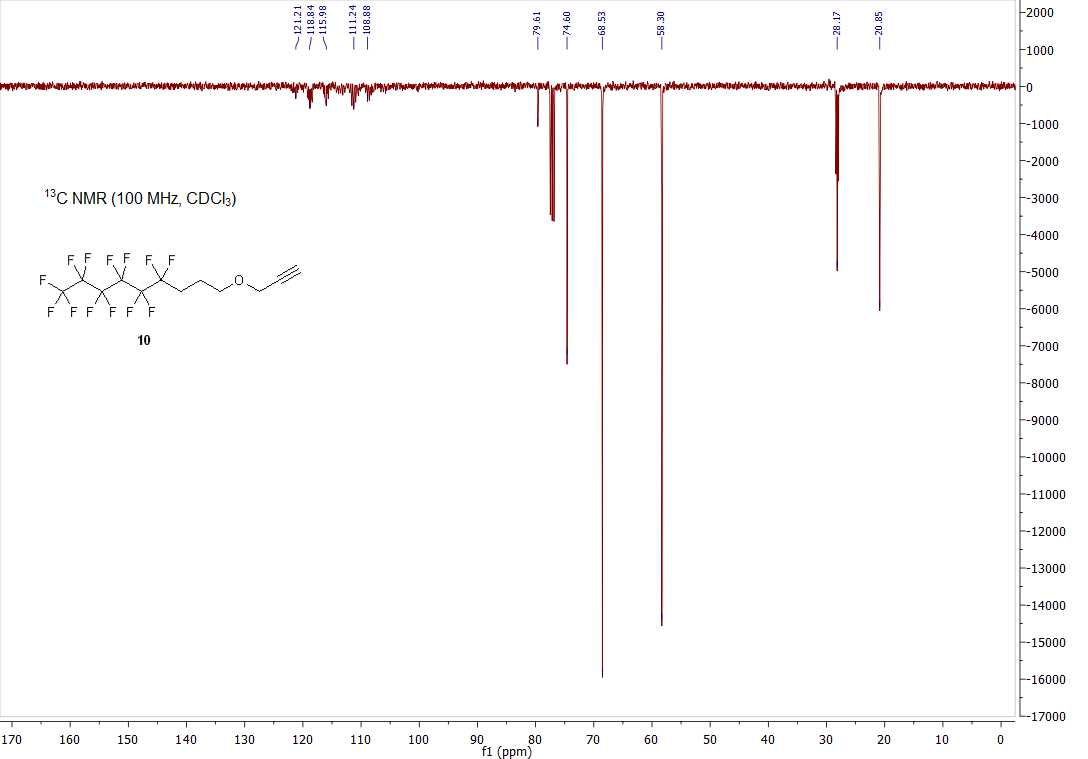


## NMR spectra of **12**


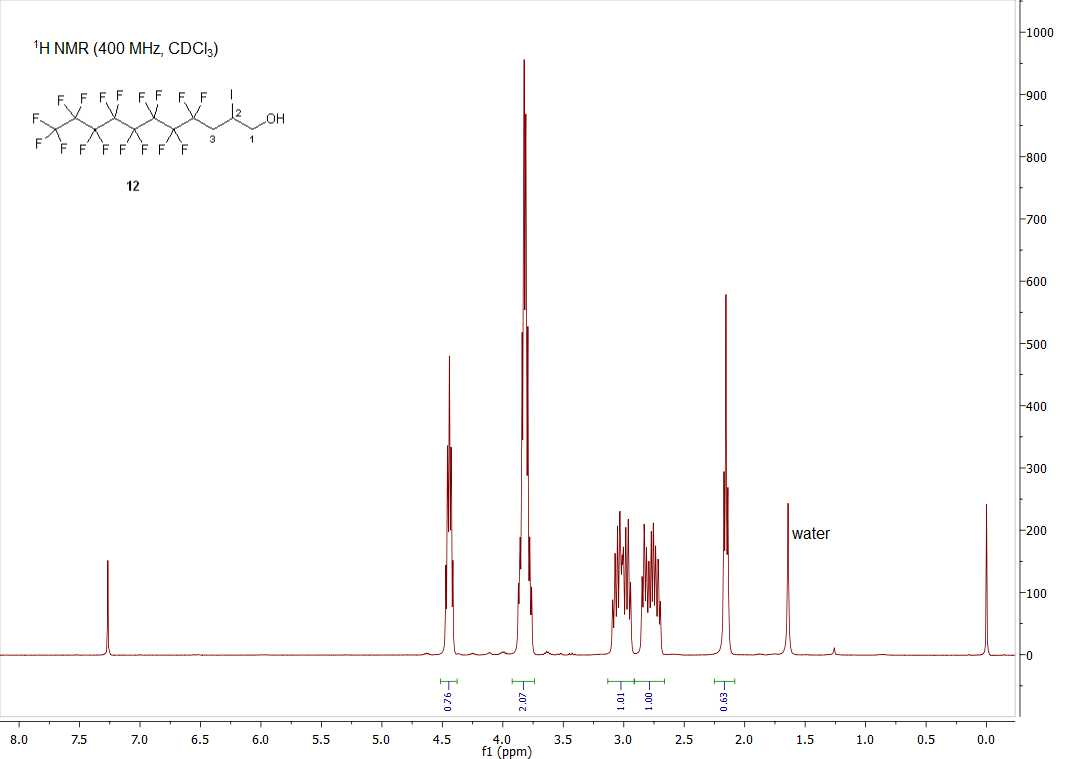


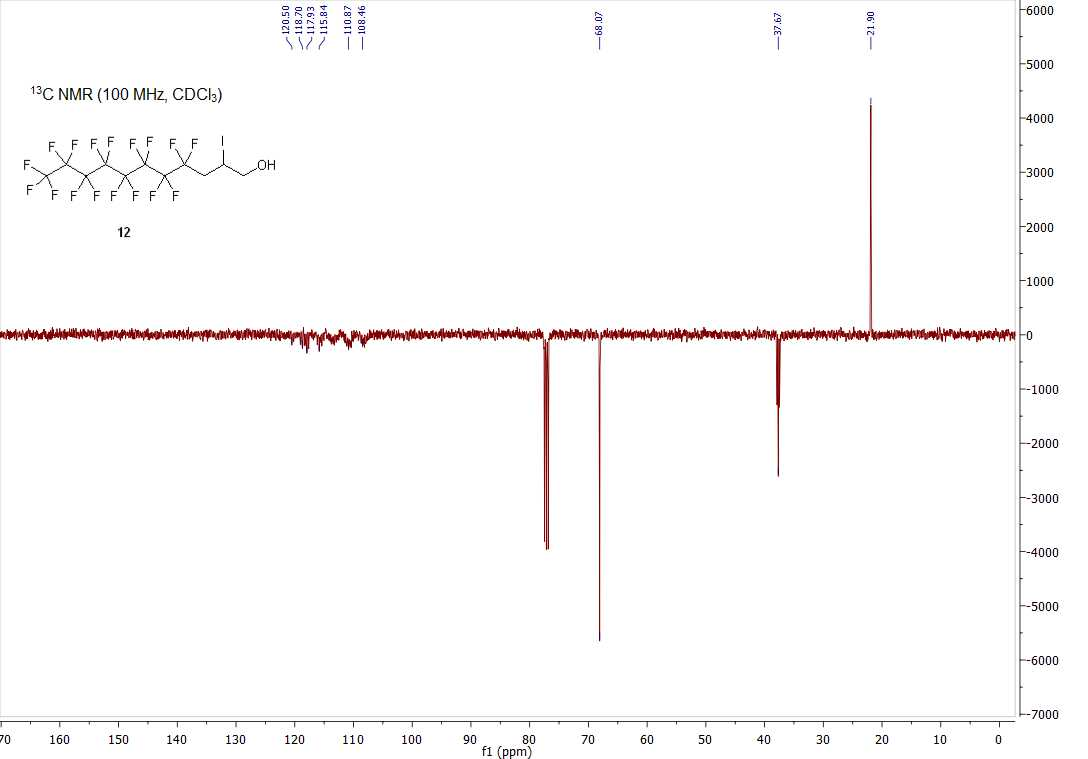


## NMR spectra of **13**


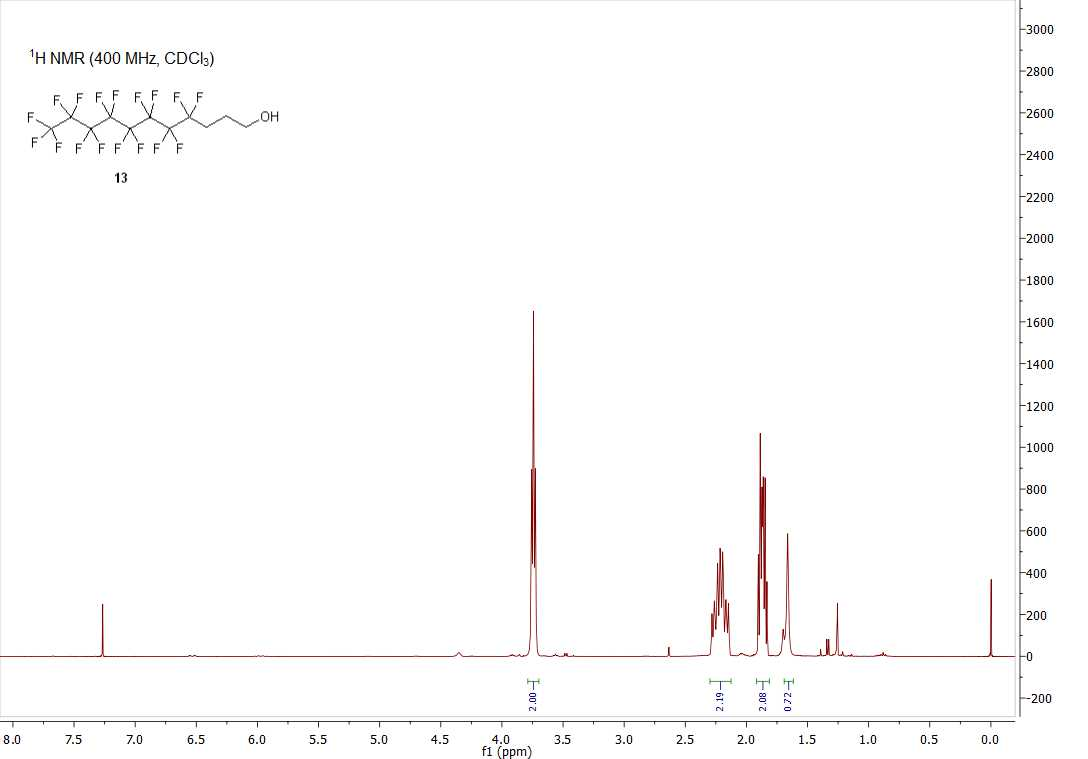


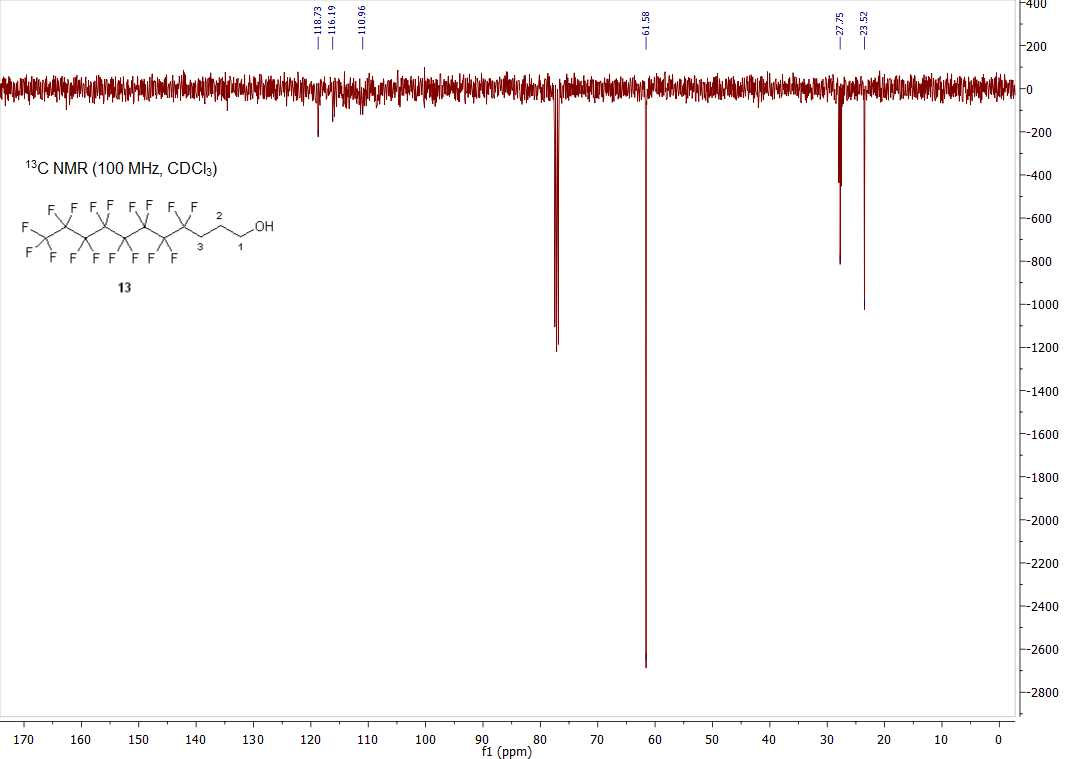


## NMR spectra of **14**


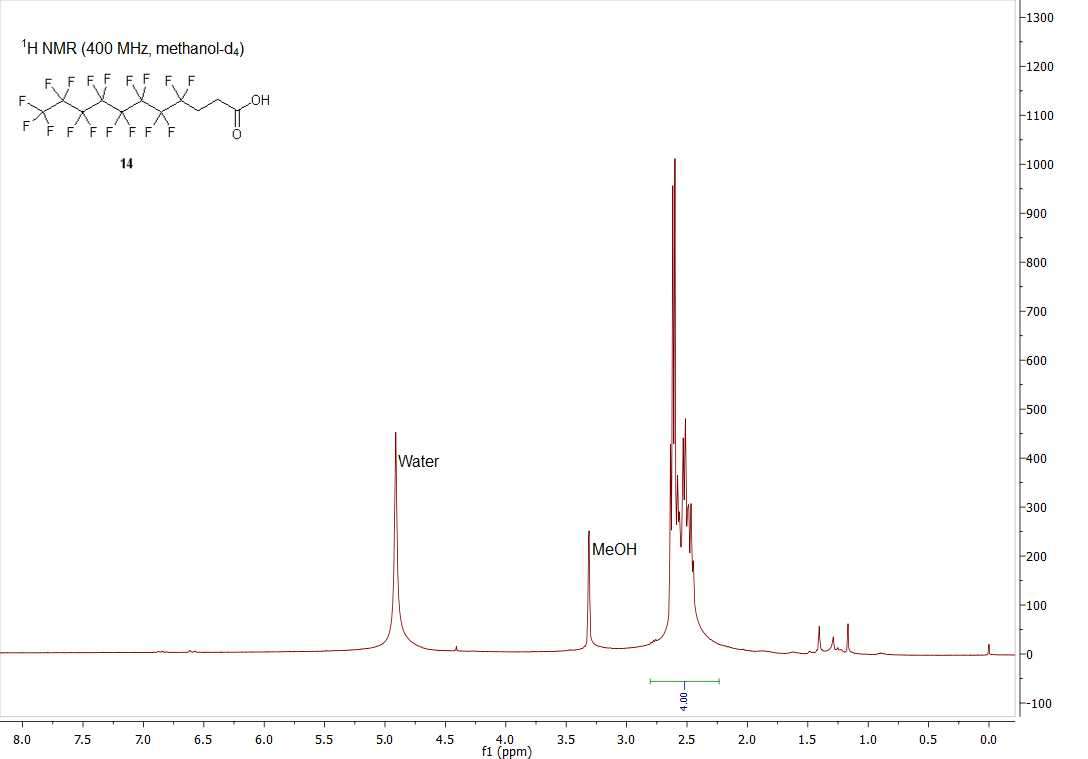


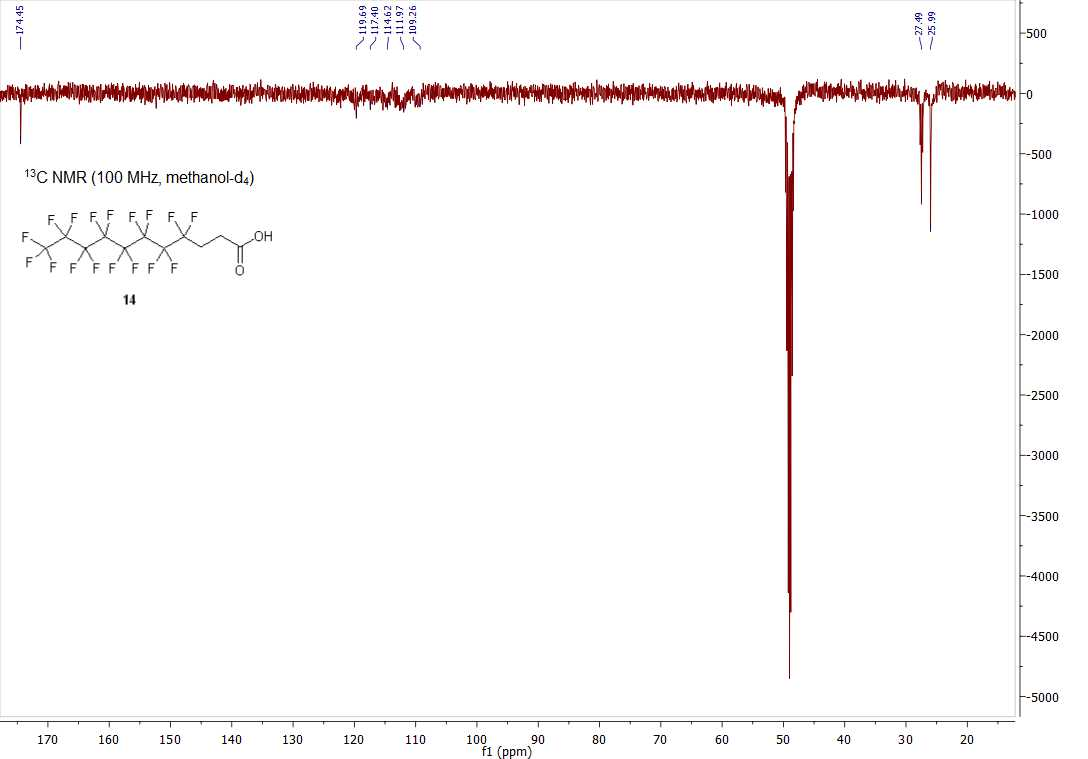


## NMR spectra of **15**


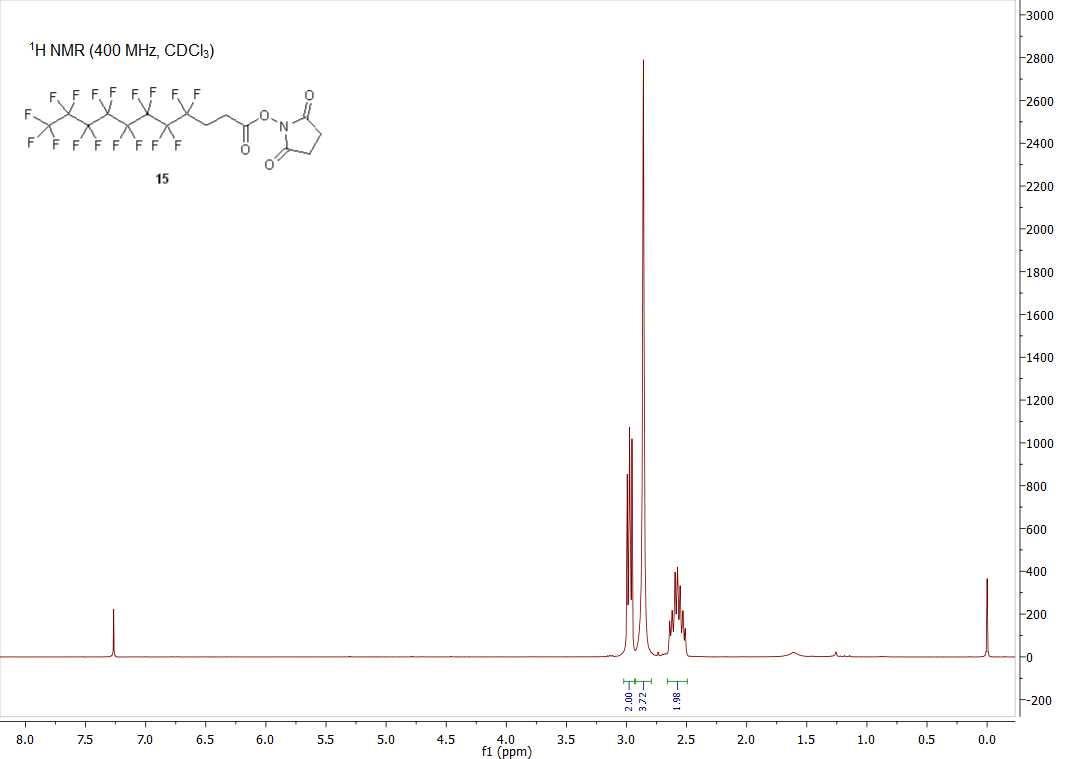


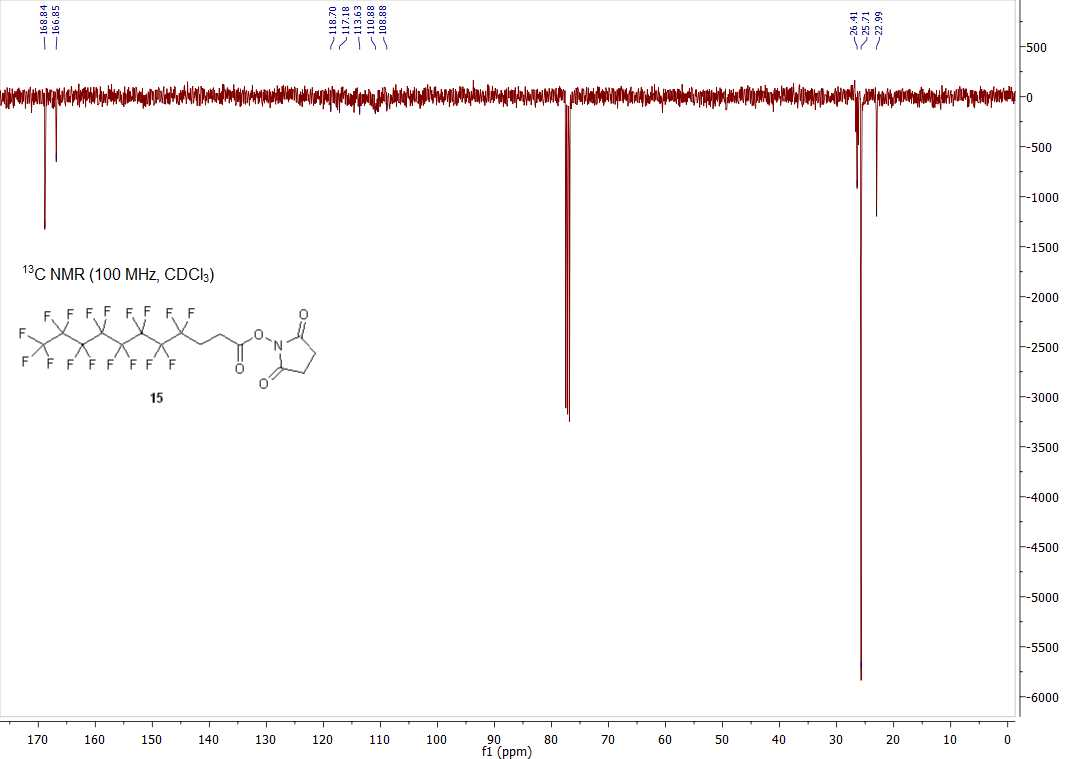


## NMR spectra of **18**


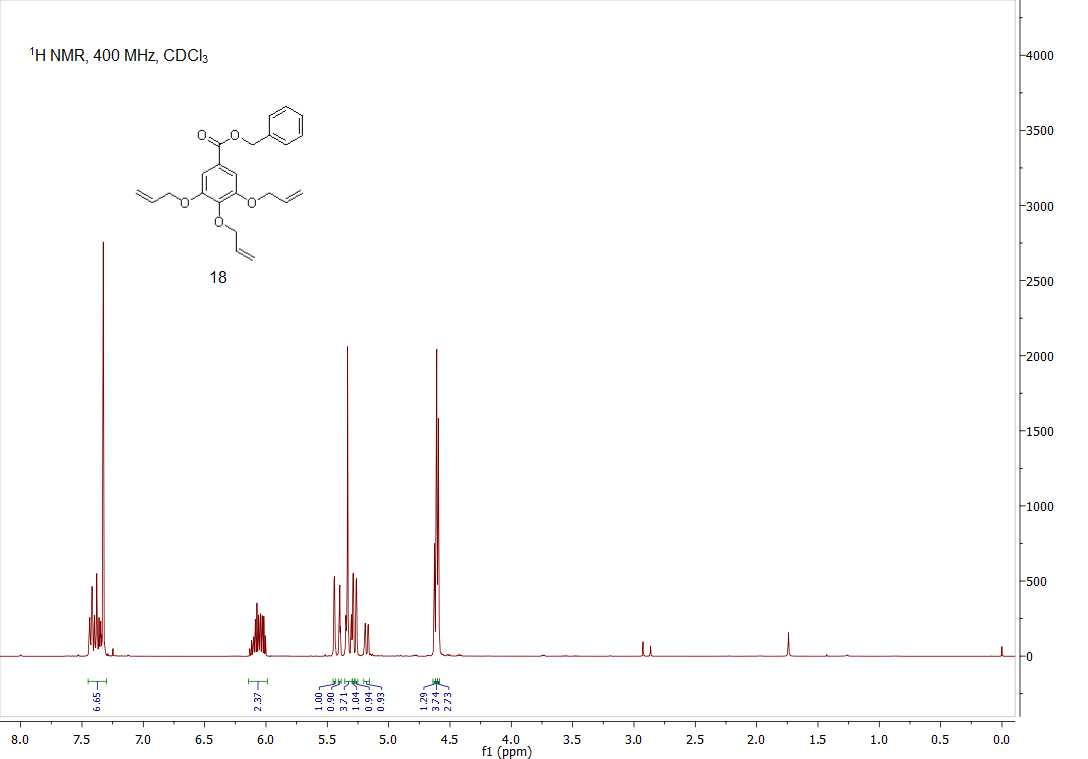


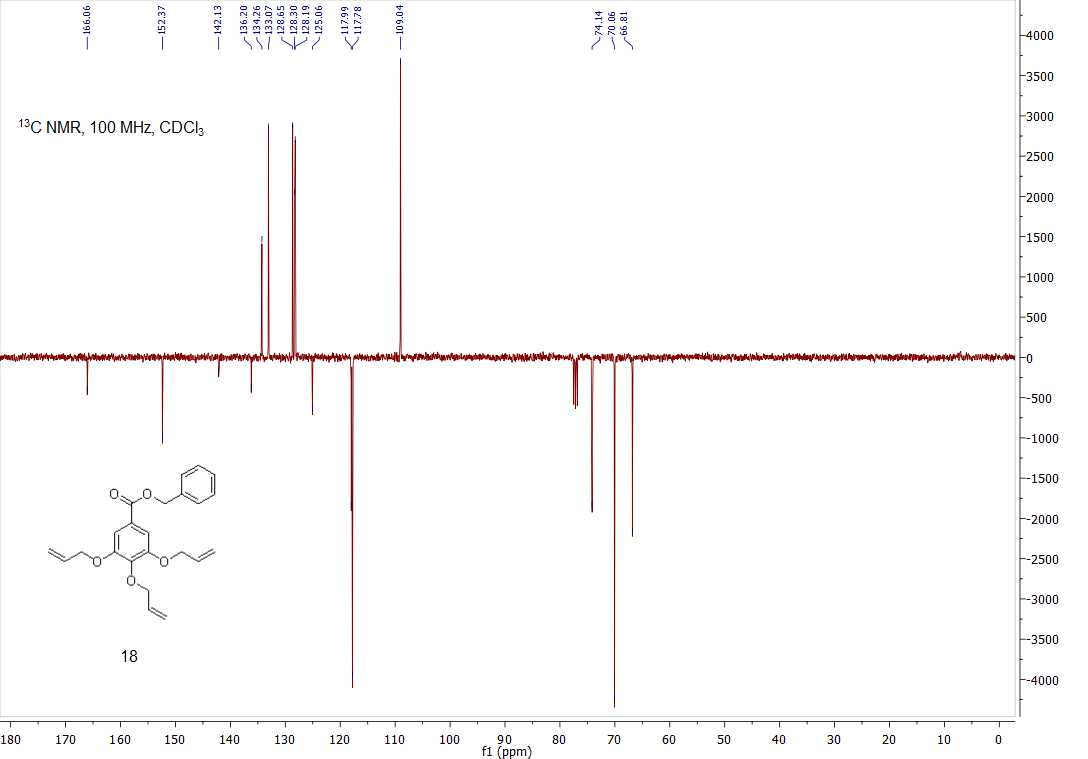


## NMR spectra of **19**


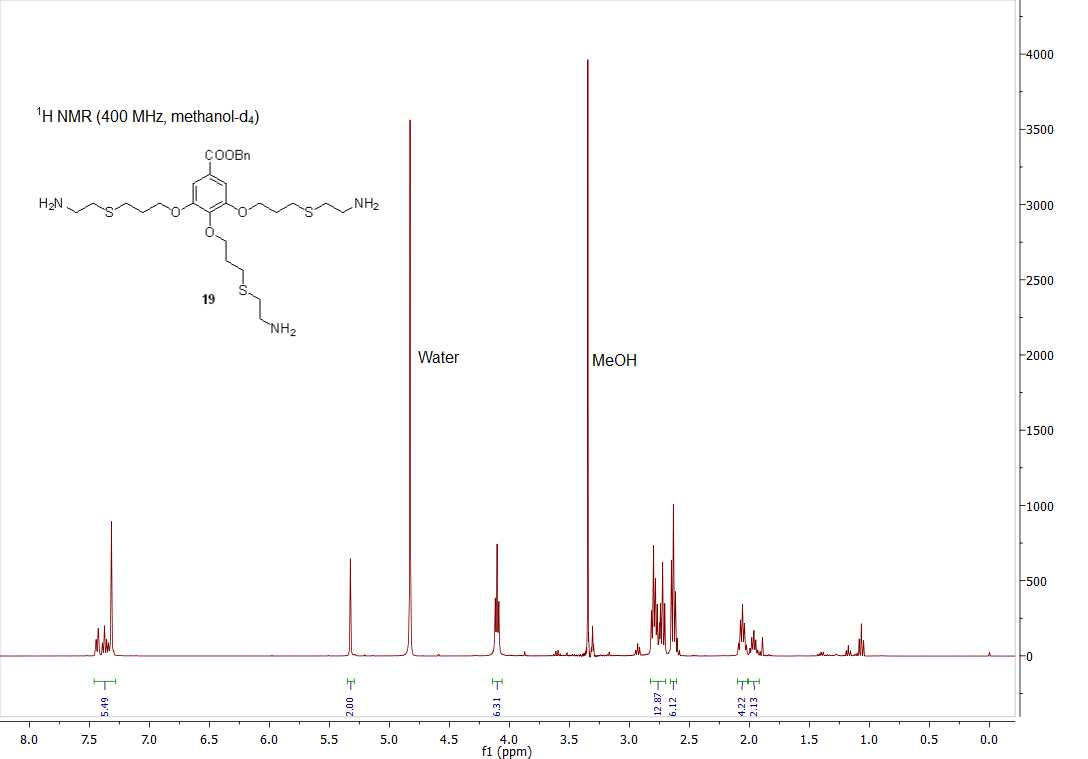


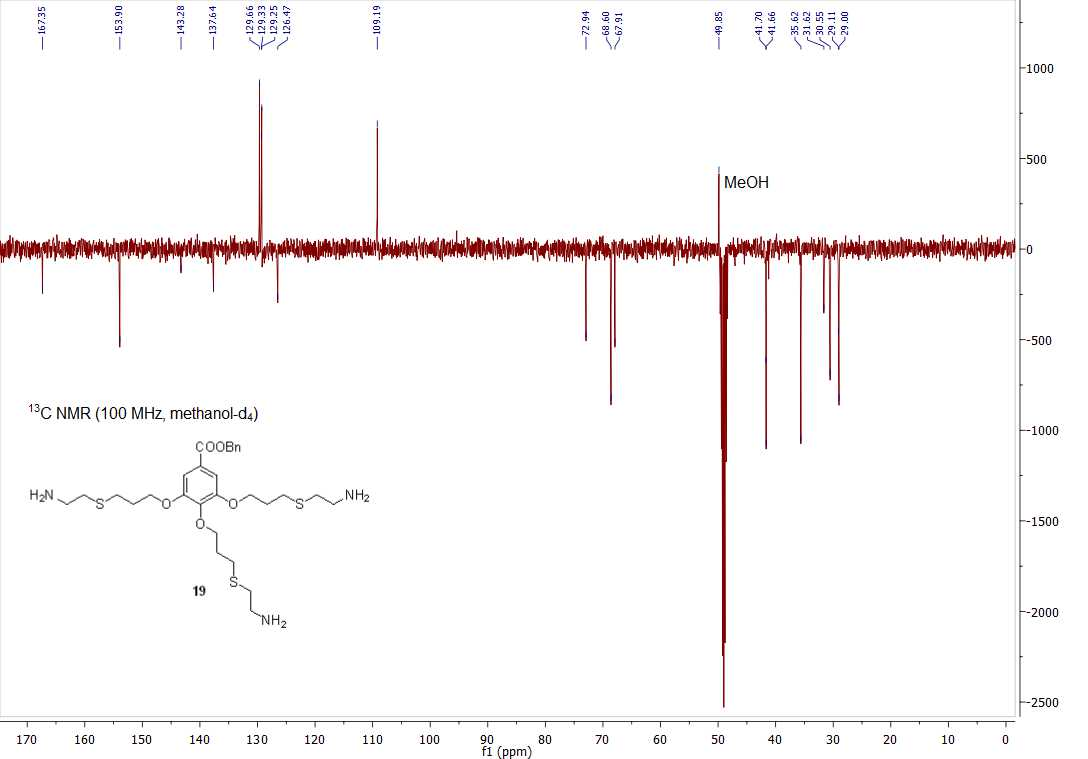


## NMR spectrum of **21**


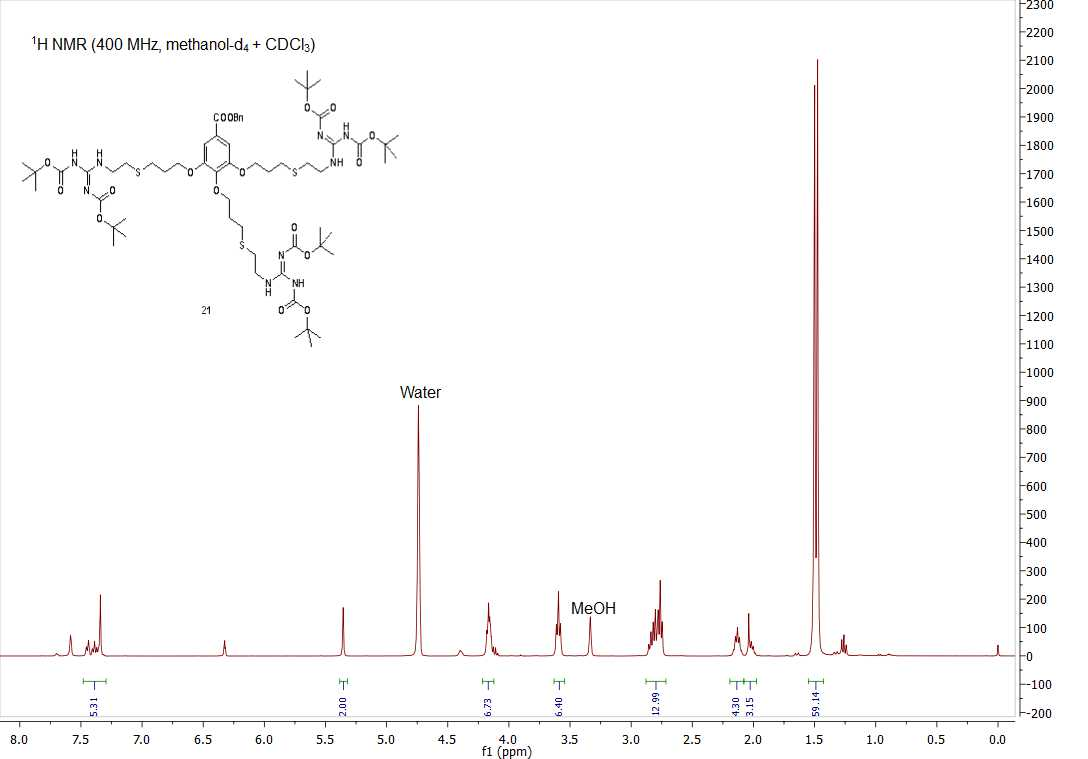


## NMR Spectra of **22**


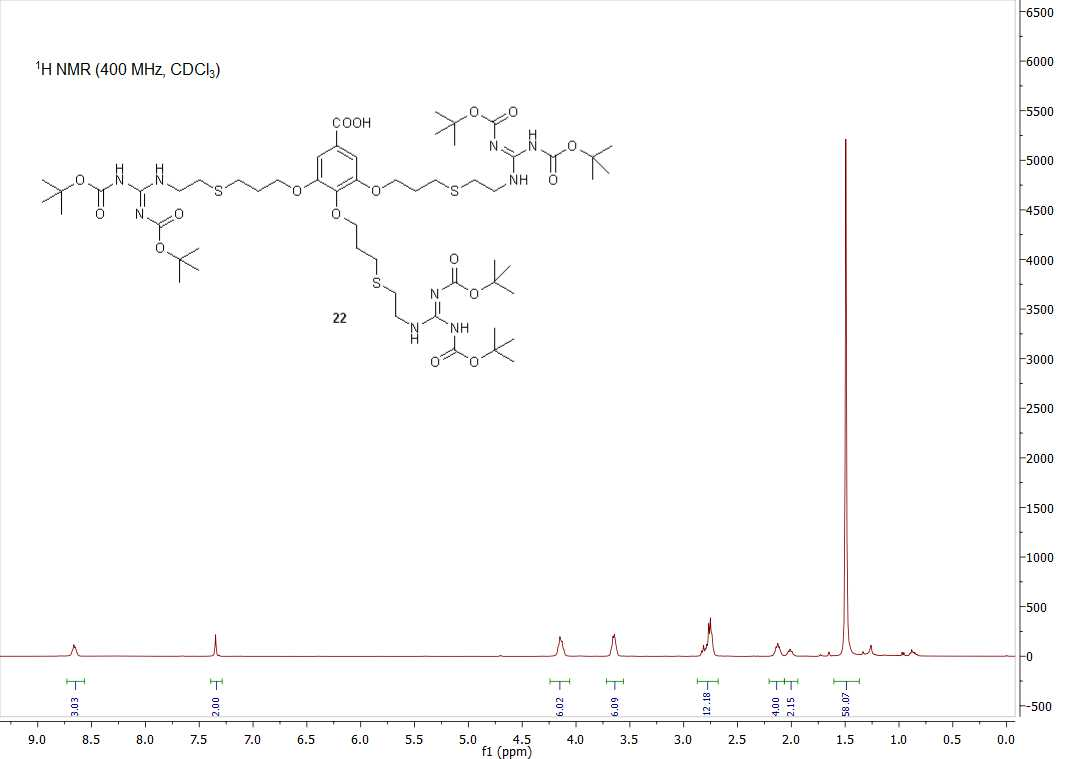


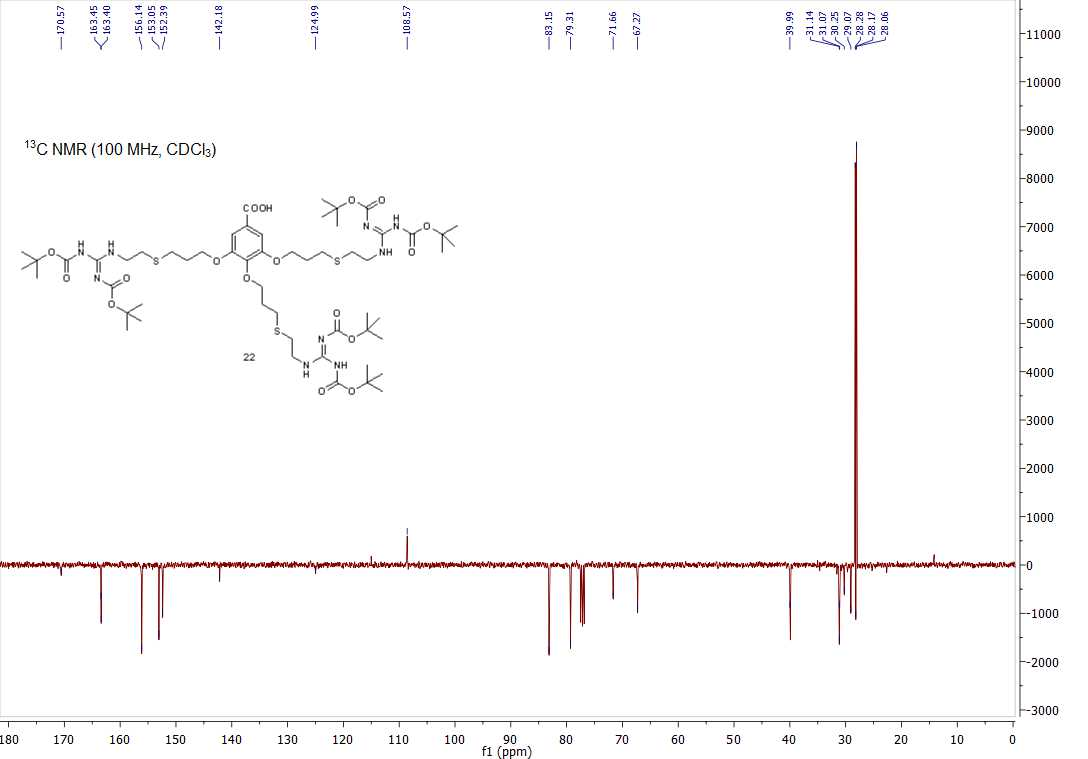


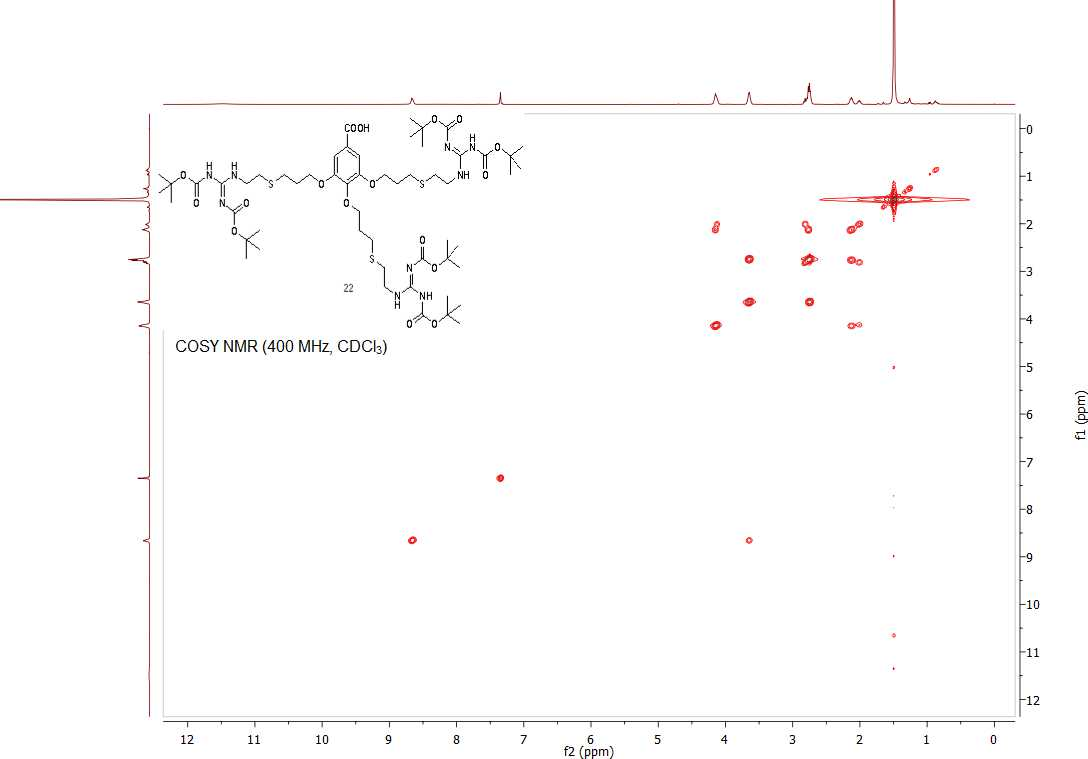


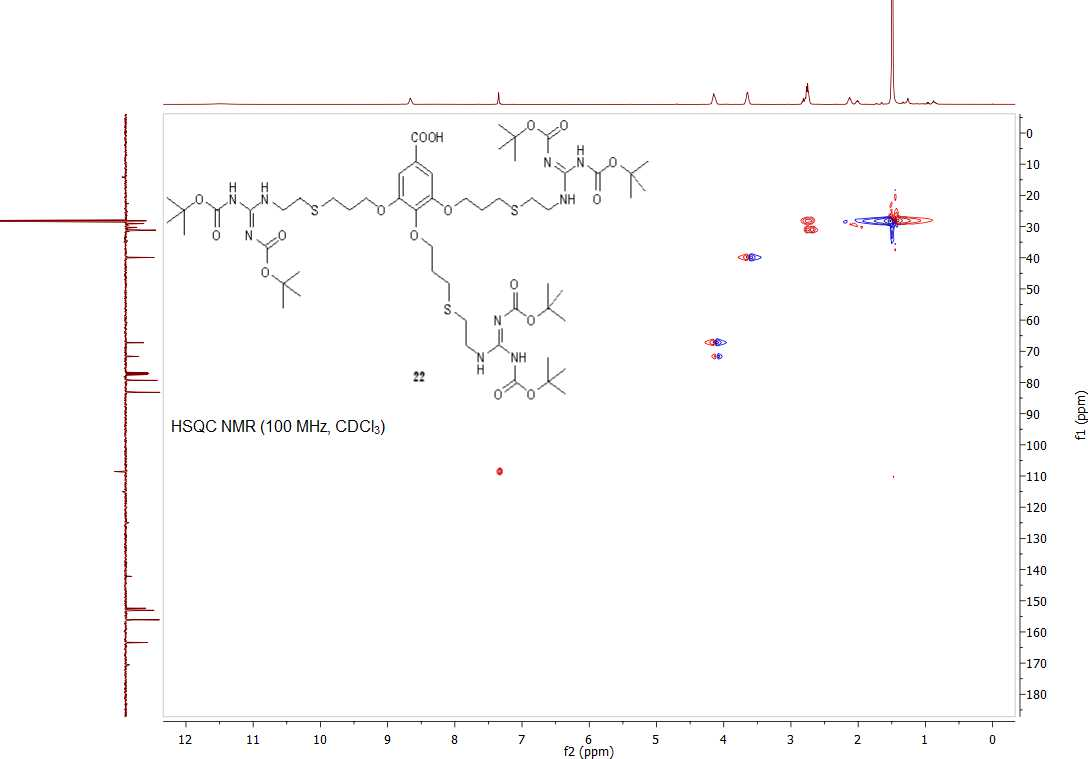


## NMR spectra of **23**


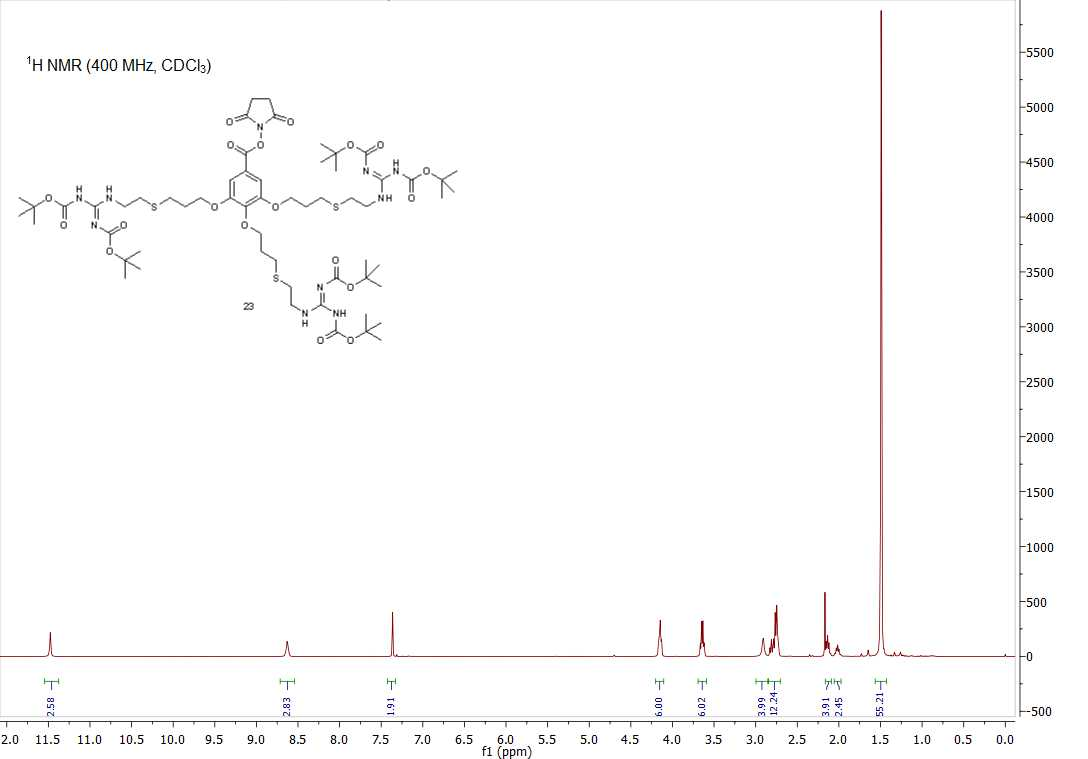


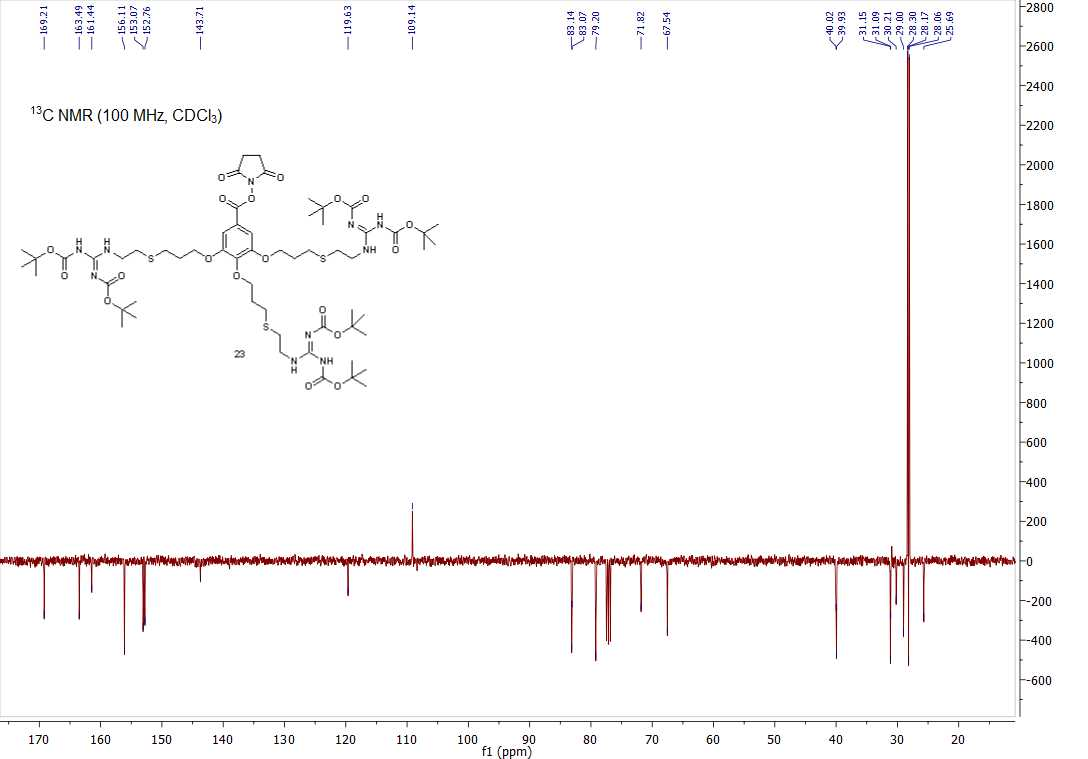


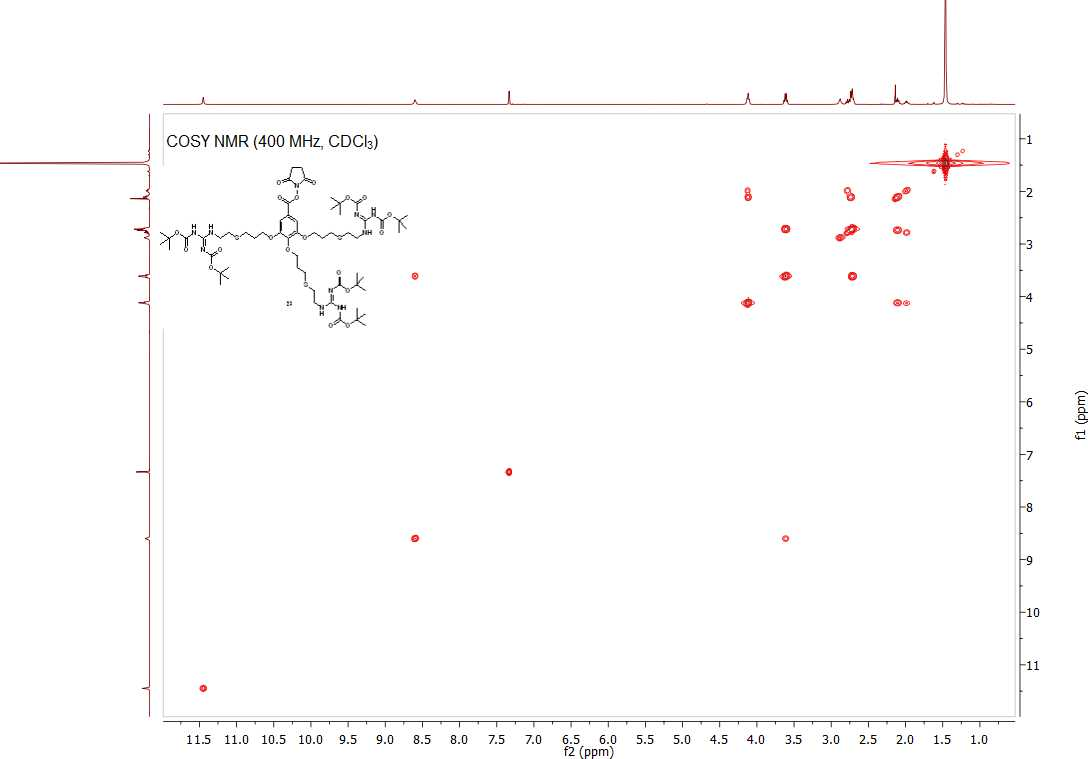


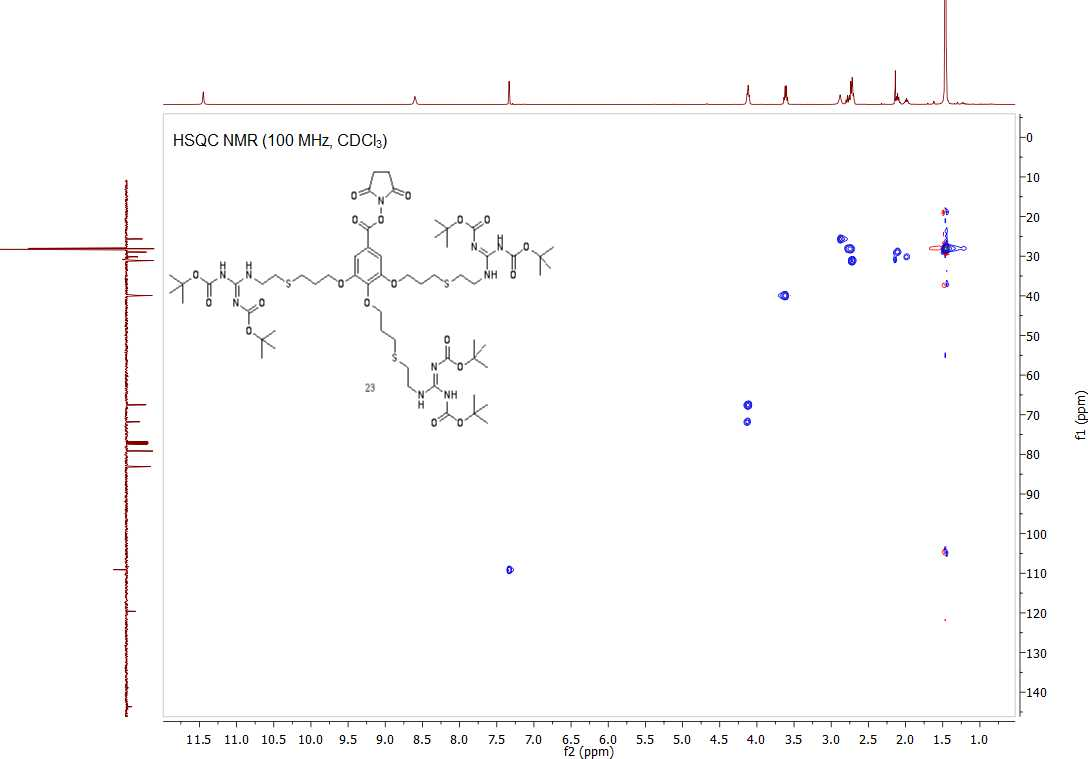


## NMR spectra of **24**


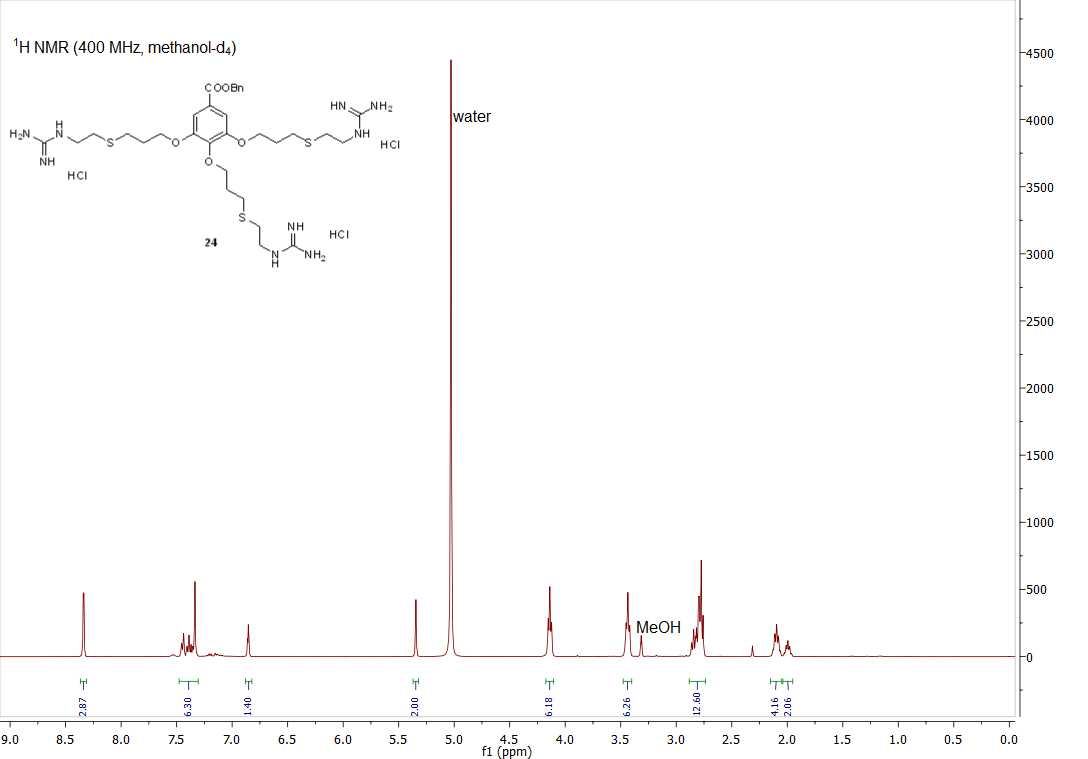


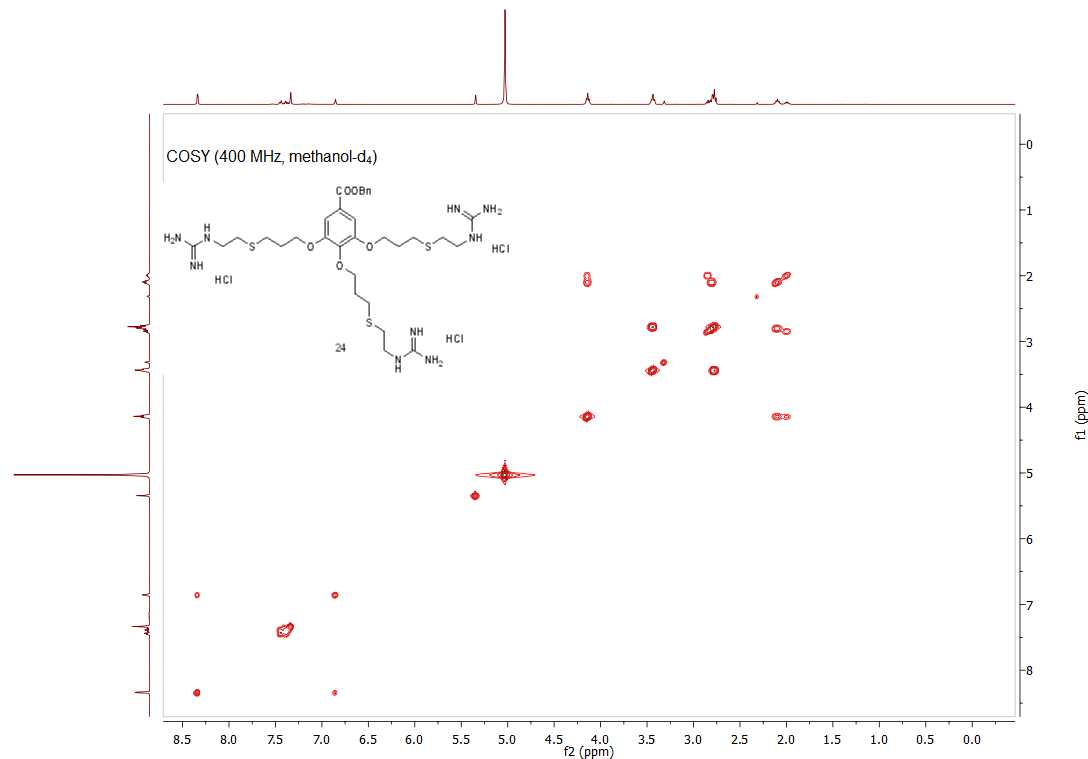


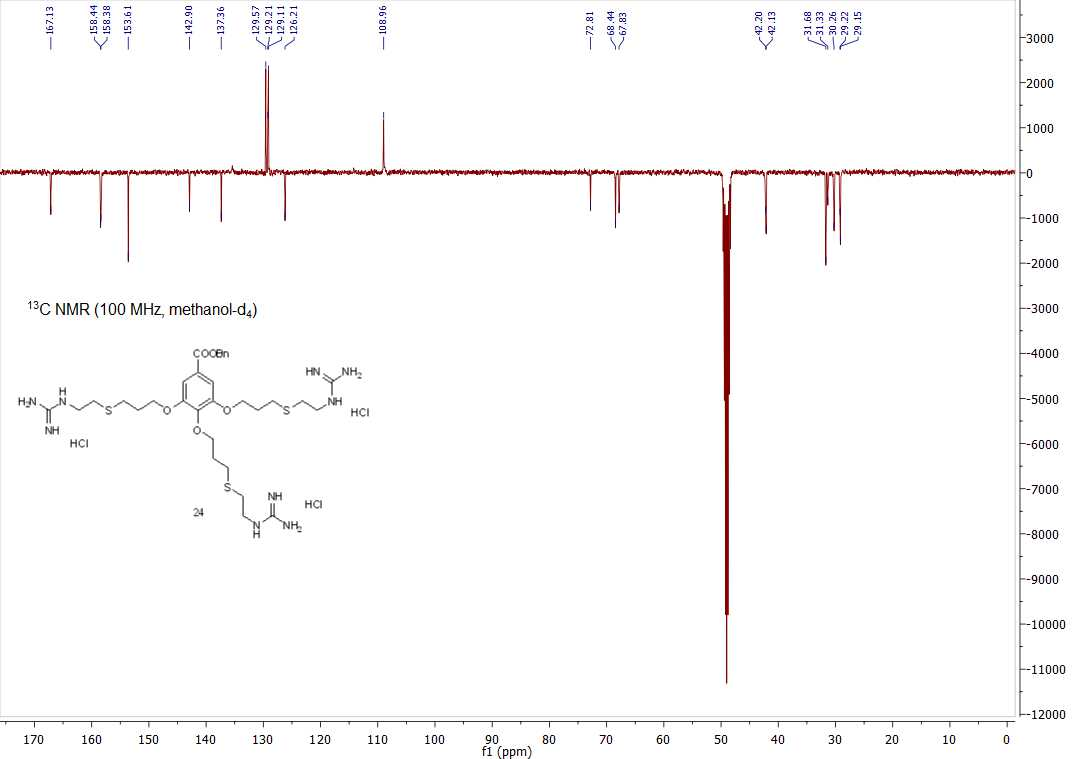

Supplement: Supplementary file 1 — Supplementary Information. [file 41598_2022_20182_MOESM1_ESM.docx]
